# Astilbin exerts a neuroprotective effect by upregulating the signaling of nuclear NF-E2-related factor 2 *in vitro*

**DOI:** 10.1016/j.heliyon.2024.e37276

**Published:** 2024-09-03

**Authors:** Chao Guo, Ying Yin, Zhongying Ma, Fangqin Xu, Shiquan Wang

**Affiliations:** a Xi'an People's Hospital (Xi'an Fourth Hospital), Xi'an, China; b Department of Pharmacy, Xijing Hospital, Fourth Military Medical University, Xi'an, China; c Department of Anesthesiology, Xijing Hospital, Fourth Military Medical University, Xi'an, China

**Keywords:** Astilbin, Oxidative stress, Nrf2, Oxygen and glucose deprivation, Microscale thermophoresis

## Abstract

**Objective:**

The present study aims to evaluate the impact of Astilbin (AST) on cortical neuron survival *in vitro* under conditions of oxygen-glucose deprivation and reoxygenation (OGD/R) and determine the role of NF-E2-related factor 2 (Nrf2) in this process.

**Methods:**

Primary neurons were pre-treated with various concentrations of AST for 8 h before OGD induction. Cell viability and lactate dehydrogenase (LDH) leakage were assessed to determine the optimal concentration. Biomarkers related to oxidative stress, antioxidant enzyme activities, and apoptosis were evaluated at 24 h post-OGD/R. To investigate the involvement of Nrf2 in AST-mediated neuroprotection, we conducted molecular docking and microscale thermophoresis analyses, as well as examined the expression levels of Nrf2 and its regulatory genes including heme oxygenase-1(HO-1), (NAD(P)H: quinone oxidoreductase 1 (NQO-1), and peroxiredoxin 1 (Prdx1). Additionally, lentivirus-mediated knockdown of Nrf2 and overexpression of Nrf2 with L-sulforaphane (SFN) were performed, followed by an assessment of cell viability, oxidative stress, antioxidant enzyme activities and apoptosis.

**Results:**

Pre-treatment with AST reduced oxidative stress levels while increasing antioxidant enzyme activities and mitigating neuronal apoptosis. After OGD/R exposure, AST upregulated nuclear Nrf2 expression and increased the expression of HO-1, NQO-1 and Prdx1 in the cytoplasm. However, the knockdown of Nrf2 abolished the antioxidative and protective effects exerted by AST treatment. Conversely, combining AST with the Nrf2 agonist SFN demonstrated an enhancement in the protective effects provided by AST. These results demonstrate that Nrf2-dependent antioxidant responses contribute to AST-induced tolerance against neuronal injury caused by OGD/R injury.

**Conclusions:**

Overall findings support the ability of AST to protect primary neurons from OGD/R-induced damage through activation of Nrf2-dependent antioxidant responses.

## 1 Introduction

Cerebral ischemia/reperfusion (CI/R) is characterized by inadequate cerebral blood flow and subsequent impaired blood flow recovery, posing significant challenges as a global health issue [1]. The severe consequences of ischemic stroke, including cognitive and motor dysfunction, as well as neurodegenerative diseases, impose substantial burdens on patients' families and society at large [2,3]. Neuronal death represents a pivotal pathological event during the progression of ischemia-induced impairments [4]. Consequently, numerous studies have focused on elucidating the underlying mechanisms of neuronal death induced by ischemia and exploring potential interventions. It is widely recognized that the generation of reactive oxygen species (ROS) plays a crucial role in the pathophysiological processes leading to neuronal death following ischemic events [5,6]. Excessive production of ROS directly disrupts lipids, proteins, and nucleic acids within cells, resulting in cell demise [7].

Nuclear factor erythroid 2-related factor (Nrf2), as a transcription factor, regulates many genes involved in ROS detoxification and antioxidative reactions [8]. The binding of Nrf2 to Keap1, a protein associated with Kelch-like ECH, typically serves to restrict its activity. However, under conditions of oxidative stress, Nrf2 separates from Keap1 and relocates to the nucleus. Once in the nucleus, Nrf2 interacts with the antioxidant response element (ARE) and triggers the activation of various antioxidant enzymes including catalase (CAT), glutathione peroxidase (GSH-PX), and superoxide dismutase (SOD). Additionally, it regulates the expression of certain phase II detoxification enzymes such as NQO1, HO-1, and Prdx1. Numerous studies have provided evidence that enhancing nuclear levels of Nrf2 can mitigate oxidative damage caused by ischemia [9,10].

Astilbin (AST), a flavonoid compound derived from the *rhizome of Smilax china* L., has been found to possess antioxidative, anti-inflammatory, and anti-apoptotic properties [11,12]. AST may have a positive impact on mice with Parkinson's disease induced by MPTP, as it can suppress gliosis, overexpression of α-synuclein, and oxidative stress [13]. AST mitigates damage caused by cerebral ischemia/reperfusion through the suppression of the TLR4/MyD88/NF-κB signaling pathway [14]. Recent research has also reported that AST has the potential to protect against cerebral I/R injury by suppressing apoptosis and inflammation through inhibition of the MAPK pathway while activating the AKT pathway [15]. These findings strongly suggest that AST could potentially offer neuroprotective effects in diseases affecting the nervous system. However, the protective mechanism of AST against ischemic neuronal injury remains uncertain. Further investigation is necessary to explore this aspect. Therefore, the objective of this study was to evaluate the influence of AST on the survival of cortical neurons *in vitro* during oxygen-glucose deprivation and reoxygenation (OGD/R), while also examining the role played by Nrf2 in this process.

## 2 Materials and methods

### 2.1 Reagents

Astilbin (molecular weight of 450.39, >99 % purity; CAS 29838-67-3) was supplied by the Weikeqi Biotech Co., Ltd (Chengdu, China). Fetal bovine serum (FBS), Dulbecco's modified Eagle's medium (DMEM)/Ham's F12, B27 and L-glutamine were provided by Gibco (USA). Cell Counting Kit-8 (CCK-8, Cat: C0038), ROS assay (Cat: S0033S) and TUNEL apoptosis assay (Cat: C1088) kits were obtained from Beyotime (China). Protein extraction (Cat: W038-1-1), BCA protein assay (Cat: A045-4-2), 4-hydroxynonenal (4-HNE, Cat: H268-1-2) and 8-hydroxy-2′-deoxyguanosine (8-OHdG, Cat: H165-1-2) ELISA, superoxide dismutase (SOD, Cat: A001-3-2), catalase (CAT, Cat: A007-1-1), glutathione peroxidase (GSH-PX, Cat: A005-1-2) and lactate dehydrogenase (LDH, Cat: A020-2-2) assay kits were from Jiancheng Bioengineering Institute (Nanjing, China). L-sulforaphane (CAS:142825-10-3) was provided by TargetMol Chemicals Inc (Shanghai branch, China). Anti-cleaved caspase-3(ab214430), anti-HO-1(ab189491), anti-NQO1(ab80588), anti-histone H3 (ab1791), anti-Nrf2(#41255), anti-Prdx1(#32463) and anti-β-actin (#48139) were manufactured by Abcam (Cambridge, UK) and SAB (Maryland, U.S.A).

### 2.2 Primary cortical neuron cultures

The mice utilized in this investigation were acquired from the Fourth Military Medical University Laboratory Animal Center. All animal experiments were approved by the Institutional Animal Care and Utilization Committee of the Fourth Military Medical University, with ethics approval number IACUC-20190059. Primary cortical neurons were isolated from embryonic day-18 (E18) C57BL/6 mouse brain samples following previously established protocols [16]. The euthanasia procedure for the mice adhered to the ARRIVE guidelines [17]. Briefly, pregnant C57BL/6 mice were anesthetized using isoflurane (3 % for induction and 1.5 % for maintenance), and then the embryos were promptly extracted. The dissection process involved a 10-min digestion with 0.125 % trypsin, followed by the collection of cells using a DMEM medium supplemented with 10 % FBS. Once cellular attachment to the flask's bottom occurred, they were cultured in a neurobasal medium containing 2 % B27 and 1 % glutamax. Medium changes took place every three days to sustain cell viability and growth.

### 2.3 Oxygen-glucose deprivation and reoxygenation (OGD/R)

Primary neurons were washed 3 times in DMEM without glucose, aspartate, glutamine, or B27, and then incubated in the same medium. Next, the cells were placed in an incubation chamber flushed with 95 % N_2_/5 % CO_2_ (2 L/min) for 15 min at ambient temperature to remove oxygen. After sealing, the chamber was transferred into a 37 °C incubator for 60 min. Upon OGD treatment, primary neurons were placed in a normal medium.

Primary neurons were pre-treated with different concentrations of AST in the same volume for 8 h before OGD. DMSO was employed to dissolve AST to yield a 100 μM stock solution, which was subsequently diluted with a culture medium.

### 2.4 Grouping

For evaluating the optimal concentration of AST against OGD/R injury, the cells were divided into the following groups: control (ctrl), OGD/R, OGD/R + DMSO and OGD/R + AST groups. The control group had no treatment. The OGD/R group only underwent OGD/R treatment. The OGD/R + DMSO group was treated with DMSO and the OGD/R + AST groups were administered 1, 5, 10, 20 and 40 μM AST, respectively, for 8 h before OGD. To further assess the antioxidant effects of AST in OGD/R-induced injury, the cells were divided into 4 groups: control (ctrl), control (ctrl) + AST, OGD/R, and OGD/R + AST groups. The control group received no treatment. The control + AST group solely underwent AST treatment without exposure to OGD/R. The OGD/R group exclusively underwent OGD/R treatment. The OGD/R + AST group was administered 20 μM AST (the optimal concentration). To determine the role of Nrf2 in the neuroprotective effects of AST, the cells were divided into 5 groups: AST, lentivirus control (Lv-ctrl) + AST, and lentivirus-Nrf2 (Lv-Nrf2) + AST, SFN (Nrf2 activator), SFN + AST groups. The Lv-ctrl + AST and Lv-Nrf2+AST groups underwent transfection with control and Nrf2-knockdown lentiviruses for 72 h, respectively, followed by treatment with 20 μM AST for 8 h. The SFN group was treated with a concentration of 5 μM SFN, while the SFN + AST group received a combination treatment of 5 μM SFN and 20 μM AST, followed by exposure to OGD/R for 24 h.

### 2.5 Cell viability and LDH activity assessment

Cell viability was evaluated using a commercially available nonradioactive cell counting kit (CCK-8) assay. The absorbance of each well in the culture plate was measured at 450 nm using a microplate reader (Thermo Scientific, USA). Cell viability was expressed as a percentage relative to the control group, which was considered 100 %. LDH activity was determined by following the protocol provided by the manufacturer's LDH cytotoxicity detection kit. Absorbance measurements were taken at 492 nm, with background signals acquired at 655 nm.

### 2.6 TUNEL staining

Apoptosis assessment was conducted using the Terminal deoxynucleotidy1 transferase-mediated dUTP nick end-labelling (TUNEL) technique. Following 24 h of OGD/R, cells were incubated with TdT and dUTP labelled with fluorescein for 45 min at a temperature of 37 °C. Analysis was performed using an inverted fluorescence microscope from Olympus, Japan. The presence of green dots in each field of observation indicated TUNEL-positive cells. This experiment was replicated three times.

### 2.7 Mitochondrial ROS detection

ROS amounts were assessed with the ROS assay kit as described by the manufacturer. Fluorescence signals were detected on a fluoro-spectrophotometer with excitation at 488 nm and emission at 525 nm.

### 2.8 Measurement of antioxidant enzymes

The cells were homogenized in chilled sodium chloride. The homogenates underwent centrifugation and supernatants were collected and frozen at −80°Cfor future analysis. Protein content in the supernatant was assessed by the BCA method. SOD, CAT and GSH-PX enzyme activities were assessed with specific assay kits.

### 2.9 4-HNE and 8-OHdG detection

Twenty-four hours after OGD, the culture media were collected and frozen at −80 °C in different groups. Then, specific ELISA assay kits were used to measure the contents of 4-HNE and 8-OHdG, as directed by the manufacturer.

### 2.10 Molecular docking and microscale thermophoresis

The affinity between AST and Nrf2 was observed using Schrodinger software. The protein structure of Nrf2 was predicted from the AlphaFold2. The active sites of AST and Nrf2 protein were analyzed by MM-GBSA calculation. MM-GBSA dG Bind can approximately represent the binding free energy of compound and protein. The lower the binding free energy, the higher the binding stability of ligands and proteins. The microscale thermophoresis (MST) experiments were conducted using a specialized instrument called Monolith NT.115 from NanoTemper Technologies. The Nrf2-purified protein was labelled with the Monolith™ RED-NHS 2nd Generation kit and diluted in NHS-labelled buffer. AST underwent an incubation process alongside Nrf2 proteins, followed by absorption using capillaries to assess their binding affinity. The fluorescence measurement was performed using a Monolith NT.115 instrument from NanoTemper Technologies, with the LED power set at 40 % and high MST power in a thermal gradient. The data analysis was carried out utilizing affinity analysis v2.3 software.

#### 2.10.1 Immunofluorescence

Cultured primary neurons were fixed for 10 min with 4 % paraformaldehyde at ambient temperature. Upon blocking with 5 % BSA in phosphate-buffered saline (PBS), the cells underwent successive incubations with primary (24 h at 4 °C) and secondary (1:500; 2 h at 37 °C) antibodies. Subsequently, DAPI was used for counterstaining (5 min at ambient temperature). Primary antibodies were anti-Nrf2 (1:100) and anti-MAP-2 (1:300).

#### 2.10.2 Cell transfection

Small interfering RNA (shRNA) for Nrf2 knockdown was transfected into primary neurons with a lentiviral system. Transfection efficiency was assessed by immunoblot. Following 72 h of transfection, primary neurons underwent pretreatment with AST and were submitted to OGD (1 h) and reoxygenation (24 h). Cell specimens were obtained to assess the cell viability and antioxidative ability.

#### 2.10.3 Western blotting

Western blot analysis was performed according to the standard protocols. Cytoplasm and nuclear proteins from primary neurons were extracted with a homogenization buffer containing a protease inhibitor. The BCA method was utilized for protein content assessment. Equal amounts of protein were resolved by 10–15 % SDS-PAGE and transferred onto polyvinylidene difluoride (PVDF) membranes. Then, 5 % BSA was used for 1 h blocking, followed by successive incubations with primary antibodies against cleaved caspase-3, HO-1, NQO1, Prdx1, β-actin, Nrf2 and histone H3 at 4 °C overnight, respectively, and horseradish peroxidase-linked secondary antibodies for 90 min. A Chemi Doc™ system (Bio-Rad, USA) was employed for visualization.

#### 2.10.4 Statistical analysis

Data are presented as means with standard deviation (SD). One-way analysis of variance (ANOVA) was performed to assess multiple groups. SPSS 10.0 was employed for data analysis. All experiments were carried out at least three times independently. *P* < 0.05 indicated statistical significance.

## 3 Results

### 3.1 AST decreases neuronal injury induced by OGD/R

**Fig. 1:**
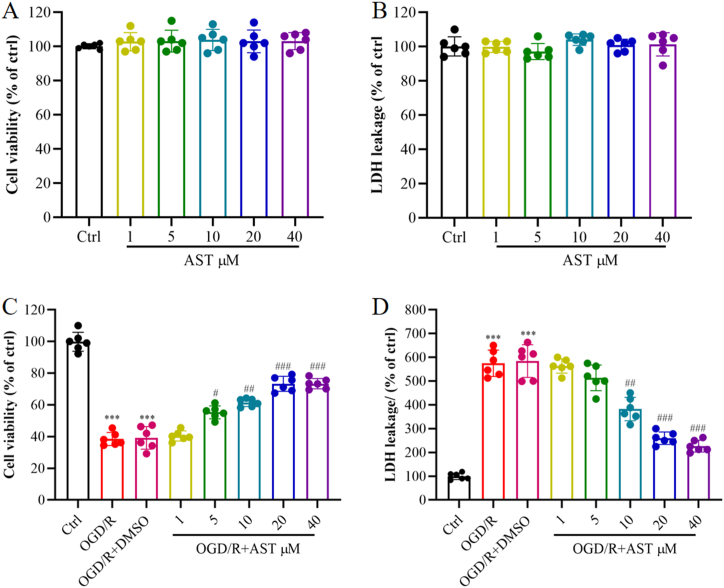
Effects of various concentrations of AST on cell viability and LDH release in primary cortical neurons. (A) Cell viability was assessed in the absence of OGD/R. (B) LDH release was quantified in the absence of OGD/R. (C) Cell viability was measured in the presence of OGD/R. (D) LDH release was quantified in the presence of OGD/R. Data were presented as mean ± SD (n = 6). ***P < 0.001 compared to the control (ctrl) group; ^###^*P* < 0.001, ^##^*P* < 0.01 and ^#^*P* < 0.05 compared to the OGD/R group.

Cell viability and LDH activity induced by primary neurons pretreated with AST and exposed to OGD for 1 h followed by reoxygenation for 24 h were quantitated. As shown in Fig. 1A and B, the results suggested that the viability and LDH levels of normal cells remained unaffected by different concentrations of AST. Subsequently, the impact of different concentrations of AST on cells exposed to OGD/R was examined. As shown in Fig. 1C, the cell viability experienced a notable decrease under OGD/R conditions in comparison to the control group (*P* < 0.001). However, when pre-treated with different concentrations of AST (5, 10, 20, 40 μM), there was a significant enhancement in cell survival compared to the OGD/R group (*P* < 0.05 or *P* < 0.01 or *P* < 0.001). According to the cell viability assay, AST exhibited a concentration-dependent ability to safeguard cultured cells from OGD/R-induced injury.

As shown in Fig. 1D, LDH levels were found to be significantly low in the control group. However, the OGD/R and OGD/R + DMSO groups exhibited a significant increase in LDH levels (*P* < 0.001). Pretreating with AST at concentrations of 1 μM and 5 μM resulted in a slight reduction in LDH release, without any statistically significant difference compared to the OGD/R group. Conversely, there was a remarkable inhibition of LDH release observed in the 10 μM, 20 μM and 40 μM AST groups, with statistically significant differences when compared to the OGD/R group (*P* < 0.01 or *P* < 0.001). Notably, the effect of AST at a concentration of 20 μM was more pronounced than that at 10 μM. Furthermore, there was no significant difference observed between the LDH release levels of the 40 μM AST group and those of the 20 μM AST group. At the concentration of 20 μM, AST provided better protection from OGD/R-induced cell injury; thus, 20 μM AST was used in subsequent experiments. In addition, we observed no impact of DMSO on the cells, thus a separate control group for the solvent was not established in the subsequent experiment.

### 3.2 AST inhibits oxidative stress injury and enhances antioxidant enzyme activities

**Fig. 2:**
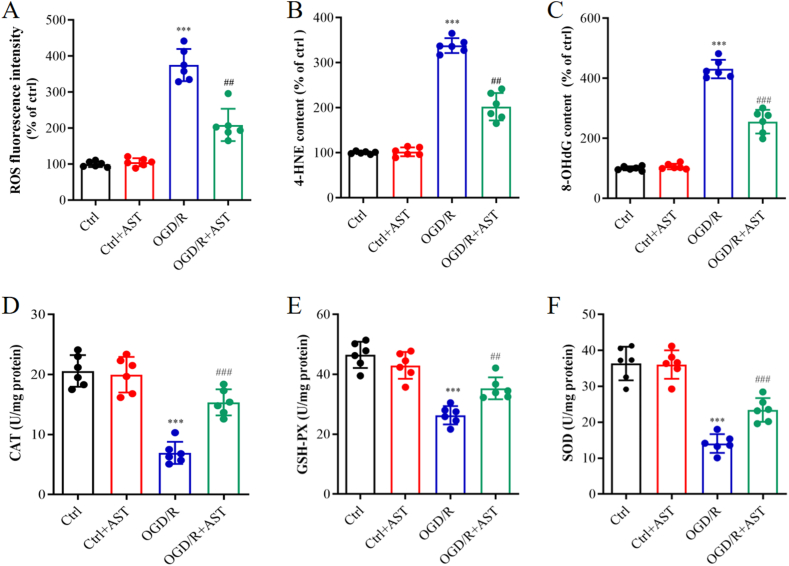
AST alleviated the oxidative stress and enhanced the antioxidant enzymes after OGD exposure for 1 h followed by reoxygenation for 24 h. (A–C**)** AST decreased the content of ROS, 4-HNE and 8-OHdG. (D–F**)** AST increased the activities of CAT, GSH-PX and SOD. Data were presented as mean ± SD (n = 6). ***P < 0.001 compared to the control (ctrl) group; ^###^*P* < 0.001 and ^##^*P* < 0.01 compared to the OGD/R group.

The antioxidative effects of AST in primary neurons after OGD/R were evaluated by measuring oxidative stress biomarkers, including ROS, 4-HNE and 8-OHdG. Compared to the control group, the OGD/R group exhibited elevated levels of ROS, 4-HNE and 8-OHdG (*P* < 0.001). However, pre-treatment with AST significantly reduced the levels of ROS, 4-HNE and 8-OHdG (Fig. 2A–C, *P* < 0.01 or *P* < 0.001). To further evaluate the antioxidative effects of AST on oxidative injury induced by OGD/R, we assessed the activities of CAT, GSH-PX and SOD. Compared to the control group, the OGD/R group exhibited decreased activities of CAT, GSH-PX, and SOD with significant differences. However, administration of AST resulted in significant increases in the activities of these antioxidant enzymes compared to both the OGD/R group (Fig. 2D–F, *P* < 0.01 or *P* < 0.001).

### 3.3 AST decreases OGD-induced neuron apoptosis

**Fig. 3:**
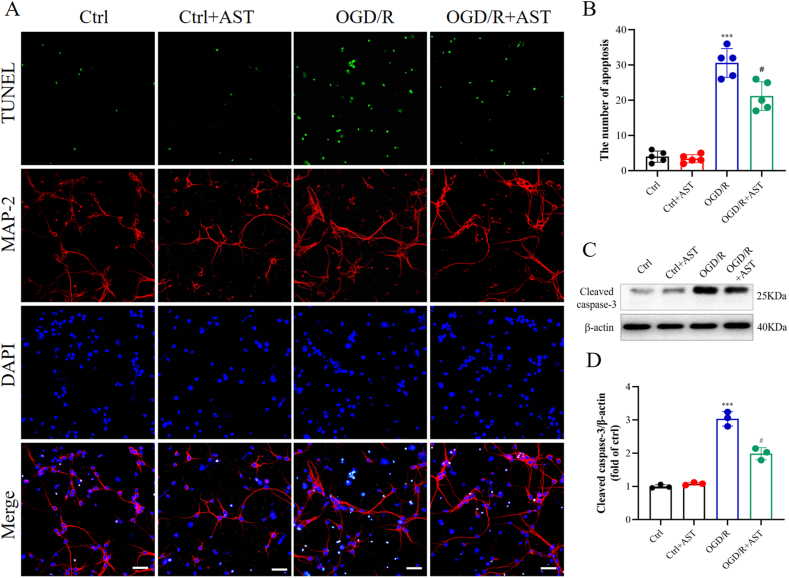
AST attenuated the cellular apoptosis after OGD exposure for 1 h followed by reoxygenation for 24 h. (A**)** The representative figure of TUNEL staining. Scale bar = 50 μm. TUNEL-positive cells (green), MAP-2 (red), and DAPI (blue). (B**)** The statistics of TUNEL-positive cells in different groups. Data were presented as mean ± SD (n = 5). (C) The representative band of cleaved caspase-3. (D) The statistics of cleaved caspase-3 content in different groups. Data were presented as mean ± SD (n = 3). ***P < 0.001 compared to the control (ctrl) group; ^#^*P* < 0.05 compared to the OGD/R group.

To determine the effect of AST on neuron apoptosis induced by OGD/R, the number of TUNEL-positive cells, as well as cleaved caspase-3 levels, were assessed. The number of TUNEL-positive cells was markedly elevated upon OGD/R compared with the control cells (*P* < 0.001) but starkly reduced after pretreating with AST (Fig. 3A–B, *P*<0.05). Primary neurons exposed to OGD/R exhibited a significant increase in levels of cleaved caspase-3 compared to the control cells, demonstrating a statistically significant distinction (*P* < 0.001). Conversely, pretreating with AST resulted in the suppression of cleaved caspase-3 levels when compared to the OGD/R group (Fig. 3C–D, *P* < 0.05).

### 3.4 AST treatment induces Nrf2 nuclear translocation

**Fig. 4:**
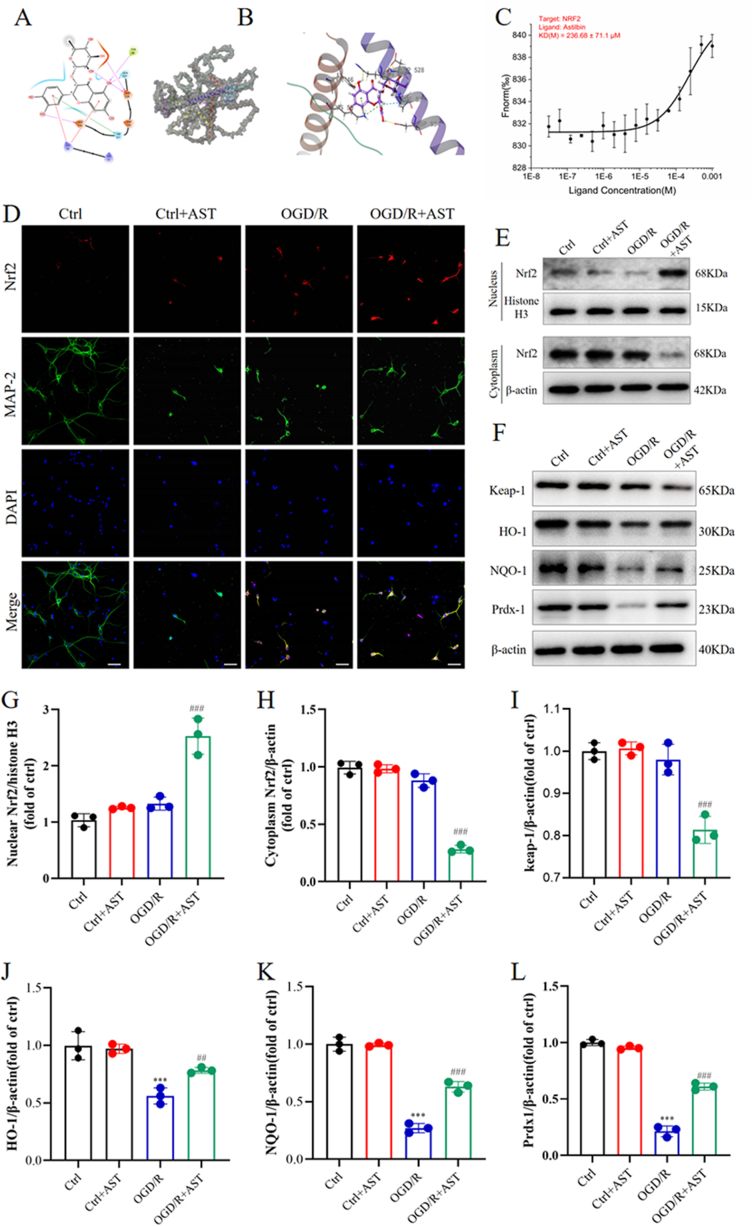
AST enhanced the expression levels of nuclear Nrf2, HO-1, NQO-1, and Prdx-1proteins after OGD exposure for 1 h followed by reoxygenation for 24 h. (A) Amino acid residues. (B) The side view of the molecule binding pocket. (C) Dose-response curve of AST binding to Nrf2 using MST. (D**)** The representative immunofluorescence staining of Nrf2 at 24 h after OGD/R. Scale bar = 50 μm. Nrf2 (red), MAP-2 (green), and DAPI (blue). (E)The representative brands of Nrf2 in the nucleus and cytoplasm. (F) The representative brands of keap-1, HO-1, NQO-1 and Prdx-1 in the cytoplasm. (G–L) Quantitative analysis of different protein expression at 24 h after OGD/R. Data were presented as mean ± SD (n = 3**)**. ***P < 0.001 compared to the control (ctrl) group; ^###^*P* < 0.001 and ^##^*P* < 0.01 compared to the OGD/R group.

To evaluate the role of Nrf2 in the neuroprotective effect of AST, molecular docking analysis was employed to confirm the binding interaction between AST and the Nrf2 protein. The docking score for AST with Nrf2 was determined to be −3.445, while the MM-GBSA result yielded a value of −23.88 kcal/mol (Fig. 4A–B). Both the low binding free energy and docking score suggest a stable association between AST and Nrf2. Subsequently, the binding of AST and Nrf2 was measured by MST assays, and the results confirmed that AST can directly bind with Nrf2 (KD = 236.68 ± 71.1 μM, Fig. 4C). Next, we conducted immunofluorescence analysis to investigate the intracellular distribution of Nrf2 in the nucleus. As shown in Fig. 4D, pretreatment with AST significantly elevated the expression of Nrf2 in primary neurons. Furthermore, Western blot analyses were performed to evaluate the levels of Nrf2 protein in both the nucleus and cytoplasm. Our results revealed that AST preconditioning resulted in an increase in nuclear-Nrf2 levels and a decrease in cytosolic Nrf2 levels compared to those observed in the OGD/R group (Fig. 4E–G and H, *P* < 0.001). Additionally, AST preconditioning also decreased Keap-1 levels while enhancing HO-1, NQO-1, and Prdx-1 levels, relative to that observed in the OGD/R group (Fig. 4F–I-L, *P* < 0.001).

#### 3.4.1 The protective effects of AST were dependent on Nrf2

**Fig. 5:**
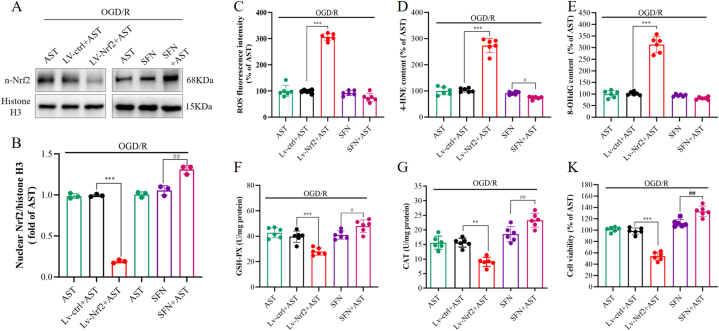
The effect of AST on oxidative stress and the activity of antioxidant enzymes was assessed by lentivirus-mediated knockdown or overexpression of Nrf2. (A) The efficiency test of knocking down and activating Nrf2 by Western blotting. (B) Quantitative analysis of Nrf2 expression. (C–E) The levels of ROS, 4-HNE and 8-OHdG under conditions of Nrf2 knockdown and activation. (F–K) The activities of GSH-PX and CAT under conditions of Nrf2 knockdown and activation. Data were presented as mean ± SD (n = 6). ***P < 0.001, **P < 0.001 compared to the Lv-ctrl + AST group; ^##^*P* < 0.01, ^#^*P* < 0.05 compared to the SFN group.

To validate the role of Nrf2 in neuroprotection induced by AST, we employed lentiviral technology and SFN to downregulate and upregulate the expression of Nrf2 in primary neurons, respectively. The efficiency of knockdown and overexpression of Nrf2 was initially assessed by Western blotting, which confirmed a significant reduction in Nrf2 levels following lentiviral-Nrf2 treatment and a substantial increase in Nrf2 expression after SFN administration (Fig. 5A and B, *P*<0.01 or *P* < 0.001). Subsequently, the markers of oxidative damage (ROS, 4-HNE, and 8-OHdG) as well as antioxidative enzymes (GSH-PX and CAT) were evaluated. In comparison to the Lv-ctrl + AST group, the Lv-Nrf2+AST group exhibited a significant elevation in levels of ROS, 4-HNE, and 8-OHdG (Fig. 5C–E, *P* < 0.001). Conversely, there was a significant decrease in the level of 4-HNE observed in the SFN + AST group compared to the SFN group (*P* < 0.05). Although no significant trend was observed for ROS and 8-OHdG levels in the SFN + AST group, they did demonstrate a decrease. Furthermore, GSH-PX and CAT activities were significantly reduced in the Lv-Nrf2+AST group when compared to the Lv-ctrl + AST group (Fig. 5F–G, *P* < 0.001). However, GSH-PX and CAT activities were significantly increased in the SFN + AST group compared to the SFN group (*P* < 0.05 or *P* < 0.01). As shown in Fig. 5K, cell viability results indicated that Nrf2 knockdown attenuated AST's protective effect on OGD/R-exposed cells while overexpression enhanced AST's protective effect.

**Fig. 6:**
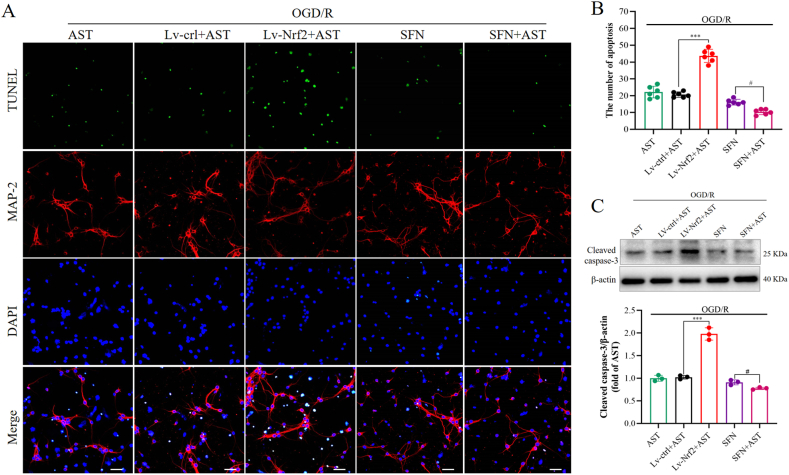
The protective effects of AST were dependent on Nrf2. (A)The representative TUNEL staining. Scale bar = 50 μm. Nrf2 (red), MAP-2 (green), and DAPI (blue). (B) The statistics of positive TUNEL cells in different groups. (C) Representative bands of cleaved caspase-3. (D) The statistics of cleaved caspase-3 levels in different groups. Data were presented as mean ± SD (n = 3). ***P < 0.001 compared to the Lv-ctrl + AST group; ^#^*P* < 0.05 compared to the SFN group.

Finally, we also investigated the impact of Nrf2 knockdown and overactivation on neuronal apoptosis and the level of cleaved caspase-3 (Fig. 6A–C). The Lv-Nrf2+AST group demonstrated a substantial increase in the number of TUNEL-positive cells compared to the Lv-ctrl + AST group (*P* < 0.001). Cleaved caspase-3 levels were significantly elevated in the Lv-Nrf2+AST group compared to the Lv-ctrl + AST group (*P* < 0.001). Conversely, both the number of TUNEL-positive cells and the level of cleaved caspase-3 were notably reduced in the SFN + AST group when compared with the SFN group (*P* < 0.05). These findings suggest that AST's protective effect is linked to the activation of the Nrf2 signaling pathway.

## 4 Discussion

Ischemia deprives of oxygen and nutrients, causing cell damage and/or death via the oxidative-related pathway [18,19]. Reperfusion of the ischemic tissue yields huge amounts of reactive oxygen and nitrogen species, disrupting redox signaling and ultimately causing neuron damage and apoptosis [20]. Accumulating evidence suggests that oxidative stress suppression might help attenuate I/R injury [[21], [22], [23]]. *In vitro*, the OGD/R model is commonly used to simulate I/R injury. Indeed, OGD/R is cytotoxic and promotes oxidative stress and apoptosis in neurons. Thus, we selected the OGD/R model of primary neurons in this work. We found that OGD/R caused primary neuron injury and decreased antioxidant enzyme activities. Although OGD/R-associated injury also slightly induced the expression of nuclear Nrf2 in primary neurons to resist oxidative damage, the subsequent accumulation of oxidative products overwhelmed cellular self-defense. Up-regulation of Nrf2 expression by natural antioxidant compounds is an effective approach for promoting cell survival. As mentioned above, preconditioning with AST alleviated neuronal injury, increased antioxidant enzyme activities, and further increased the levels of nuclear Nrf2.

Oxidative stress has a critical function in damage insults following brain ischemia and OGD/R injury [[24], [25], [26]]. Accumulated ROS oxidize multiple biomolecules such as proteins, membrane lipids and nucleic acids, causing cascade amplification of damage pathogenesis and leading to cell death. The main methods utilized to detect ROS include electron spin resonance (ESR) and DCFH-DA assays [27]. Of these methods, the DCFH-DA assay is considered the most convenient and feasible approach and is generally used for determining ROS levels [28,29]. 8-OHdG represents a predominant form of free radical-associated substance in oxidative injury and is therefore broadly utilized as an oxidative DNA damage marker [30], while 4-HNE is a product of lipid hydroperoxide degradation upon oxidative stress. 4-HNE can rapidly modify proteins on multiple amino acid moieties, causing protein inactivation [31]. In this study, ROS levels and 8-OHdG and 4-HNE contents were analyzed. Similar to published experimental results, ROS, 8-OHdG and 4-HNE amounts were increased after OGD/R. We demonstrated that AST decreased ROS, 8-OHdG and 4-HNE amounts. Intracellularly, anti-oxidative reactions are regulated by endogenous enzymatic antioxidants, including SOD, CAT and GPH-PX, which act jointly. It was reported that enhancing endogenous anti-oxidation by increasing the expression of antioxidants elicits a certain neuroprotective effect. This study also showed that AST treatment enhanced SOD, CAT and GSH-PX activities in primary neurons after OGD/R. These results illustrated that AST confers neuroprotection by increasing the activities of antioxidant enzymes.

Nrf2, a potent antioxidant and stress-responsive protein, is widely expressed in the mammalian nervous system [32]. Normally, Nrf2 and Keap1 form a complex at a low level of expression. However, when there is oxidative stress, Nrf2 separates from Keap-1 and facilitates the transportation of Keap-1 into the nucleus. This process contributes to the protective effects of Nrf2 against oxidative stress. The protective effects of Nrf2 in cerebral ischemia are well known [[33], [34], [35]]. In animal models, Nrf2 has overt beneficial effects on the brain, reducing infarct size, improving neurological scores, and decreasing cellular apoptosis after ischemia. Nrf2 downregulation increases MCAO-associated brain damage. It was reported that Nrf2 amounts peak at 24 h following MCAO or OGD/R [36]. However, endogenous Nrf2 does not increase sufficiently to protect neurons from OGD/R, and pharmacologically activating Nrf2 could potentially enhance neuronal survival following ischemia and OGD/R injury. Indeed, pharmacological activation of nuclear Nrf2 has been reported by multiple authors to enhance the protection of neurons from ischemia-associated injury and decrease the ischemic area [[37], [38], [39]]. Our research demonstrated that prior administration of AST led to a notable increase in the expression level of Nrf2 within neuronal nucleus at 24 h following OGD/R, while concurrently reducing its presence in the cytoplasm. These results indicate that under the OGD/R condition, AST may induce the translocation of Nrf2 from the cytoplasm to the nucleus. After Nrf2 transfers to the nucleus, the transcription of a series of antioxidant genes is activated. It has been verified that elevated levels of nuclear Nrf2 can lead to enhanced expressions of HO-1, NQO1, and Prdx-1 in the OGD/R model [[40], [41], [42]]. In line with these findings, our study also observed an increase in the expression of HO-1, NQO1, and Prdx-1 induced by AST, providing further evidence for the antioxidant role of AST through the up-regulation of nuclear Nrf2. To elucidate the role of Nrf2 in the protective effects associated with AST in OGD/R injury, a lentiviral system and L-Sulforaphane were employed to downregulate and upregulate Nrf2 expression. Our findings demonstrated that the knockdown of Nrf2 partially reversed the antioxidant function of AST and attenuated its protective effects; conversely, the overexpression of Nrf2 partially enhanced the antioxidant capacity of AST and strengthened its protective effect. The findings strongly indicate that the protective efficacy of AST is associated with the Nrf2 signaling pathway.

The limitations of the present study need to be addressed in the future. Firstly, it is imperative to demonstrate the in vivo protective effects of AST in CI/R injury and investigate the underlying mechanisms involved. Secondly, further investigation is necessary to determine the existence of upstream signaling pathways of Nrf2 that contribute to the protective effects of AST. Lastly, although we have confirmed the ability of AST to induce nuclear expression of Nrf2 and its specific affinity for Nrf2, the precise binding sites on Nrf2 and the phosphorylation mode responsible for increased Nrf2 expression remain unknown.

In conclusion, the effects of AST pretreatment in OGD/R-induced oxidative damage were demonstrated in the current study. Mechanistically, AST exerts its protective effects by facilitating the nuclear translocation of Nrf2 *in vitro*. These findings provide support for considering AST as a potential therapeutic candidate for ischemic stroke treatment.

## Ethics statement

This article does not contain any studies with human participants and the animal experiments were approved by the Fourth Military Medical University Institutional Animal Care and Utilization Committee, with ethics approval number IACUC-20190059.

## Funding

This present study was supported by grants from the key research and development plan of Shaanxi Province (2019SF-118) and 10.13039/501100002858China Postdoctoral Science Foundation (2022M722562).

## Data availability of statement

The data that support the findings of this study and supplementary information files for further data are available from the corresponding author upon reasonable request.

## CRediT authorship contribution statement

**Chao Guo:** Writing – review & editing, Writing – original draft, Supervision, Funding acquisition. **Ying Yin:** Writing – original draft, Software, Methodology. **Zhongying Ma:** Software, Methodology. **Fangqin Xu:** Software, Data curation. **Shiquan Wang:** Writing – review & editing, Supervision.

## Declaration of competing interest

The authors declare that they have no known competing financial interests or personal relationships that could have appeared to influence the work reported in this paper.
