# Supplementary material for: Astilbin exerts a neuroprotective effect by upregulating the signaling of nuclear NF-E2-related factor 2 in vitro
Source: Heliyon. 2024 Sep 3;10(17):e37276. doi: 10.1016/j.heliyon.2024.e37276 (PMC11409207; doi:10.1016/j.heliyon.2024.e37276)
Supplement: Multimedia component 1 [file mmc1.pdf]

Ctrl group

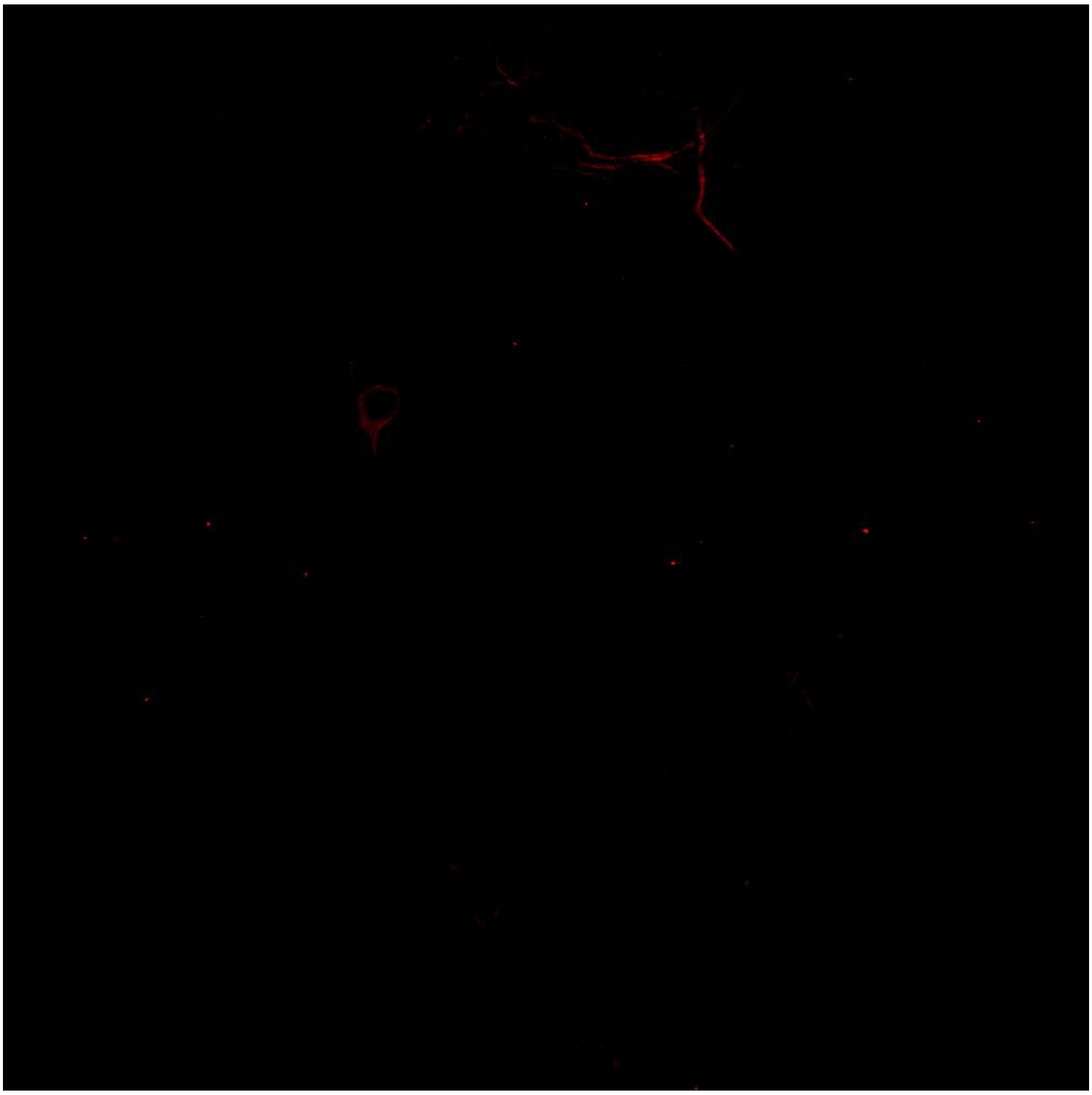

Nrf2

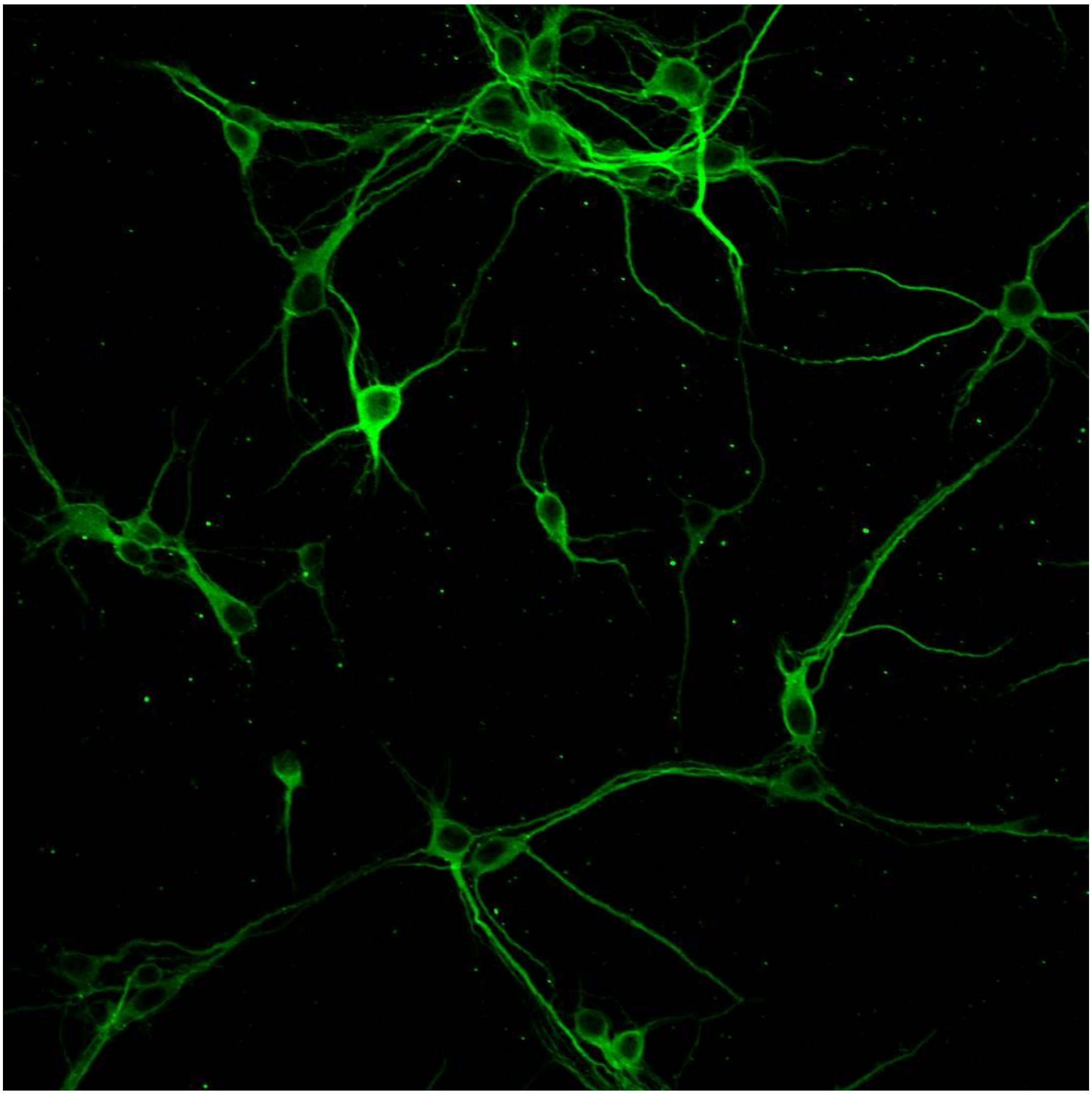

MAP-2

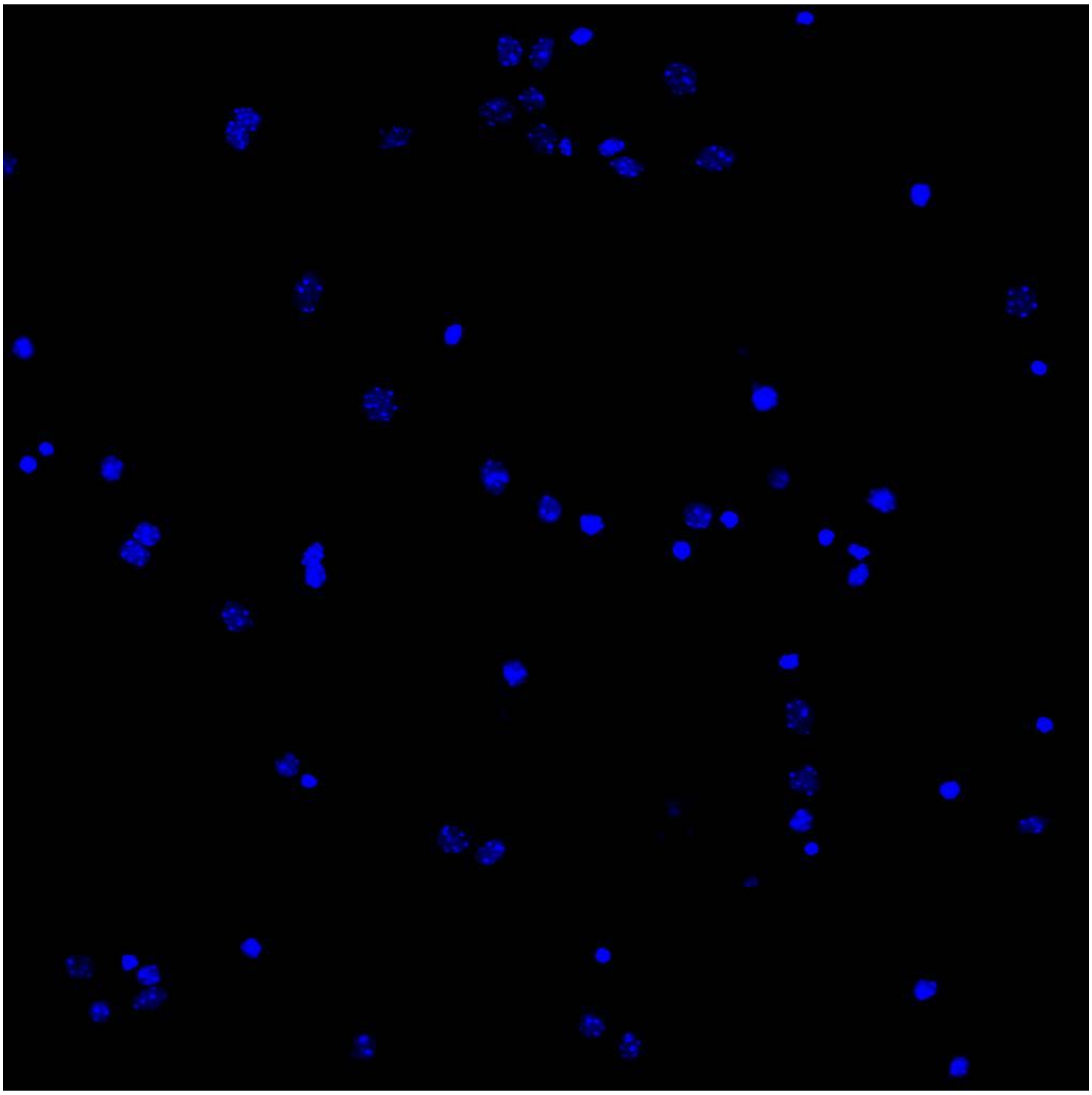

DAPI

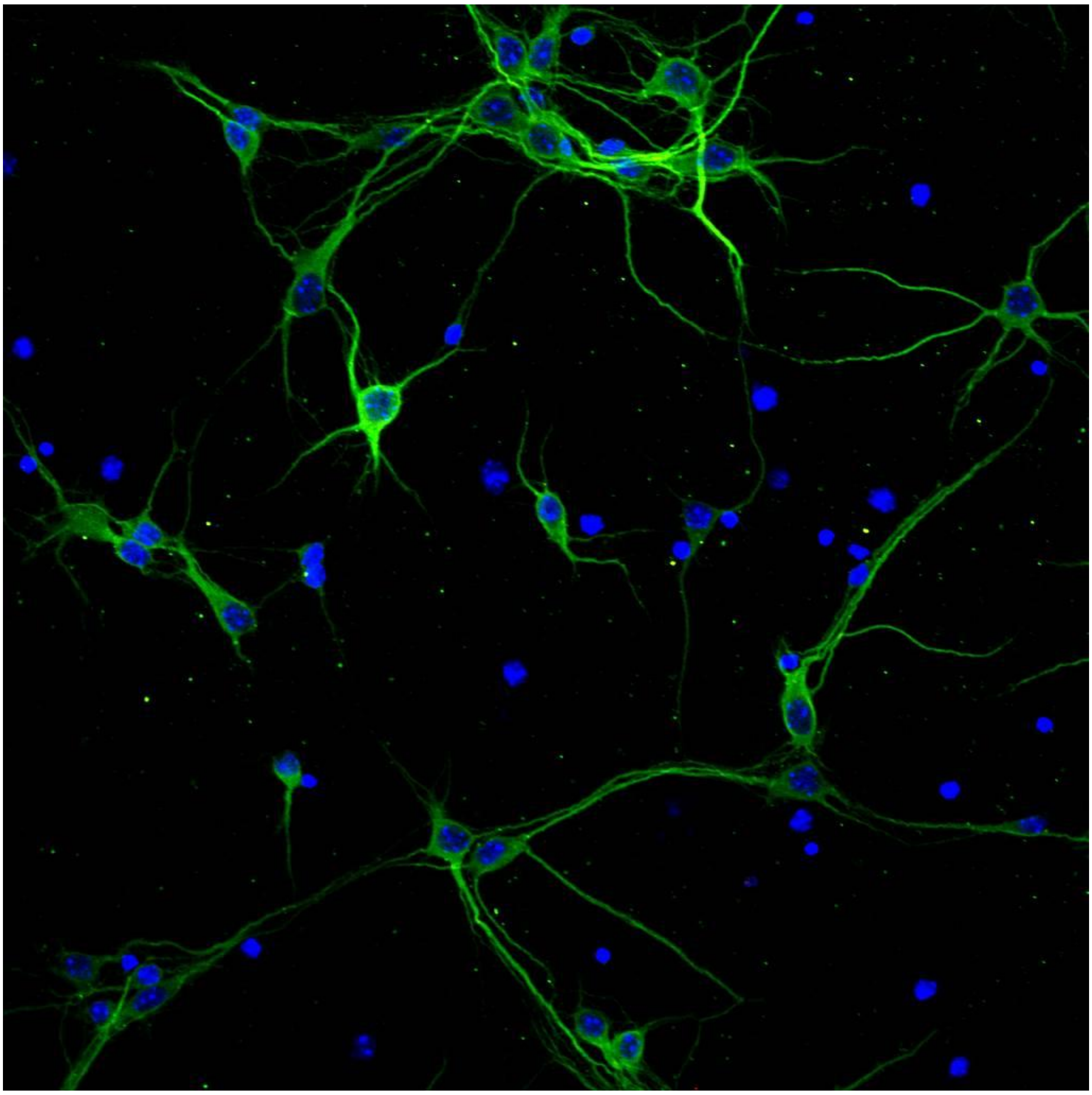

Merge

Ctrl+AST group

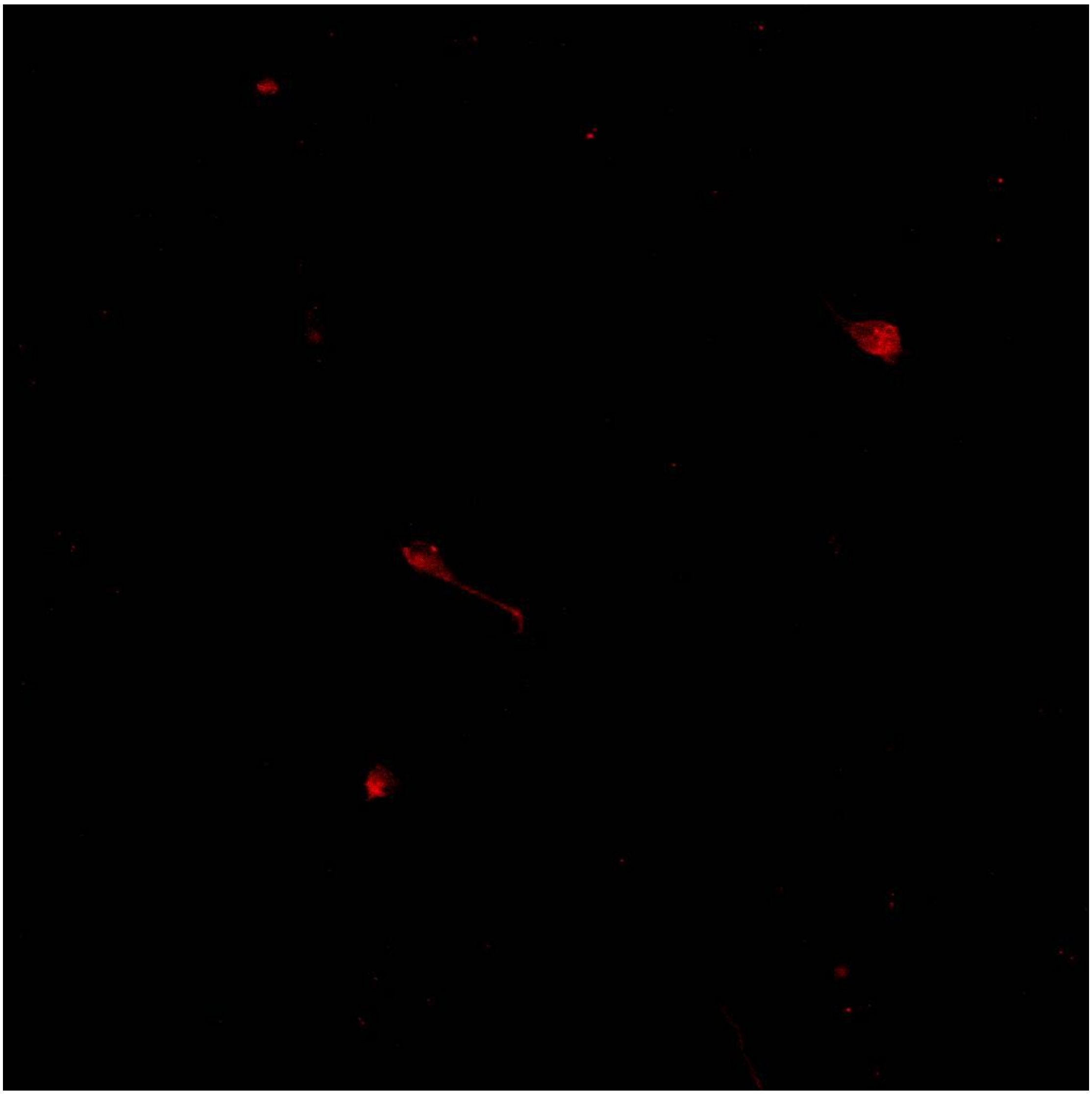

Nrf2

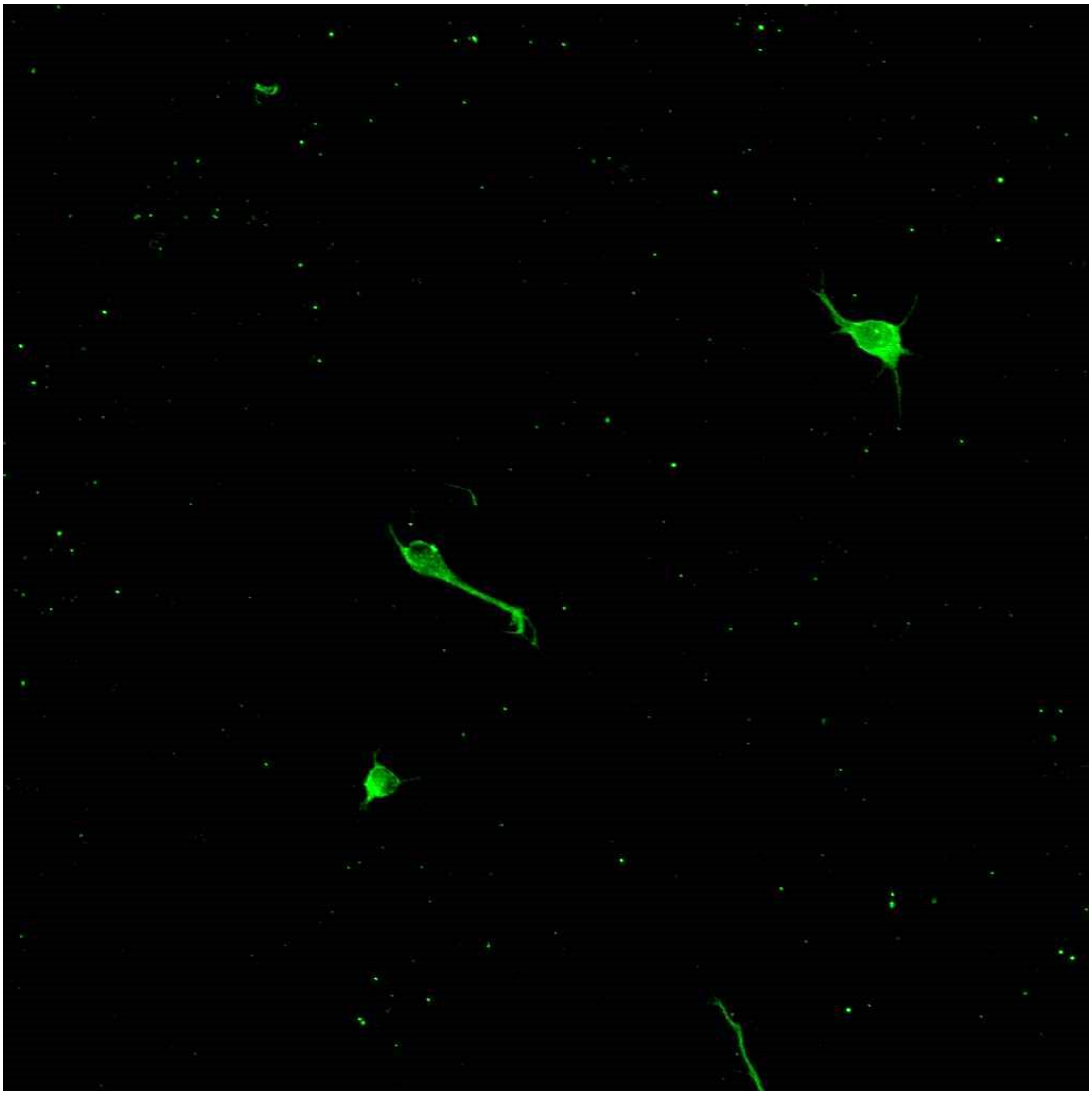

MAP-2

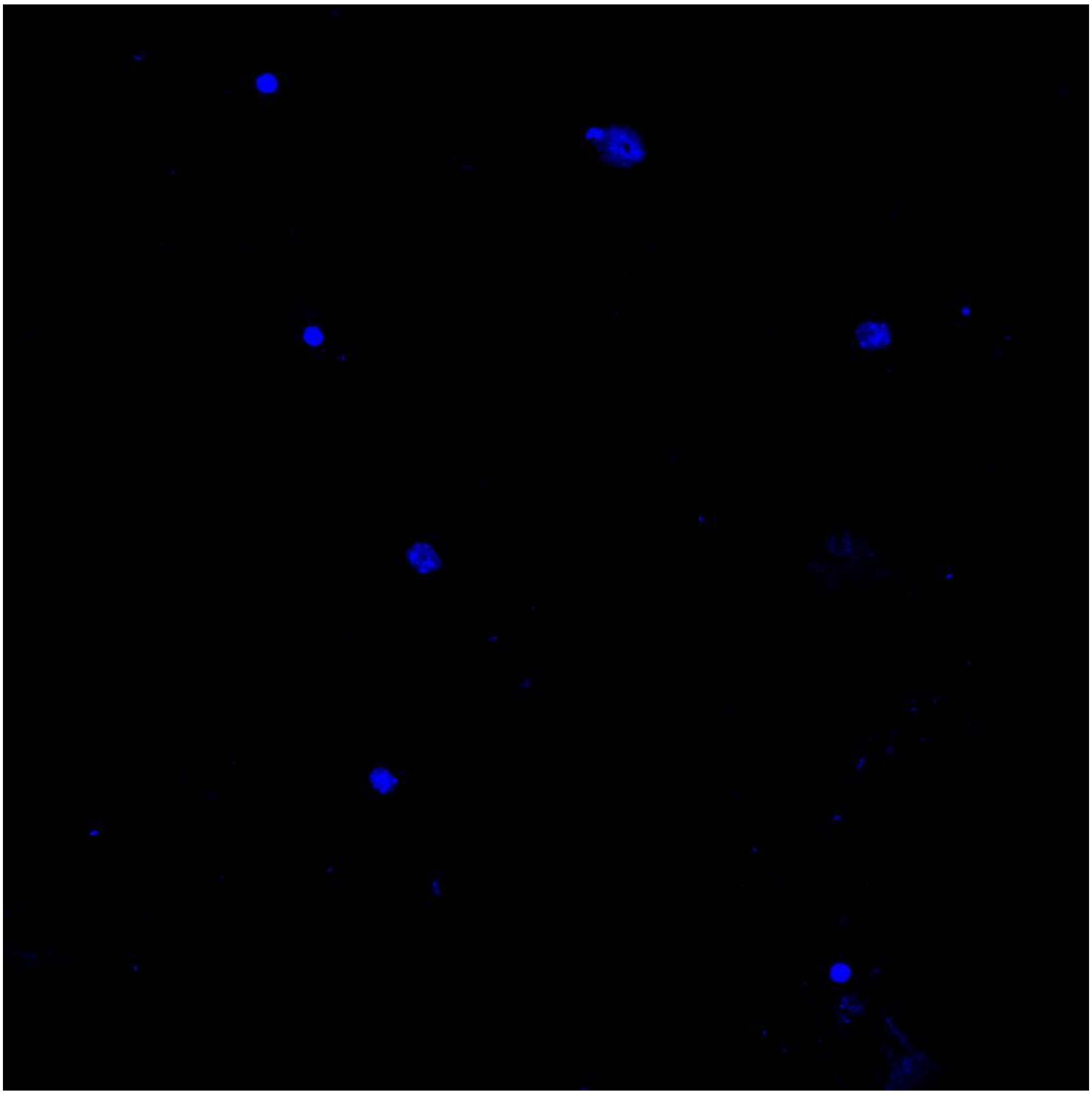

DAPI

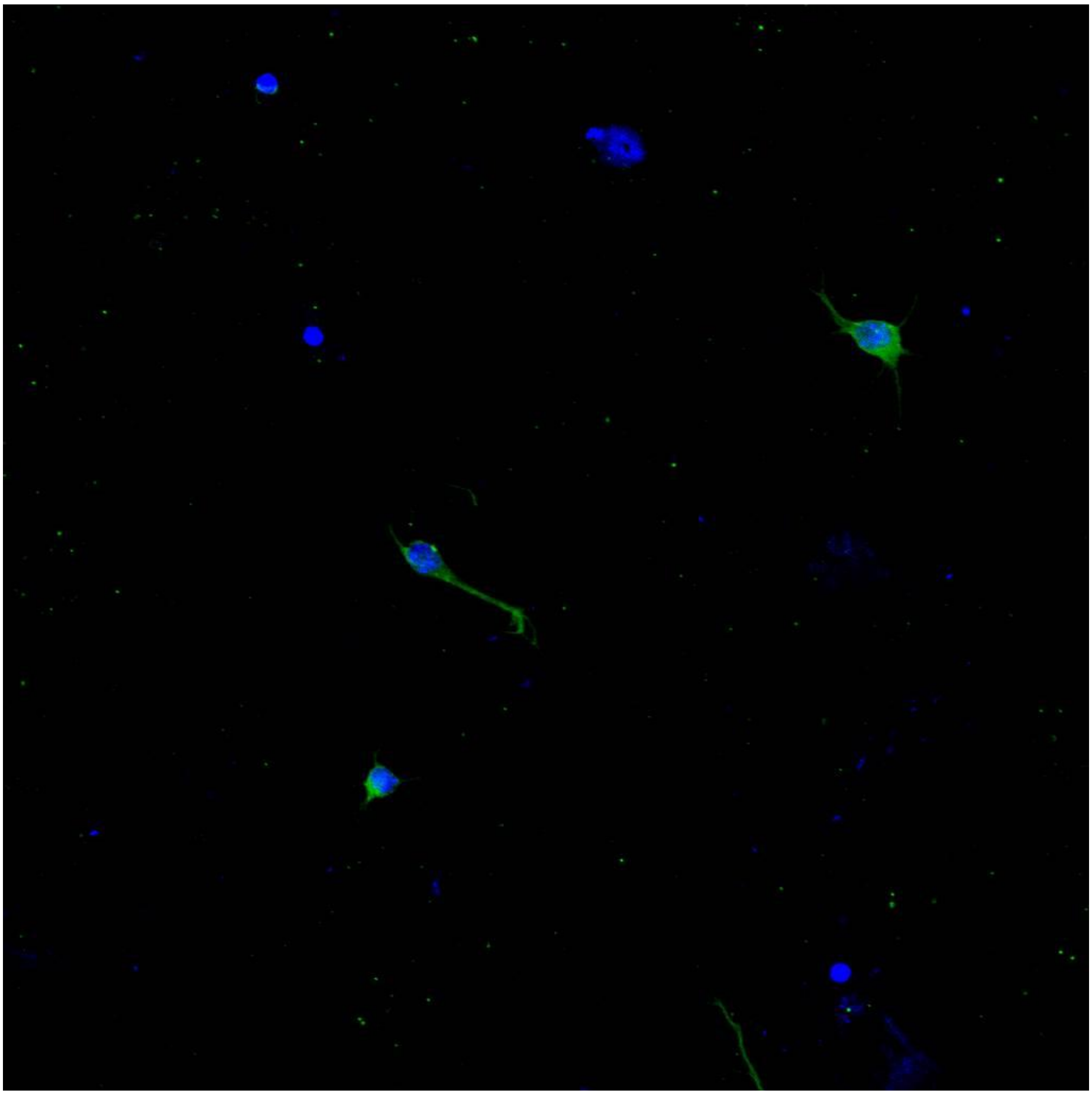

Merge

OGD/R group

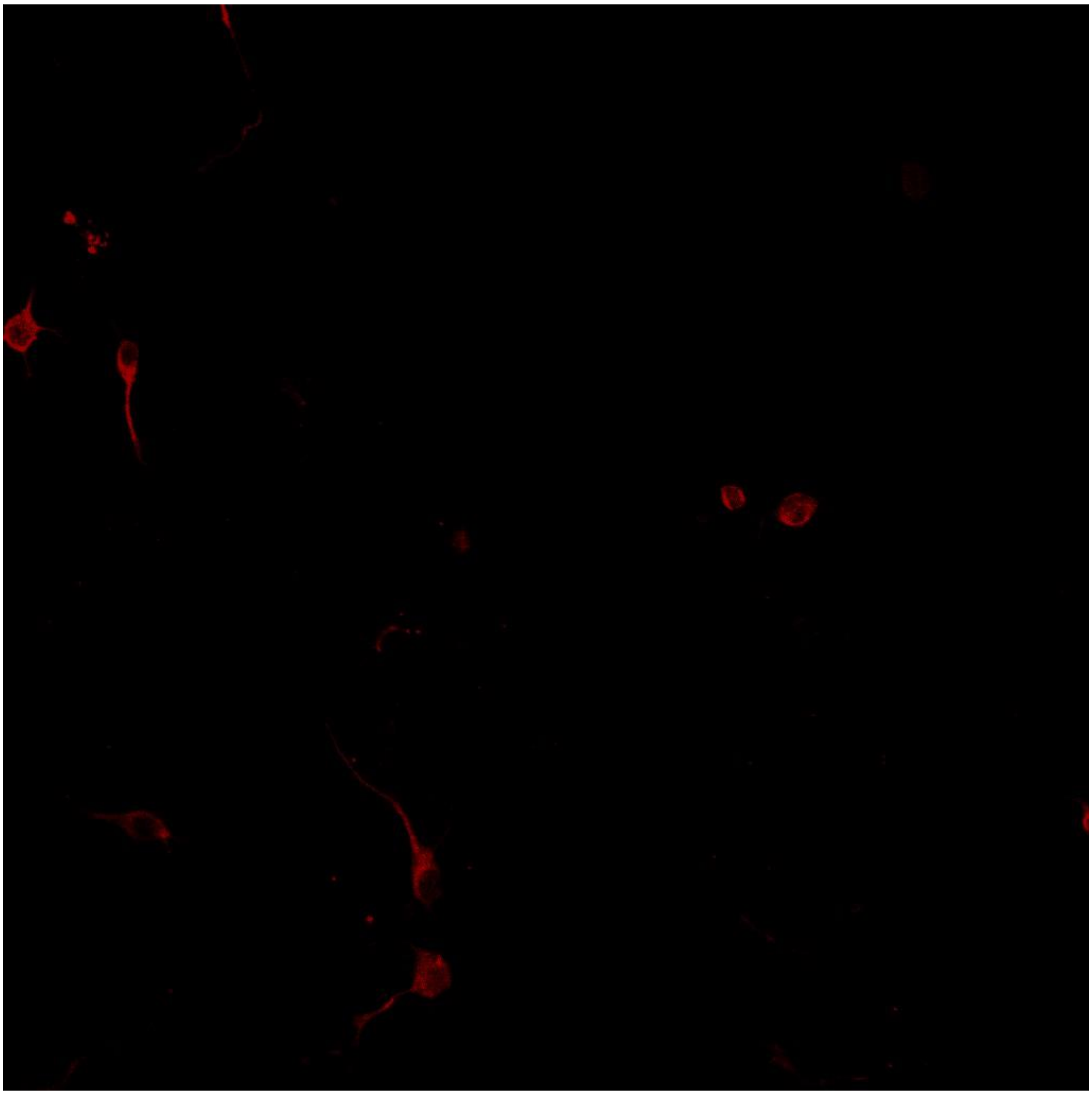

Nrf2

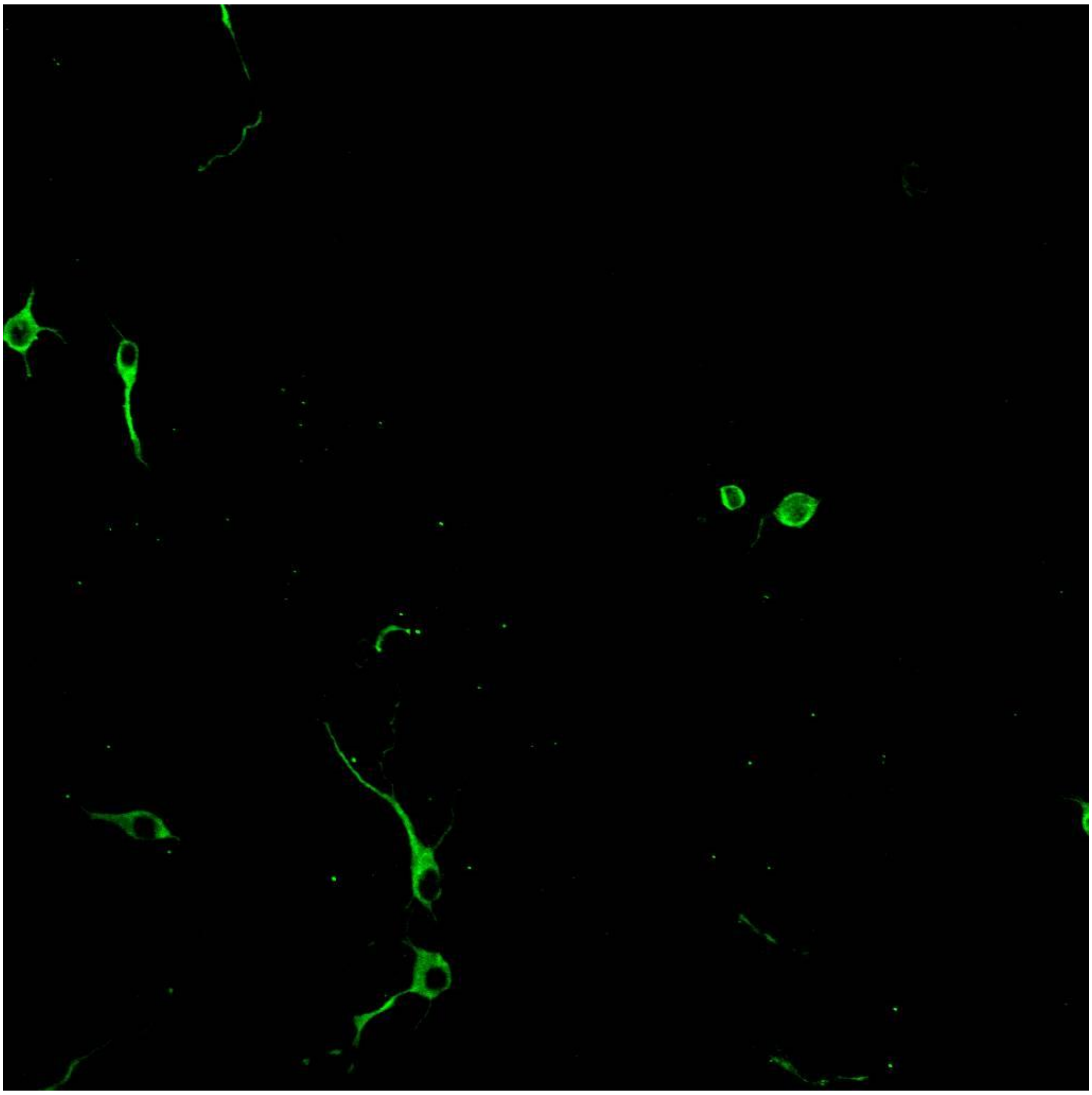
MAP-2

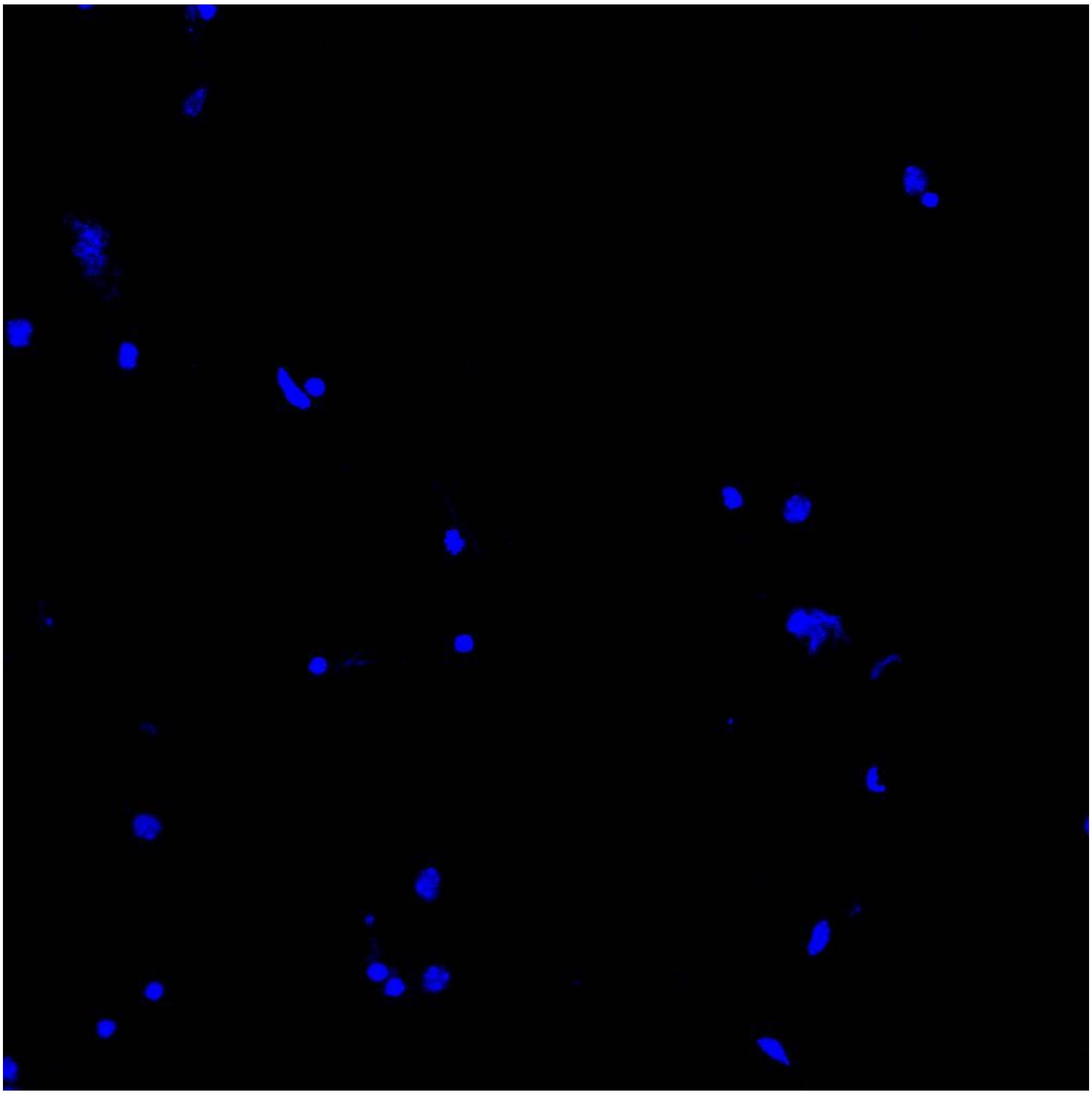
DAPI

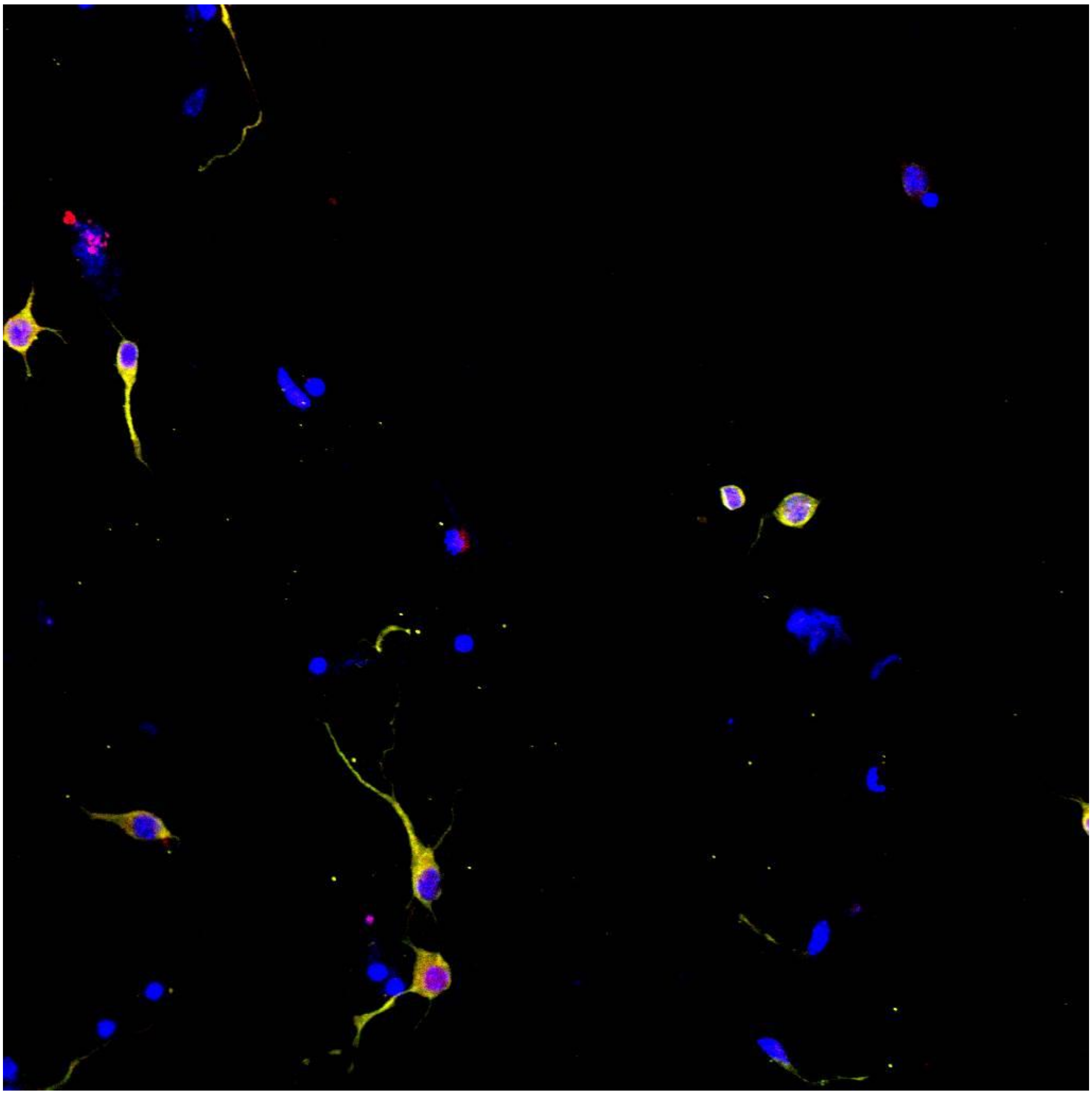

Merge

OGD/R+AST group

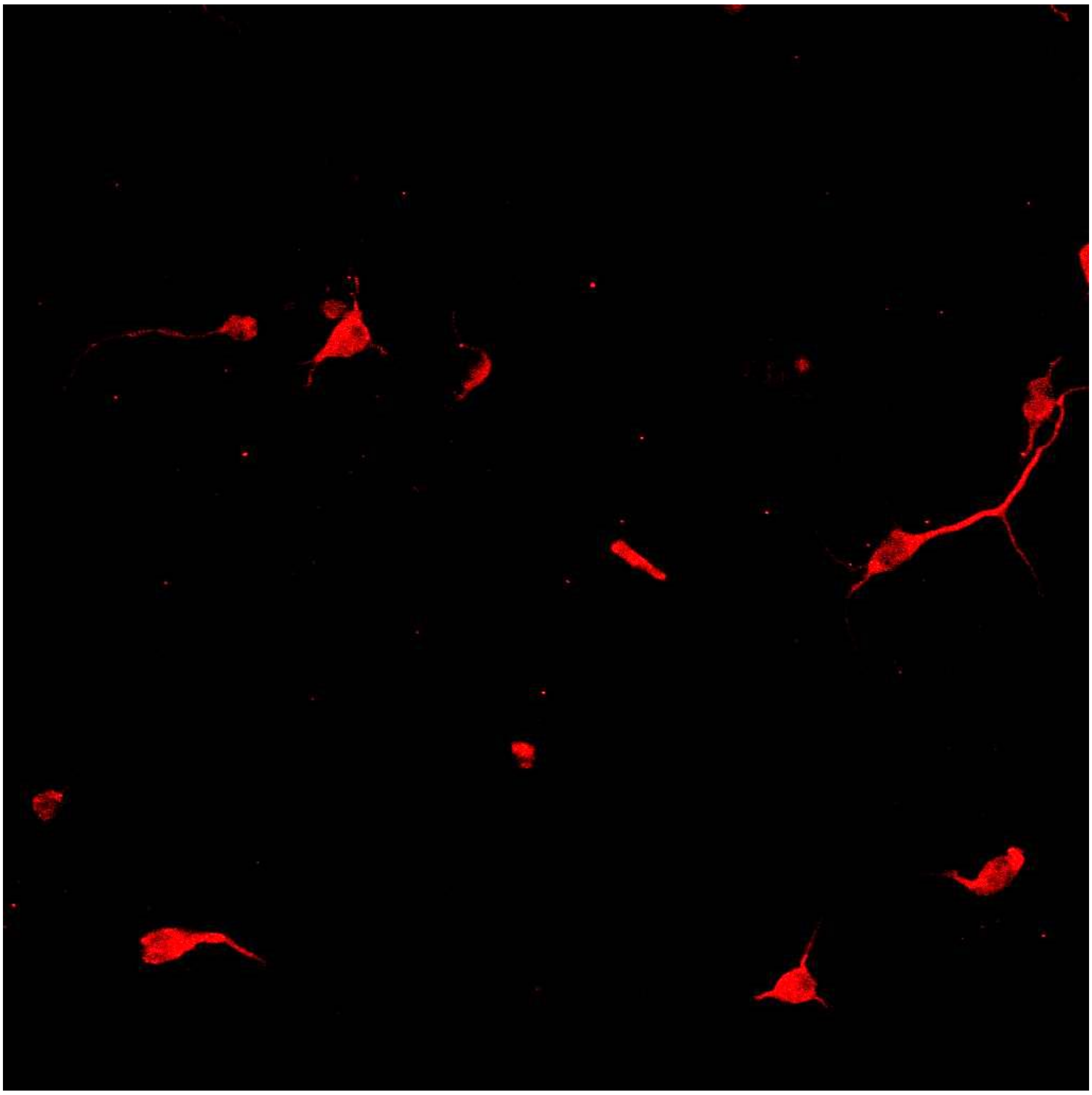

Nrf2

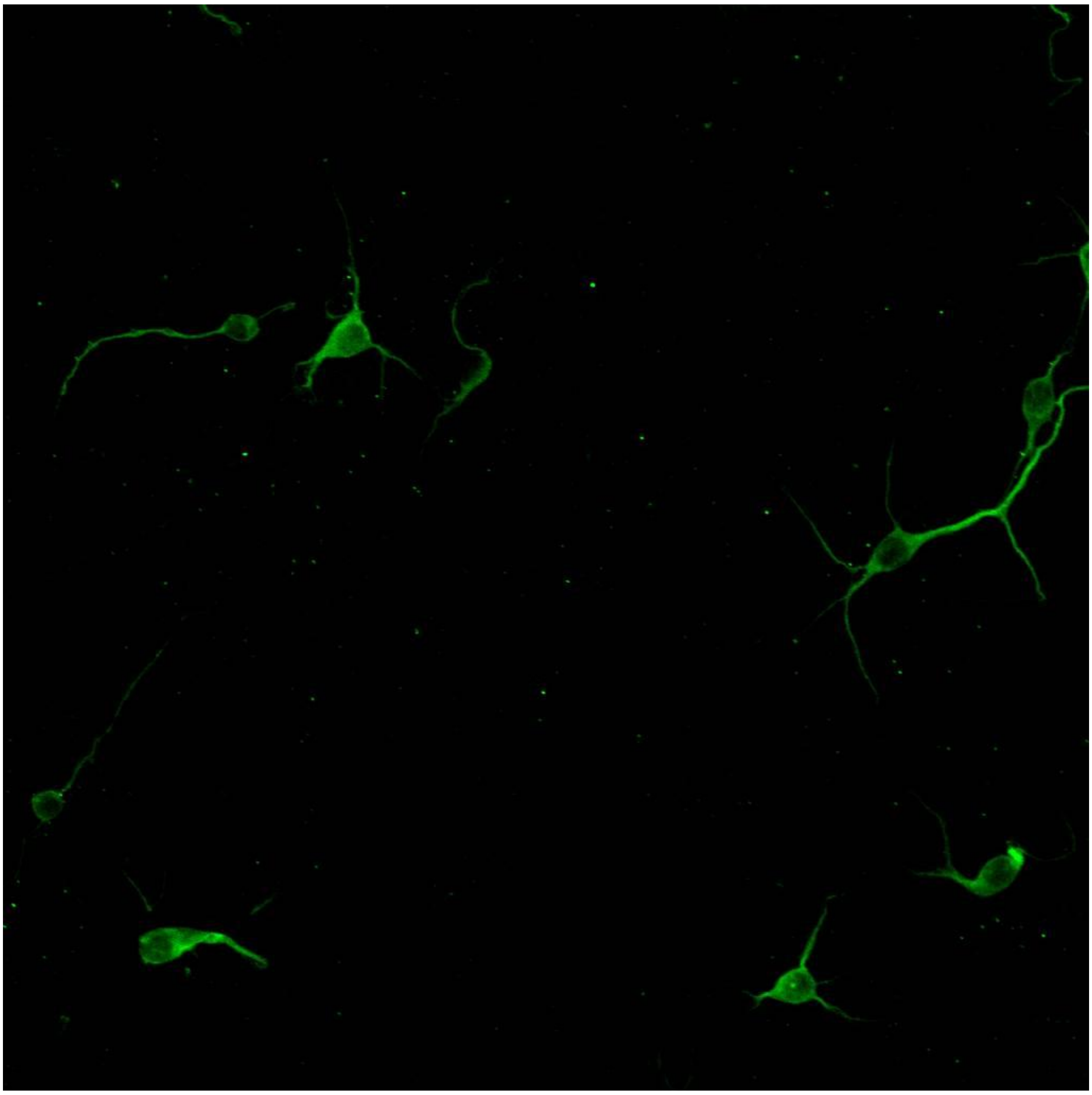

MAP-2

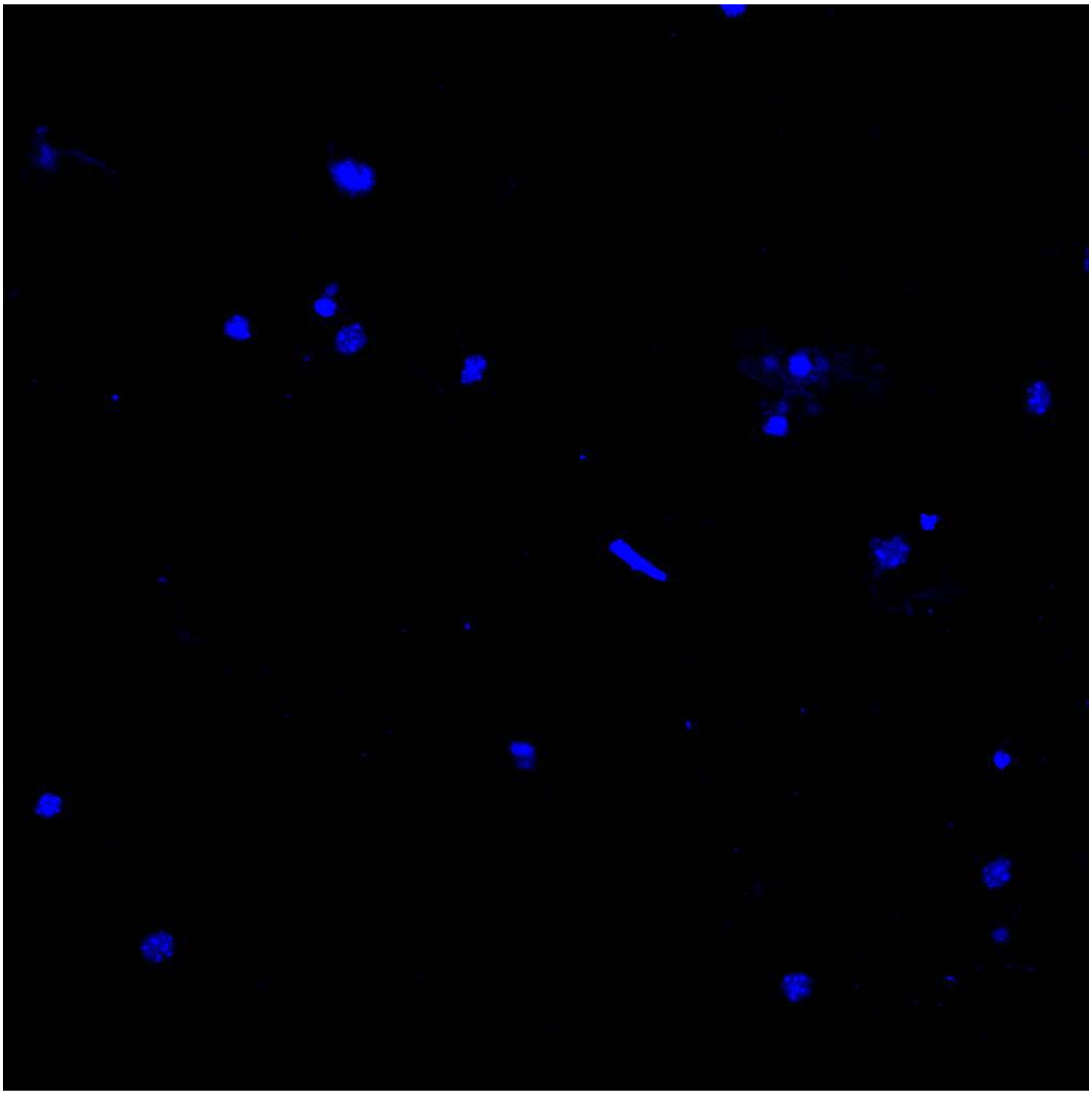

DAPI

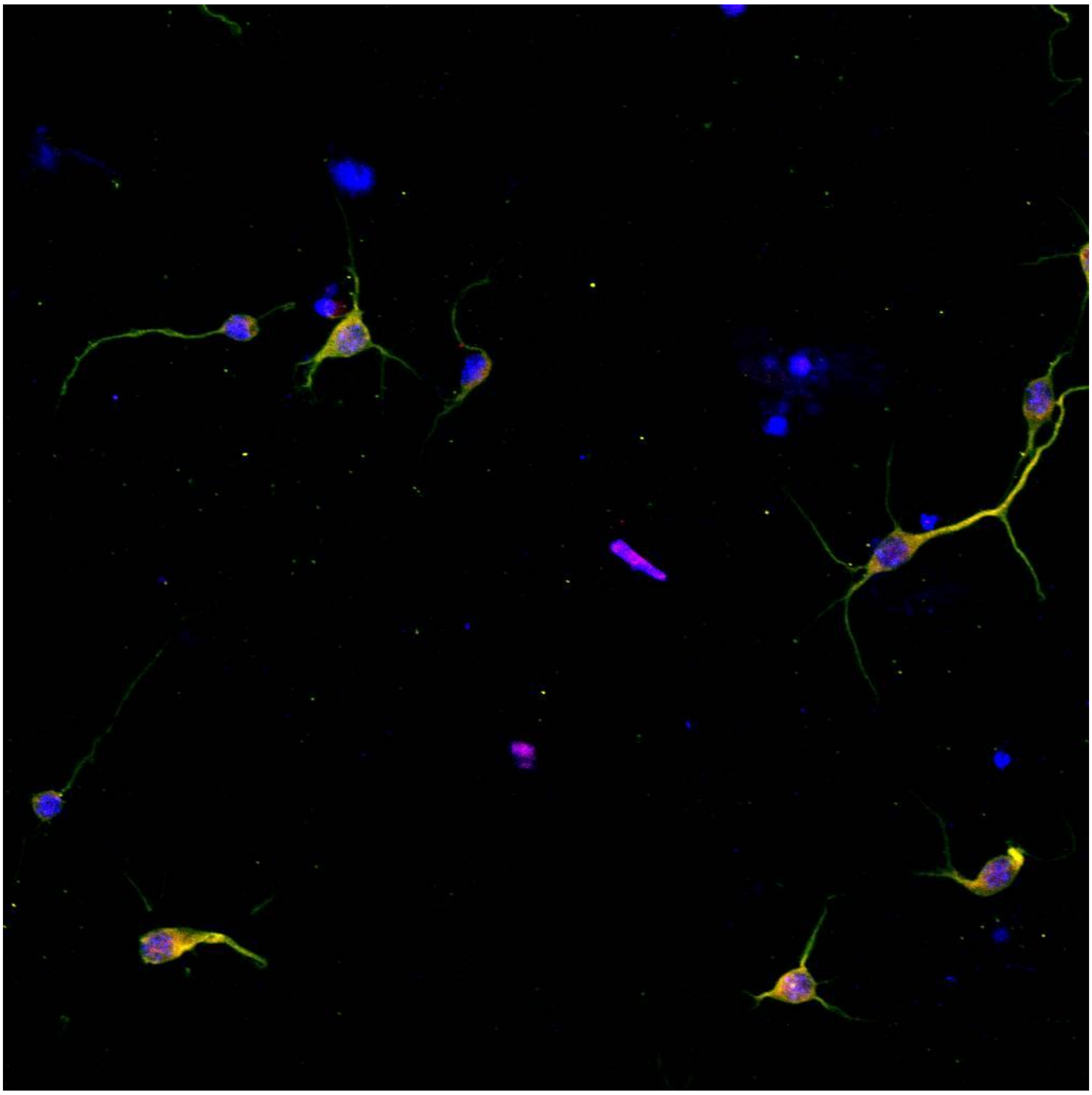

Merge
