# Supplementary material for: Astilbin exerts a neuroprotective effect by upregulating the signaling of nuclear NF-E2-related factor 2 in vitro
Source: Heliyon. 2024 Sep 3;10(17):e37276. doi: 10.1016/j.heliyon.2024.e37276 (PMC11409207; doi:10.1016/j.heliyon.2024.e37276)

Ctrl group

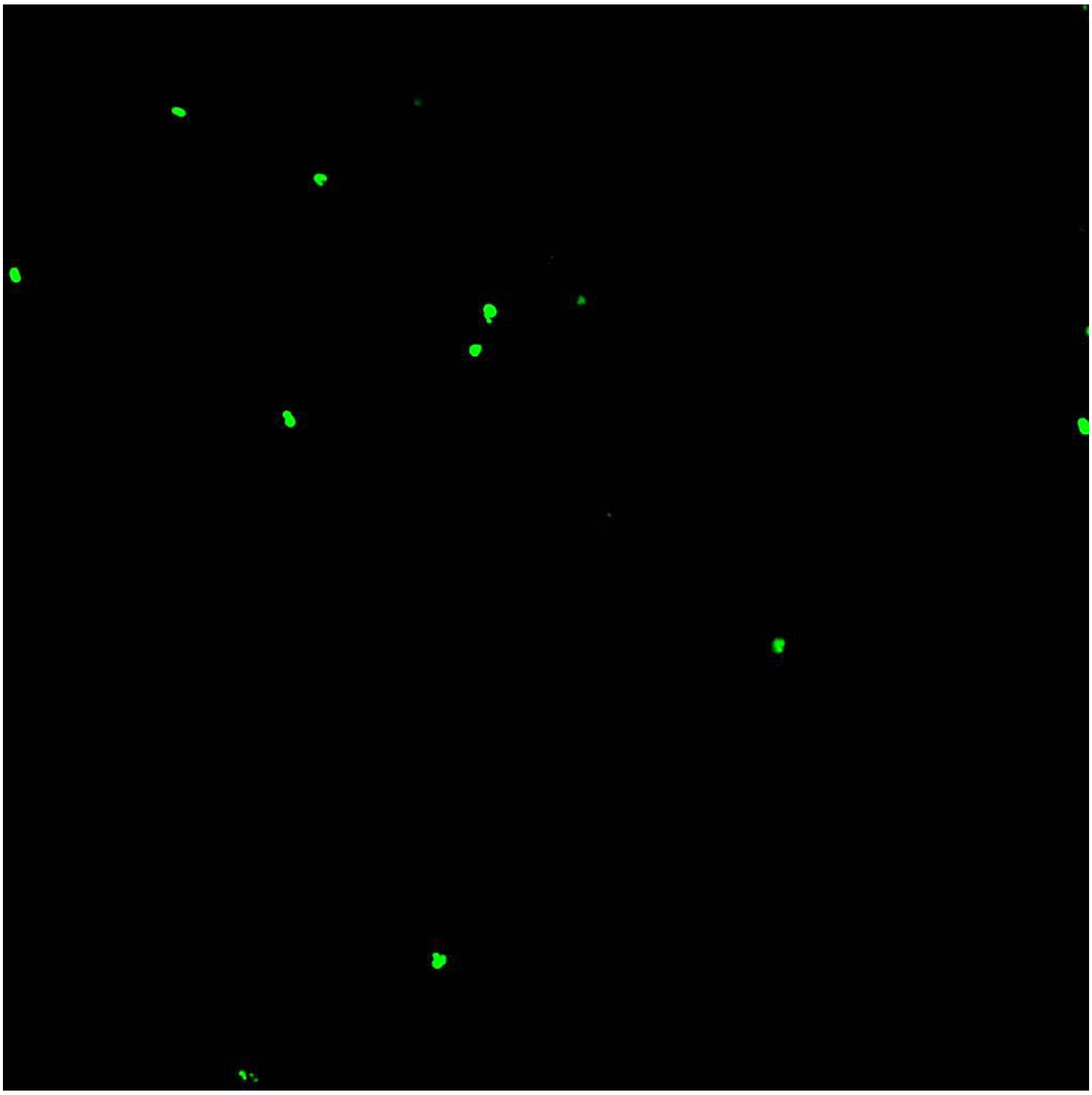

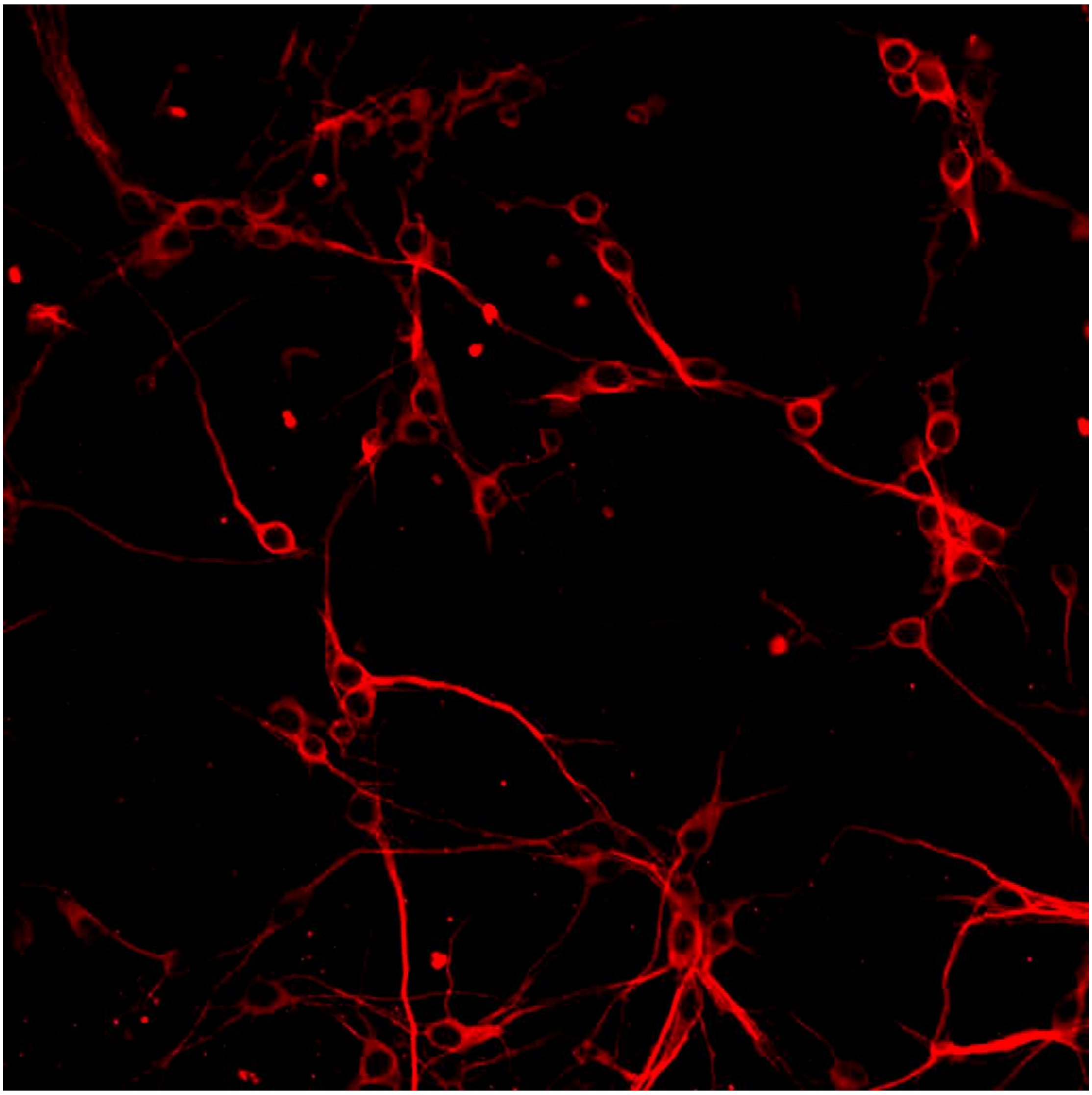

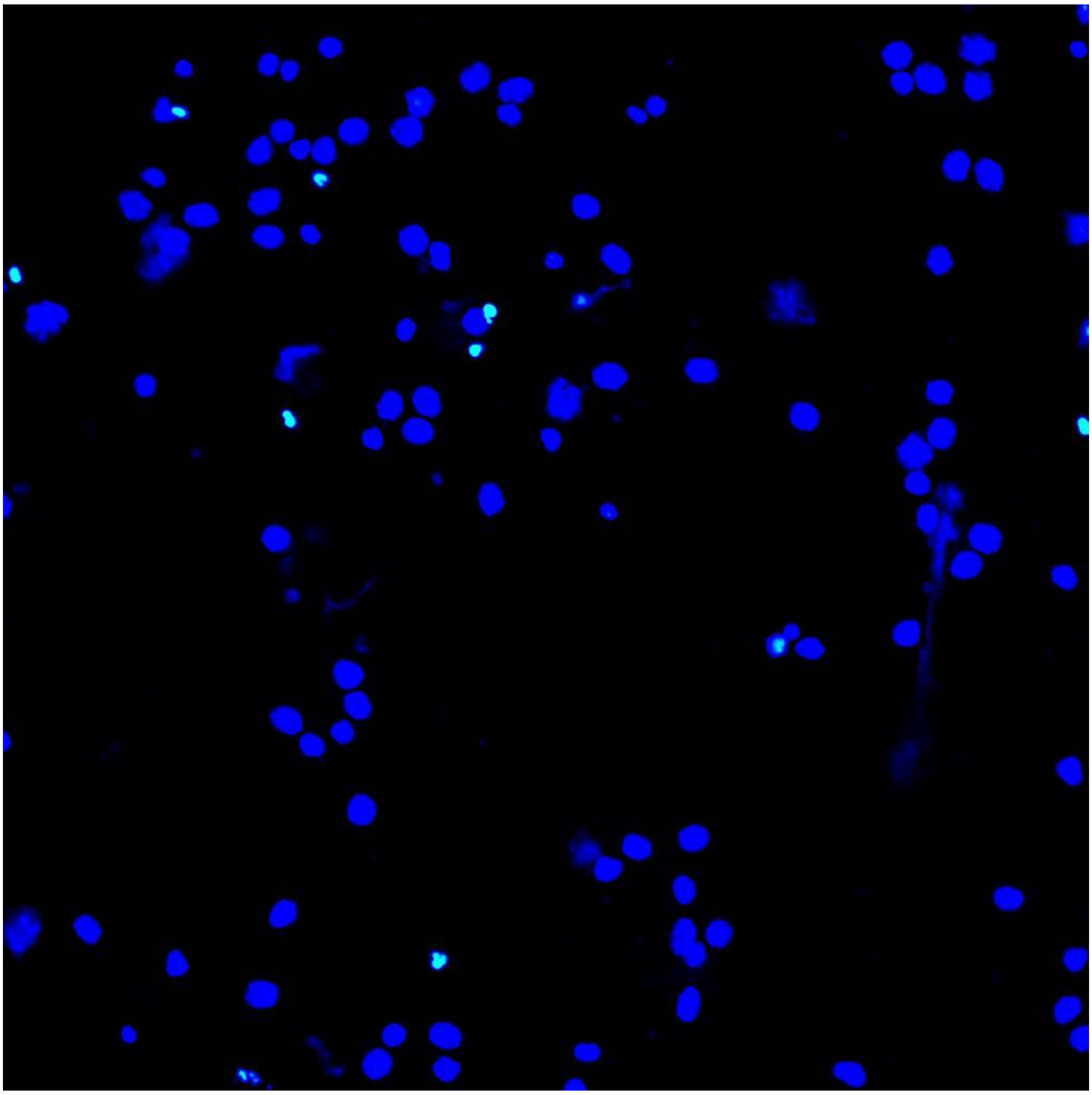

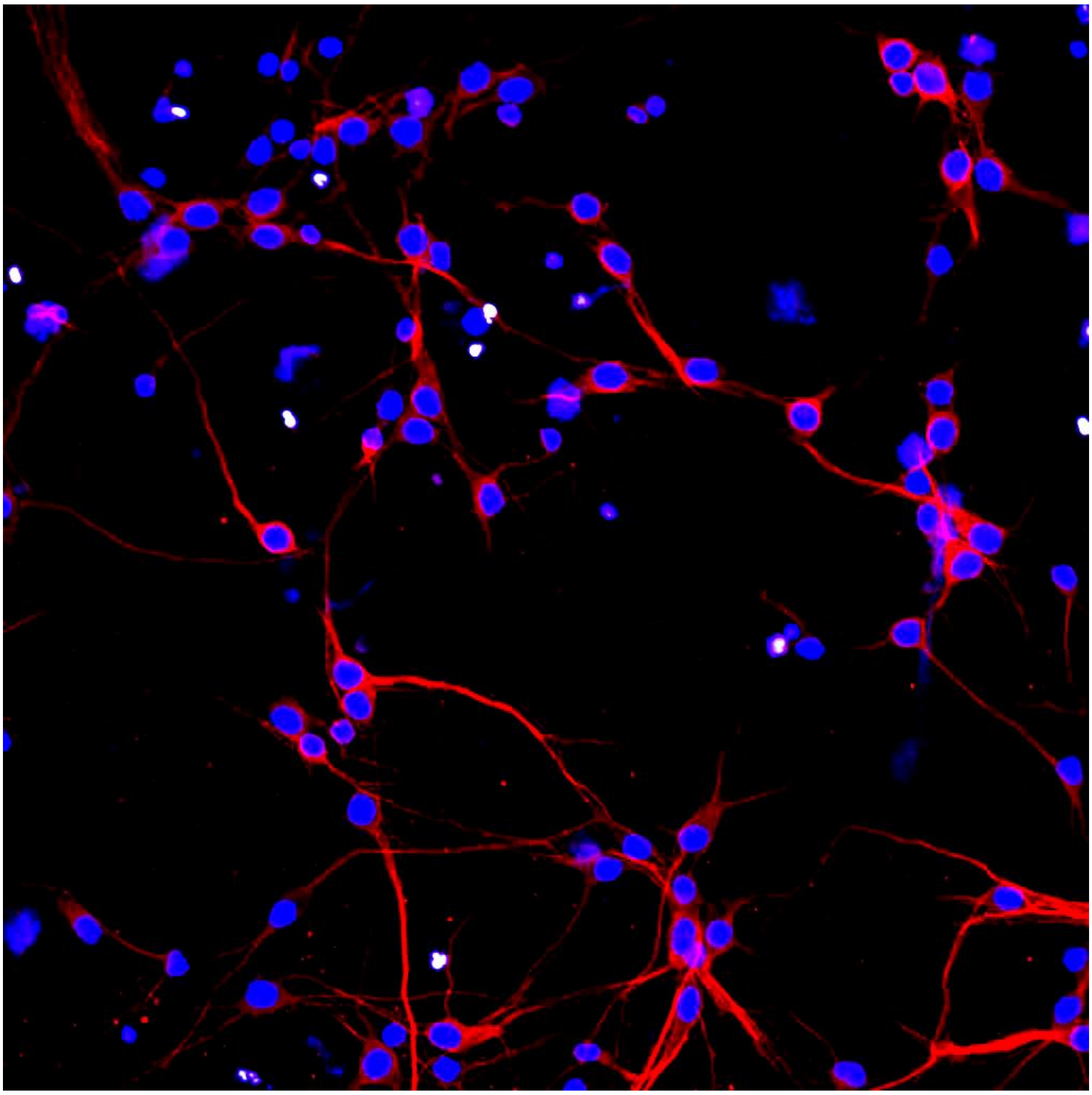

Ctrl+AST group

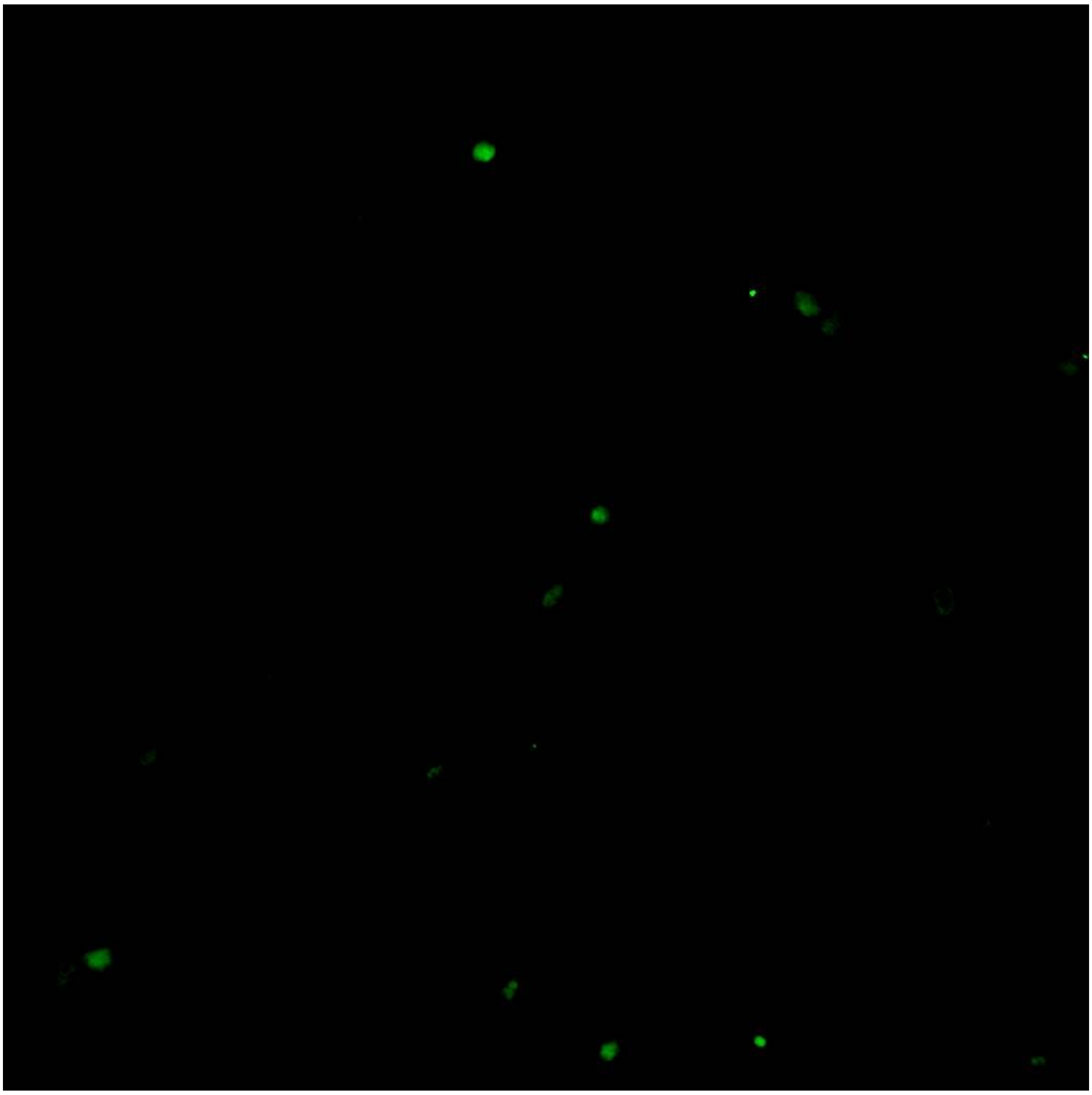

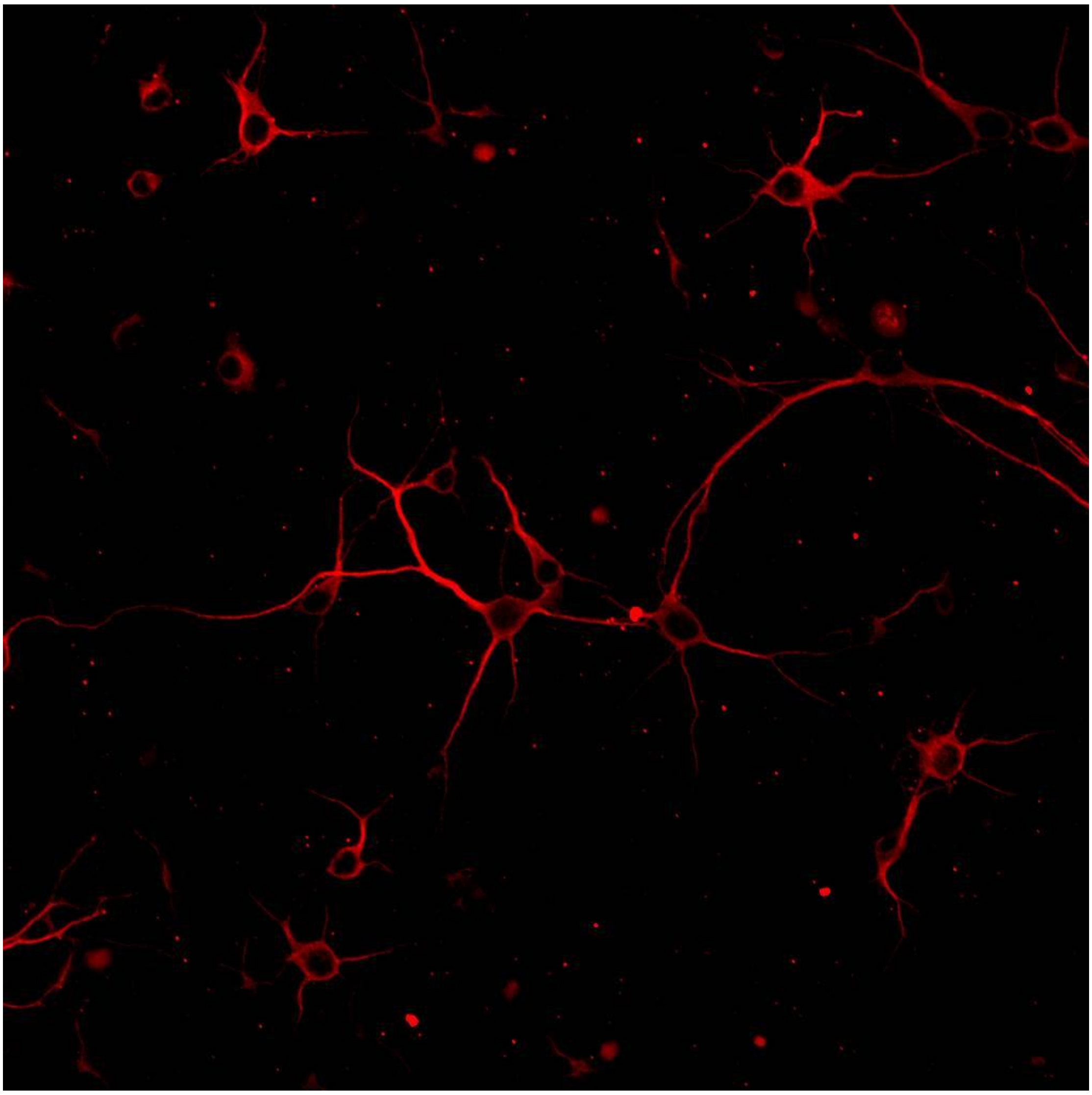

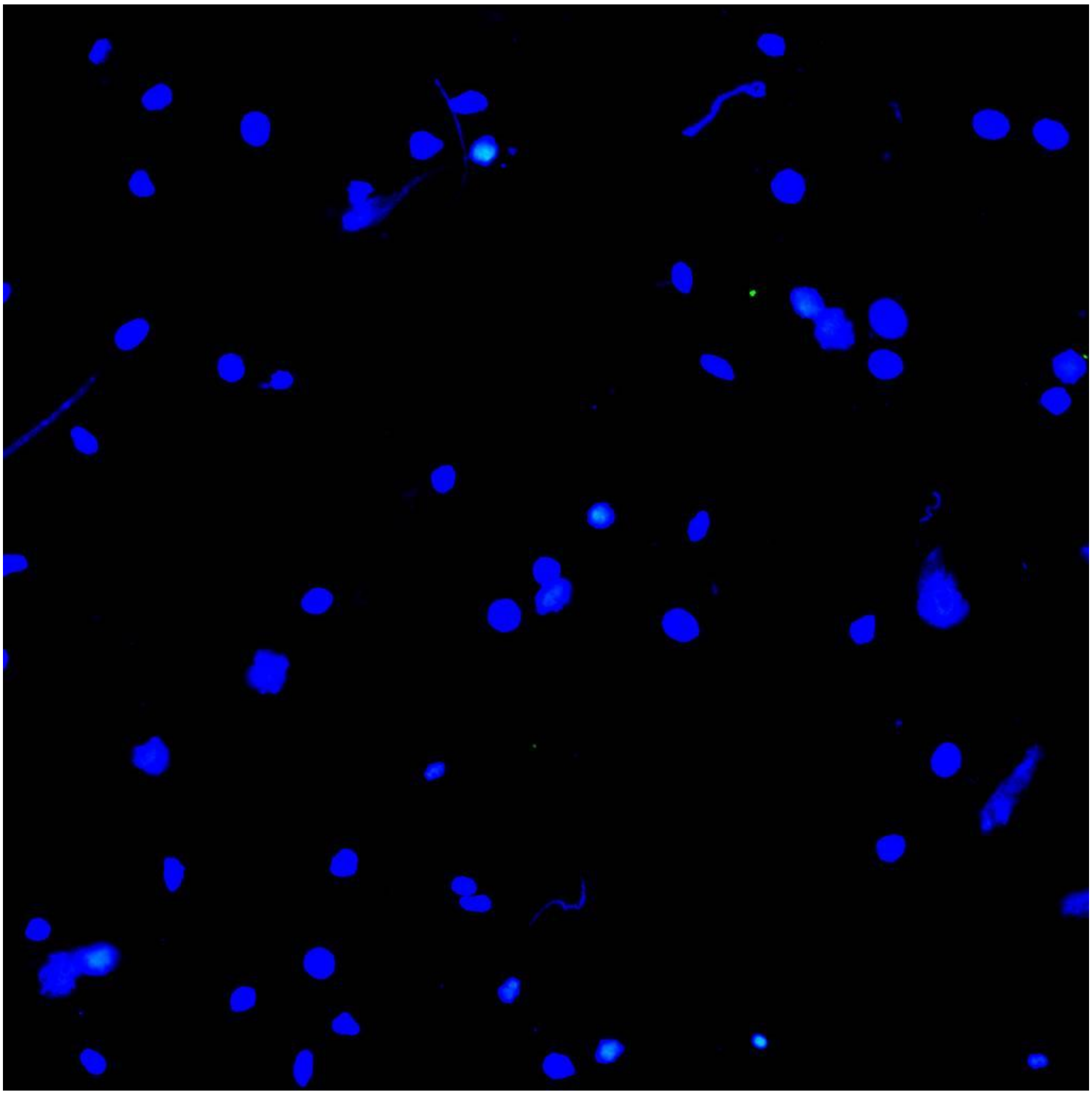

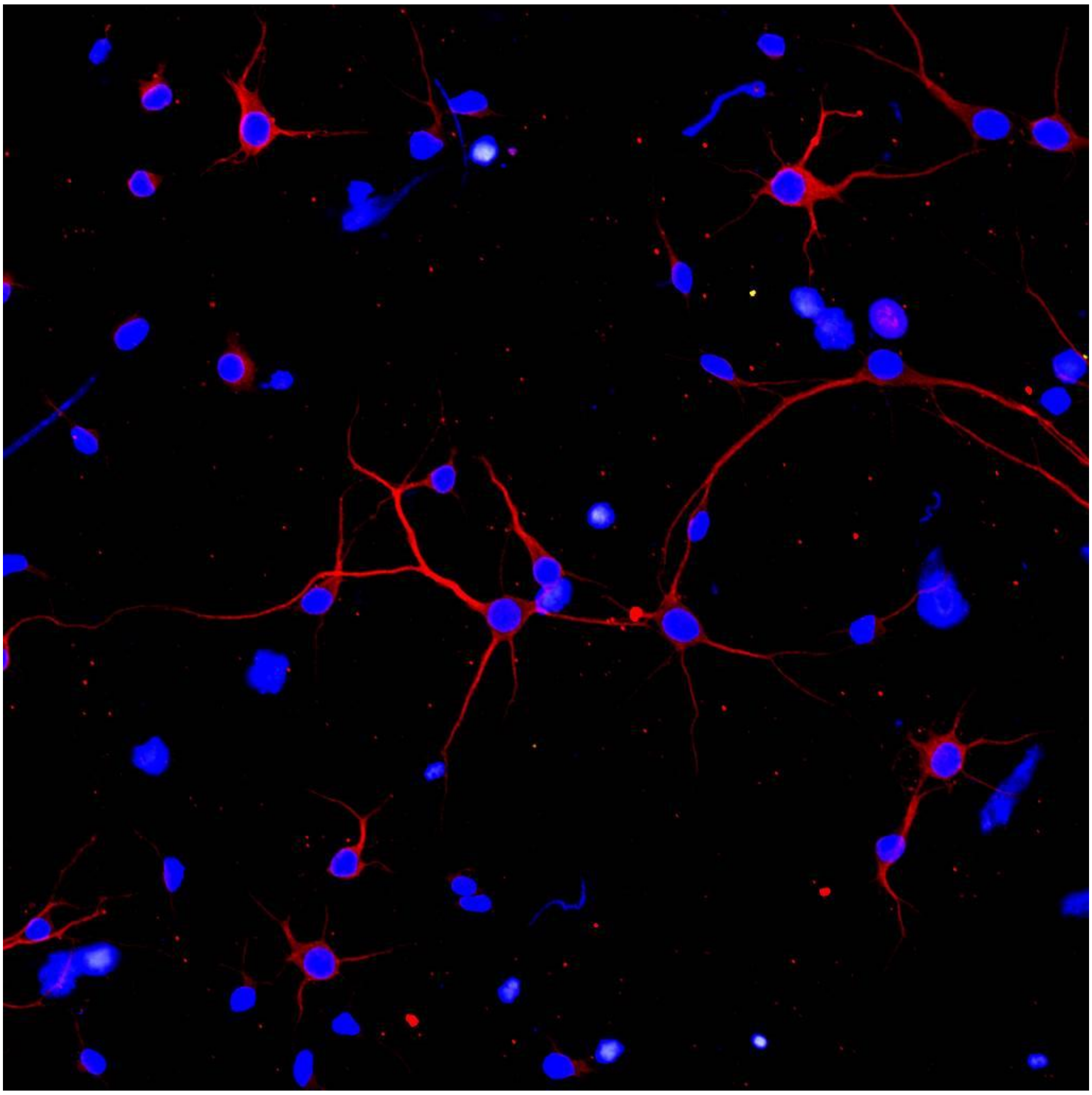

OGD/R group

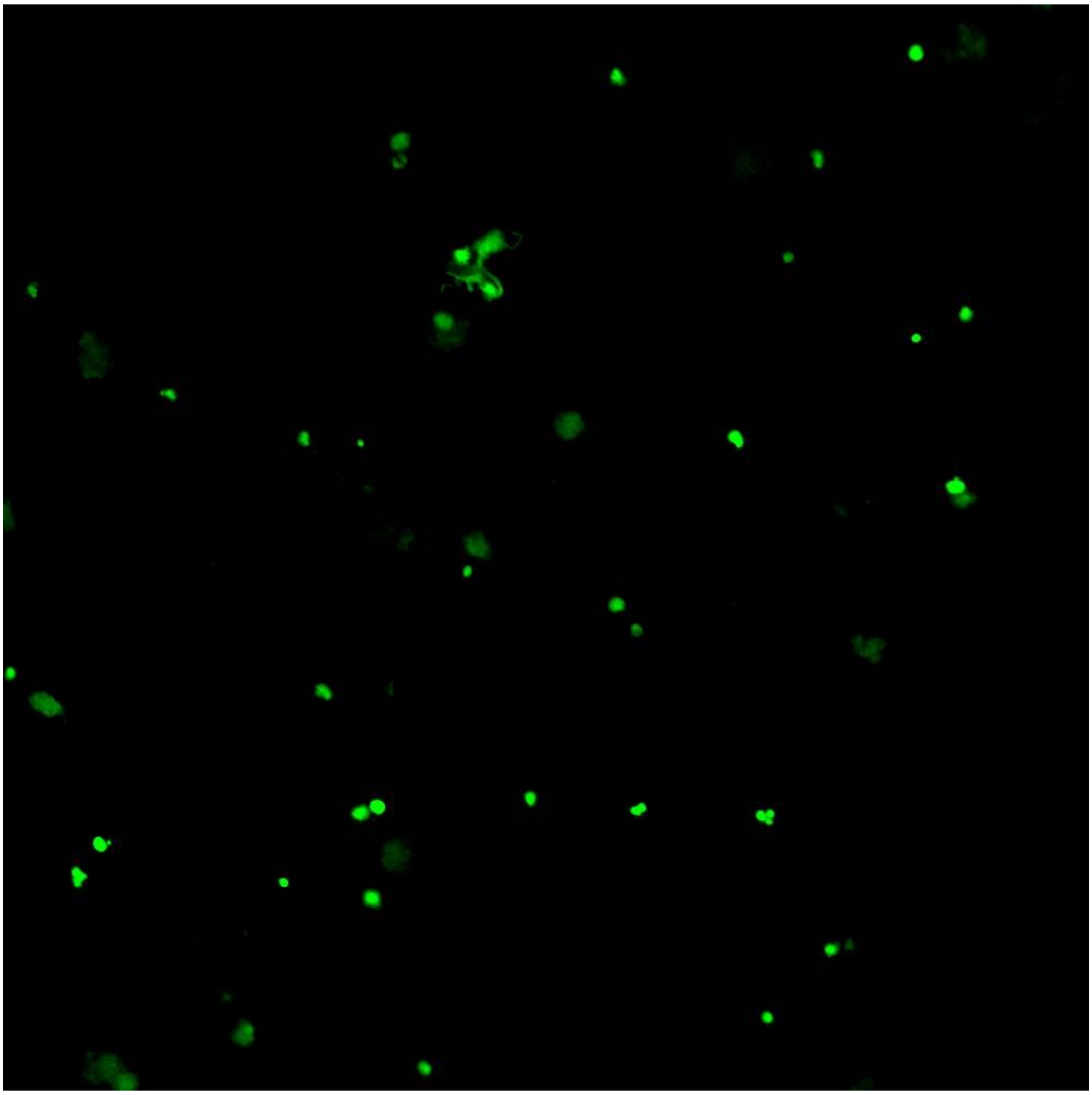

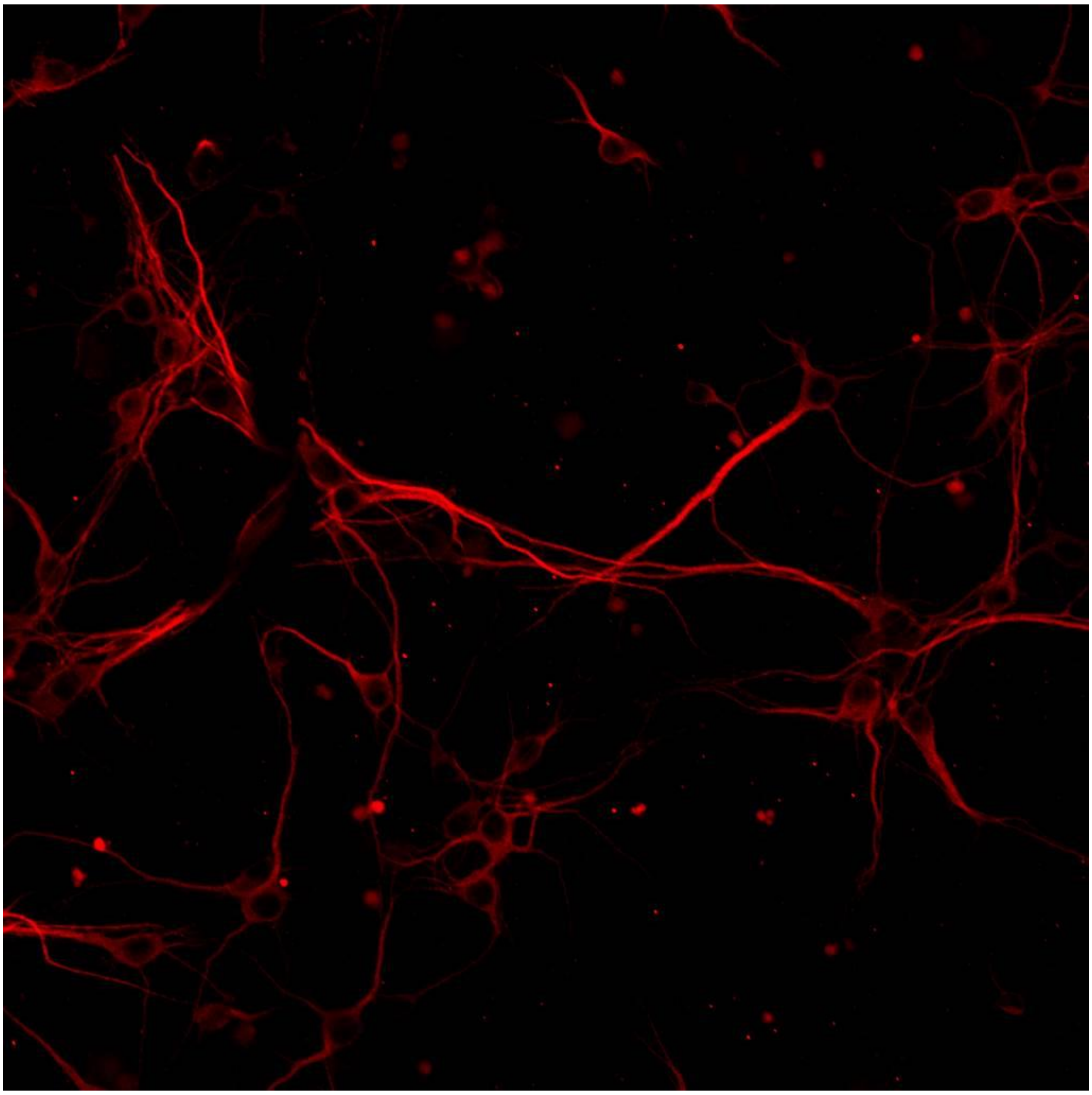

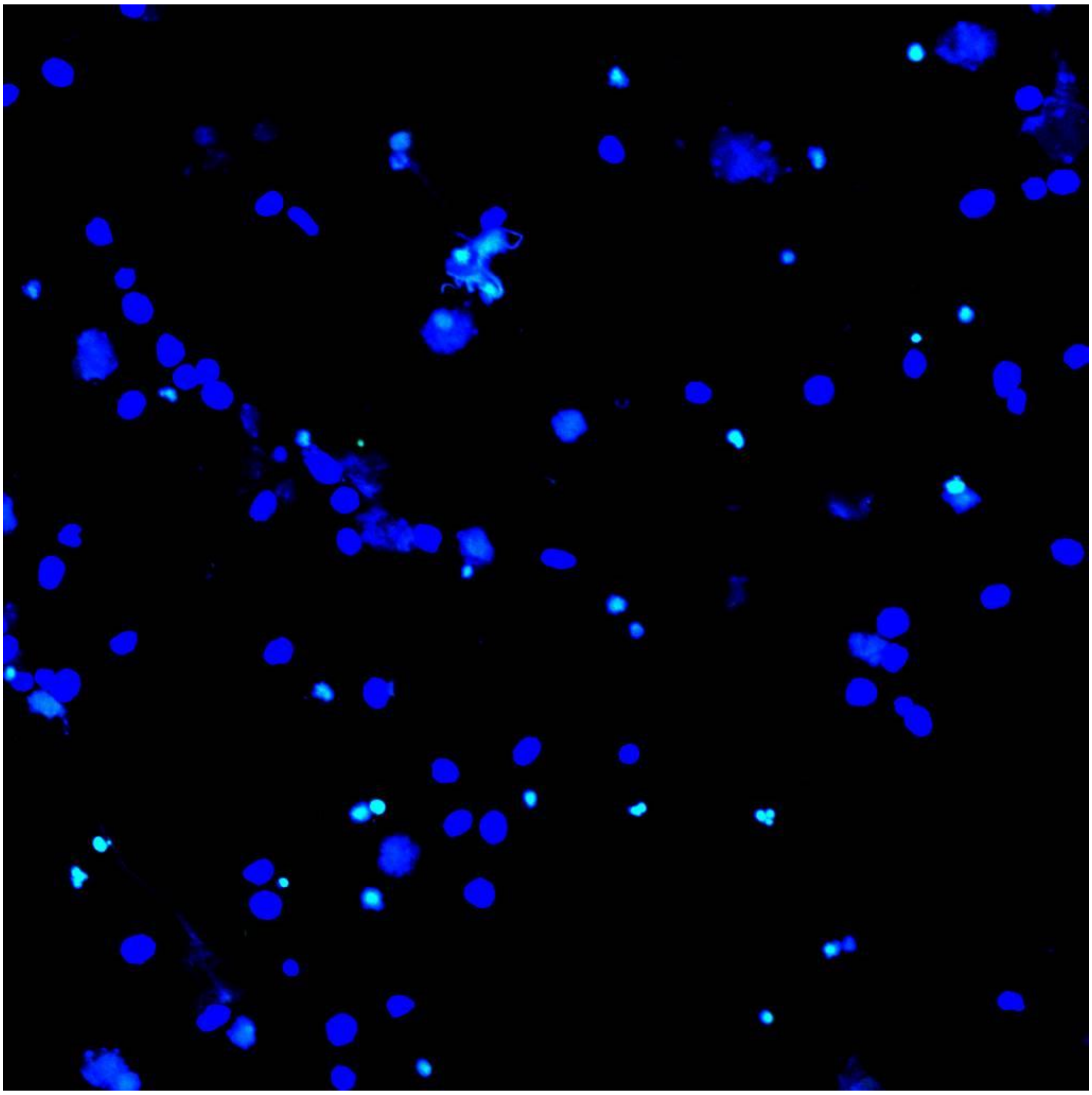

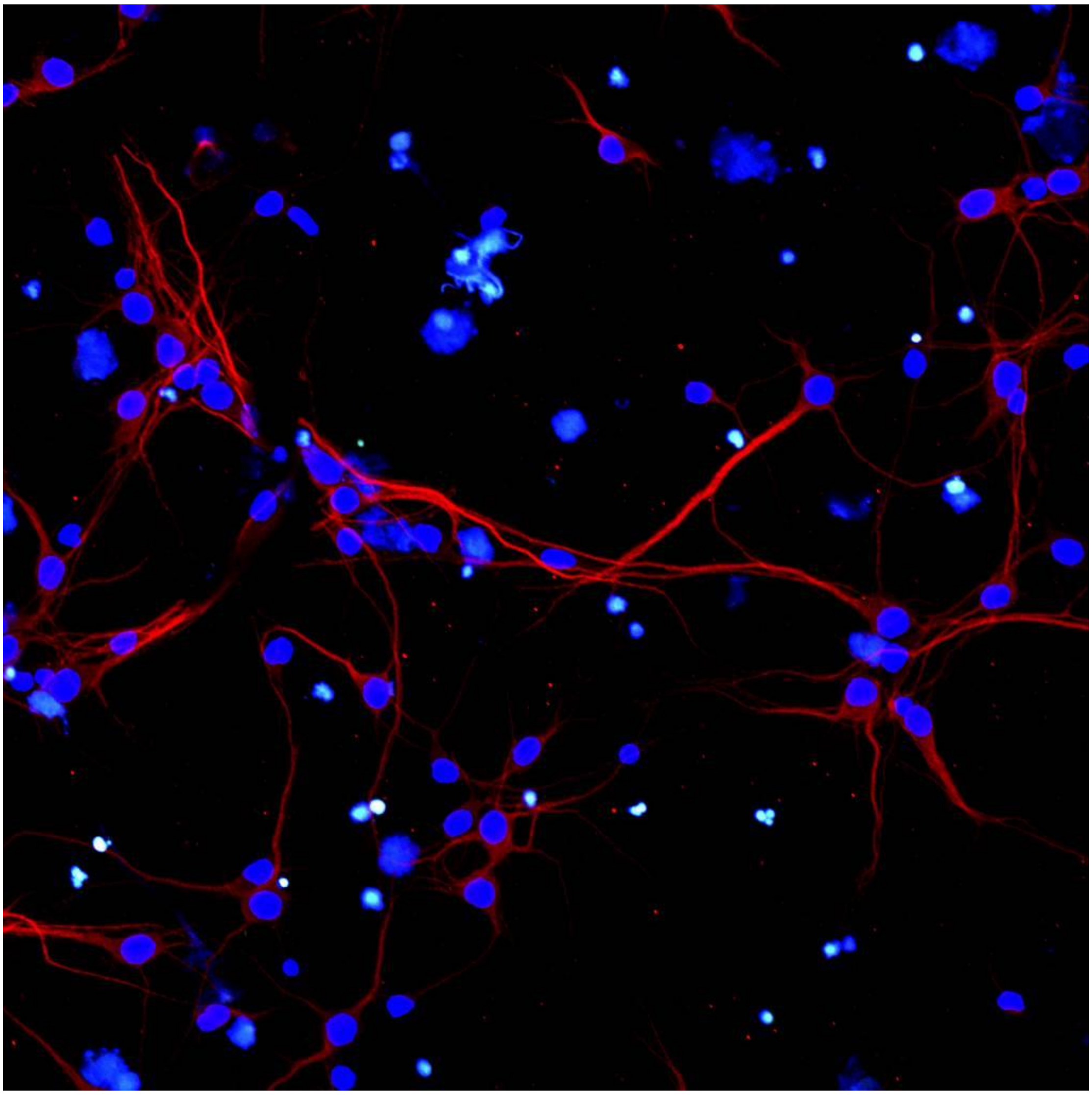

OGD/R+AST group

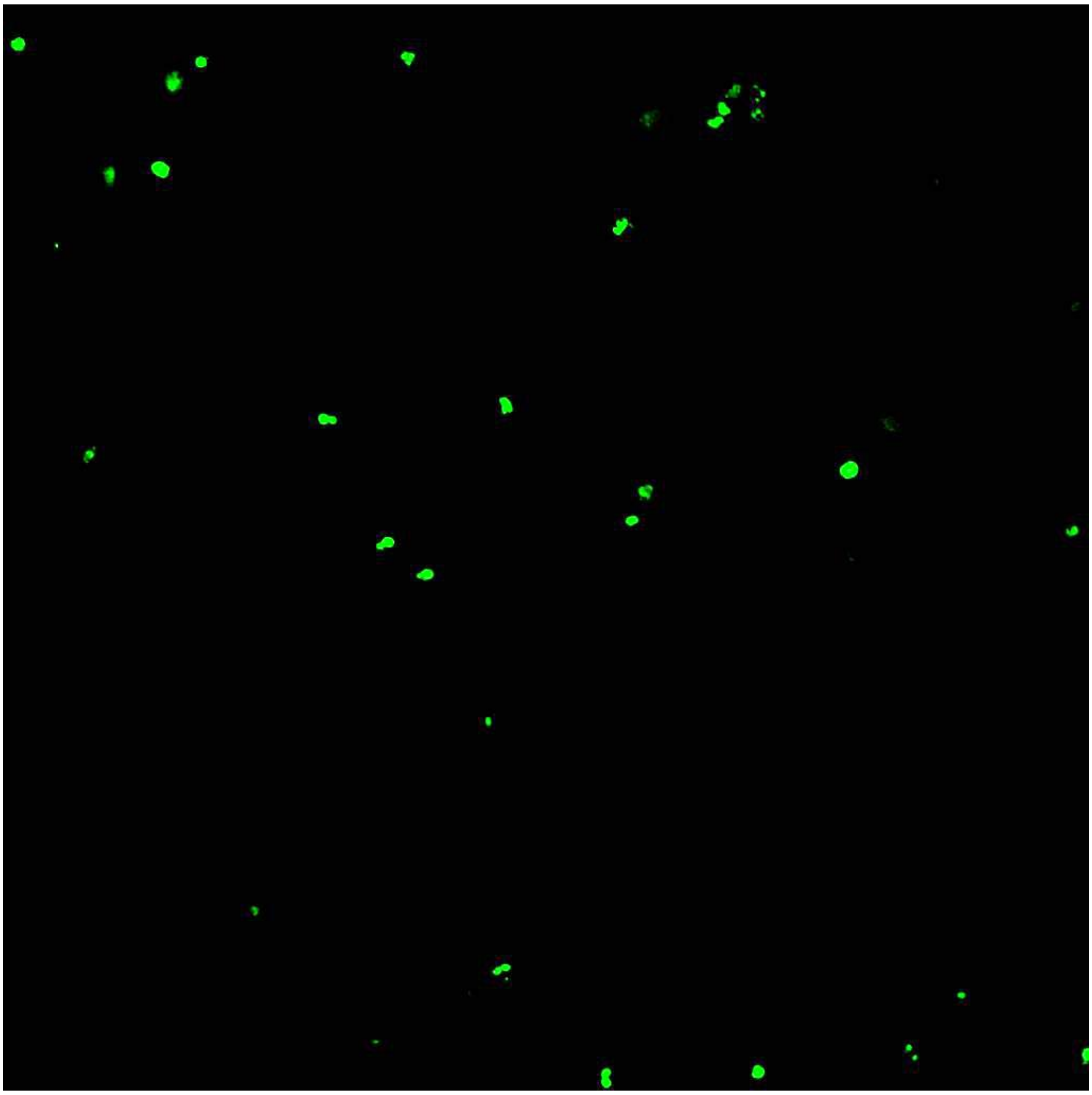

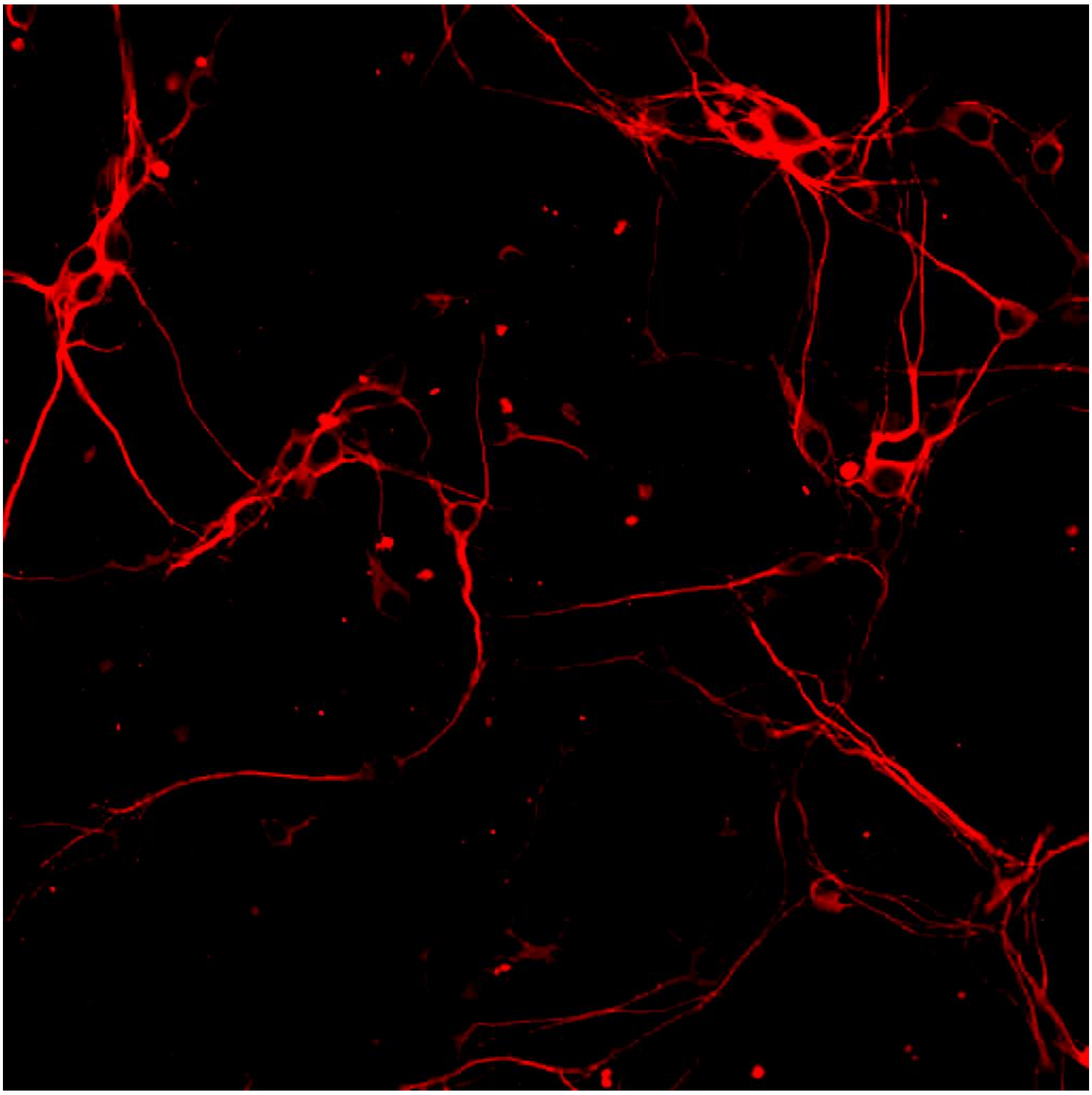

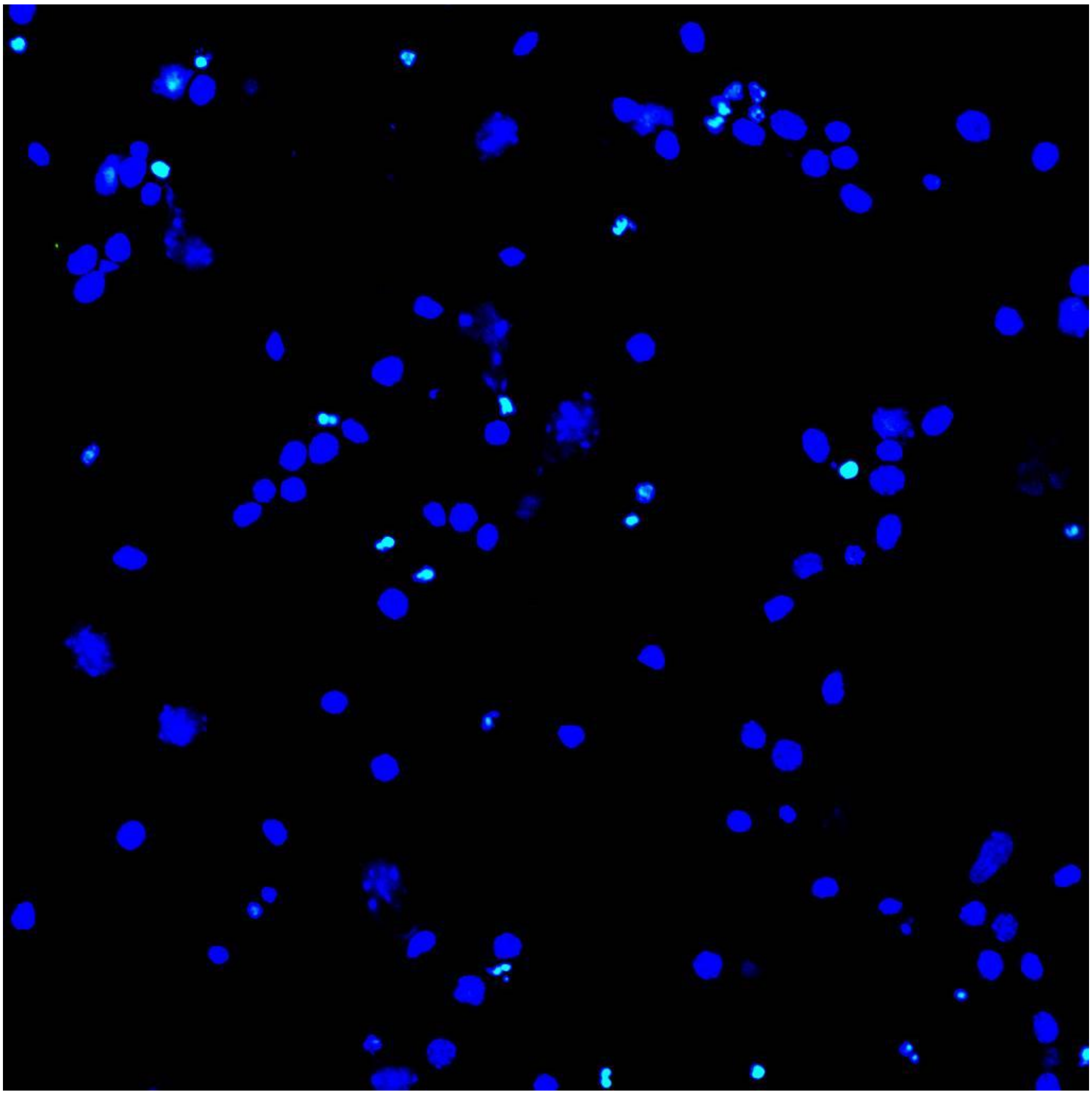

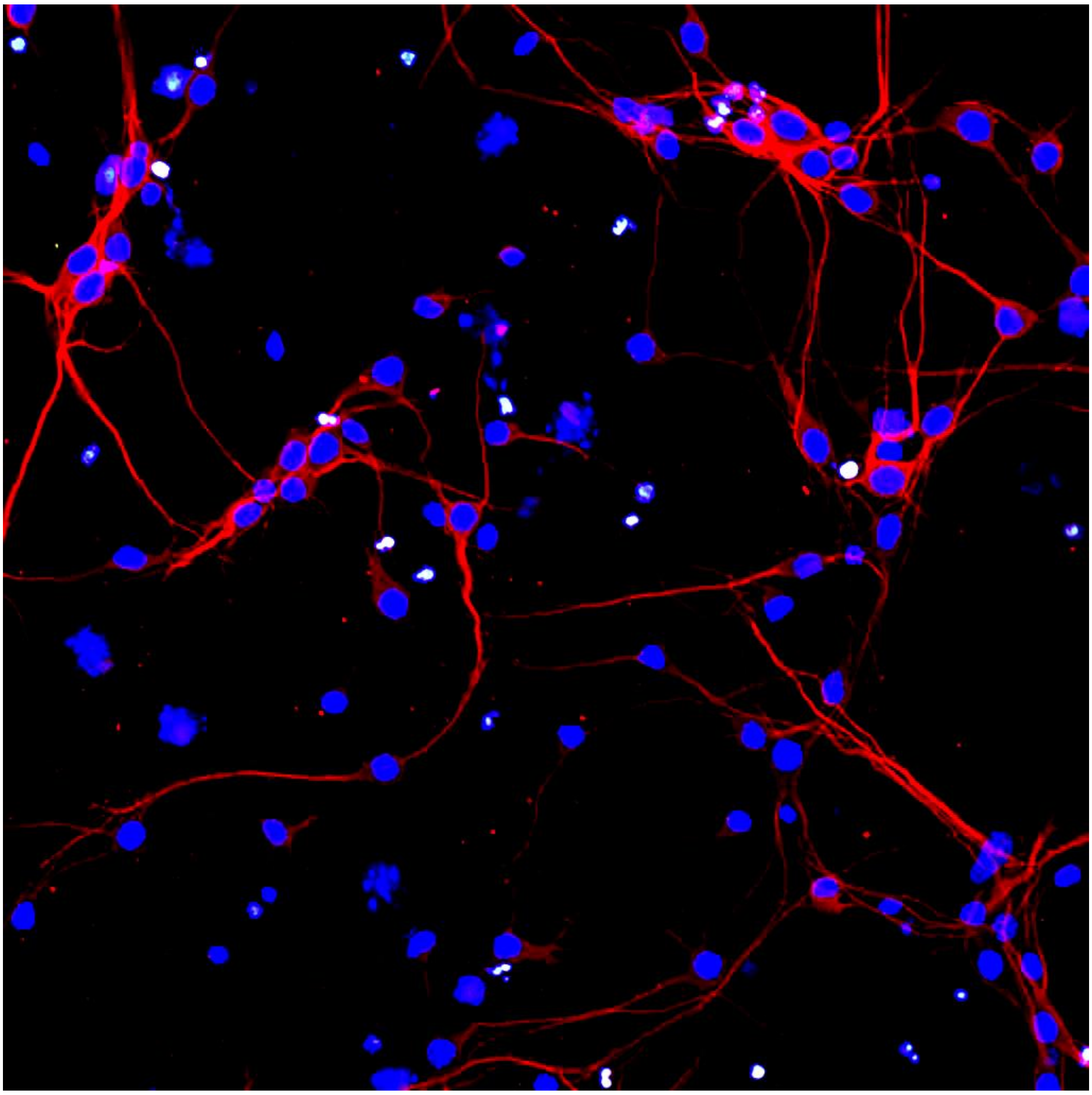

OGD/R+AST group

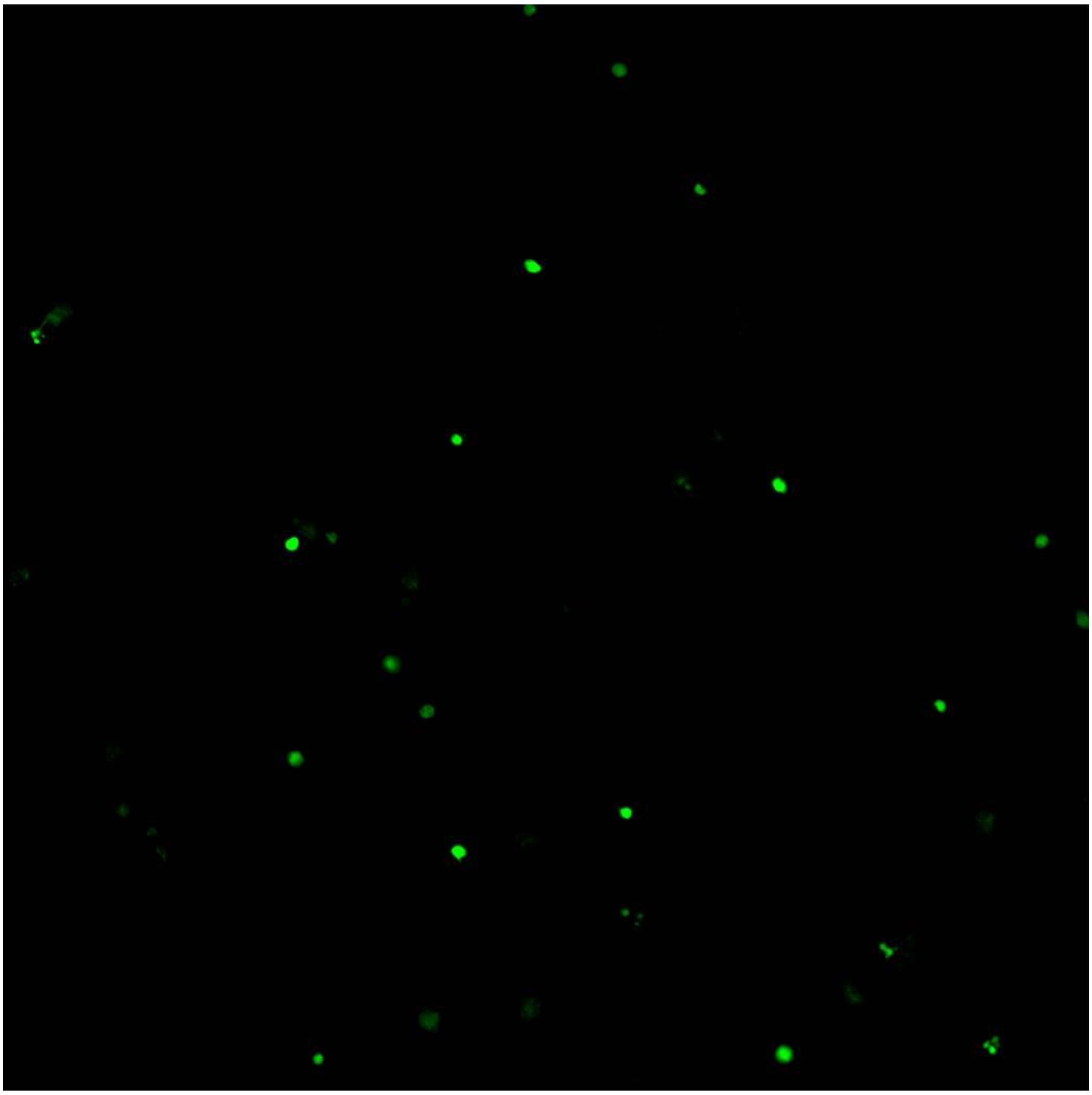

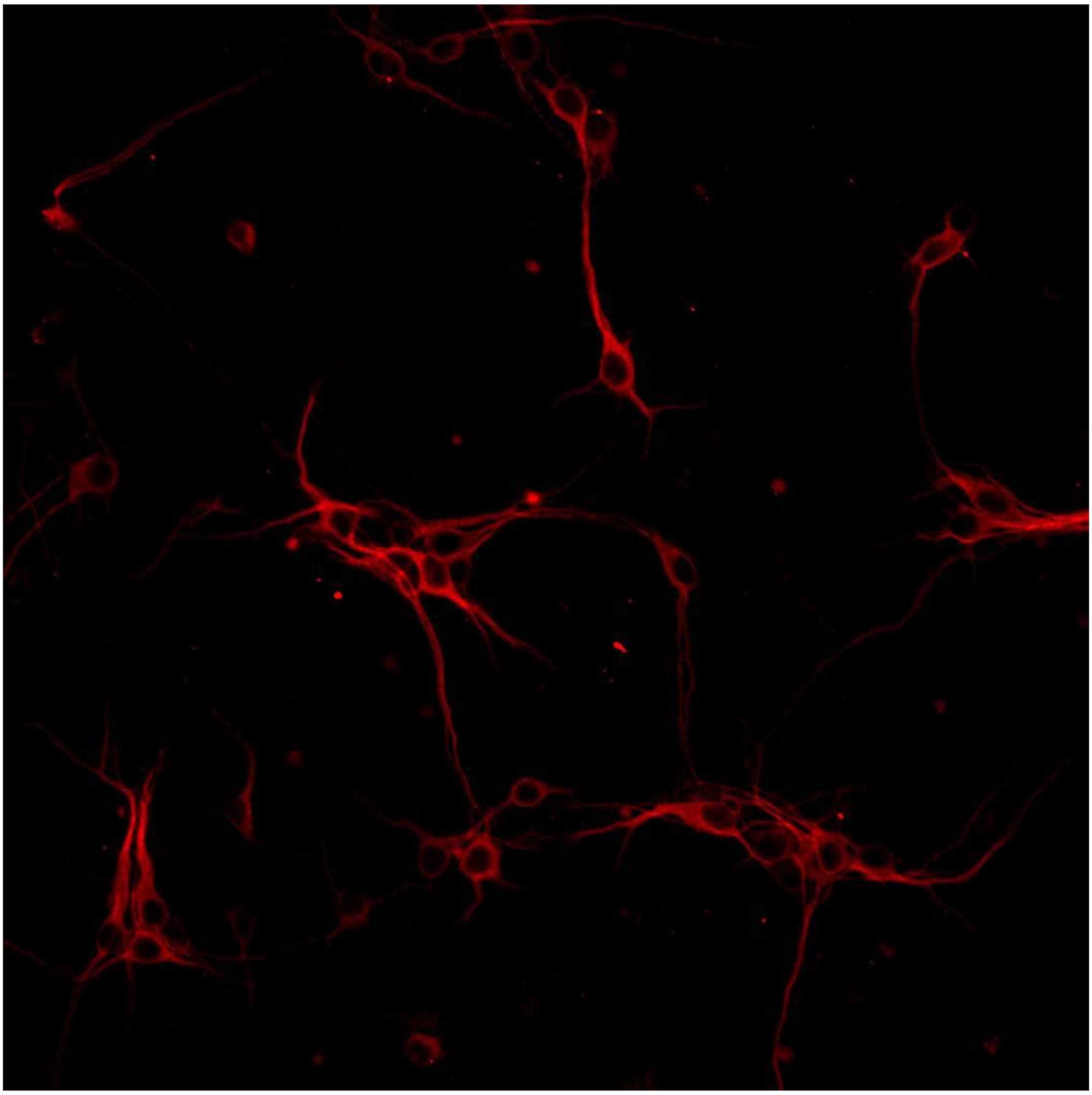

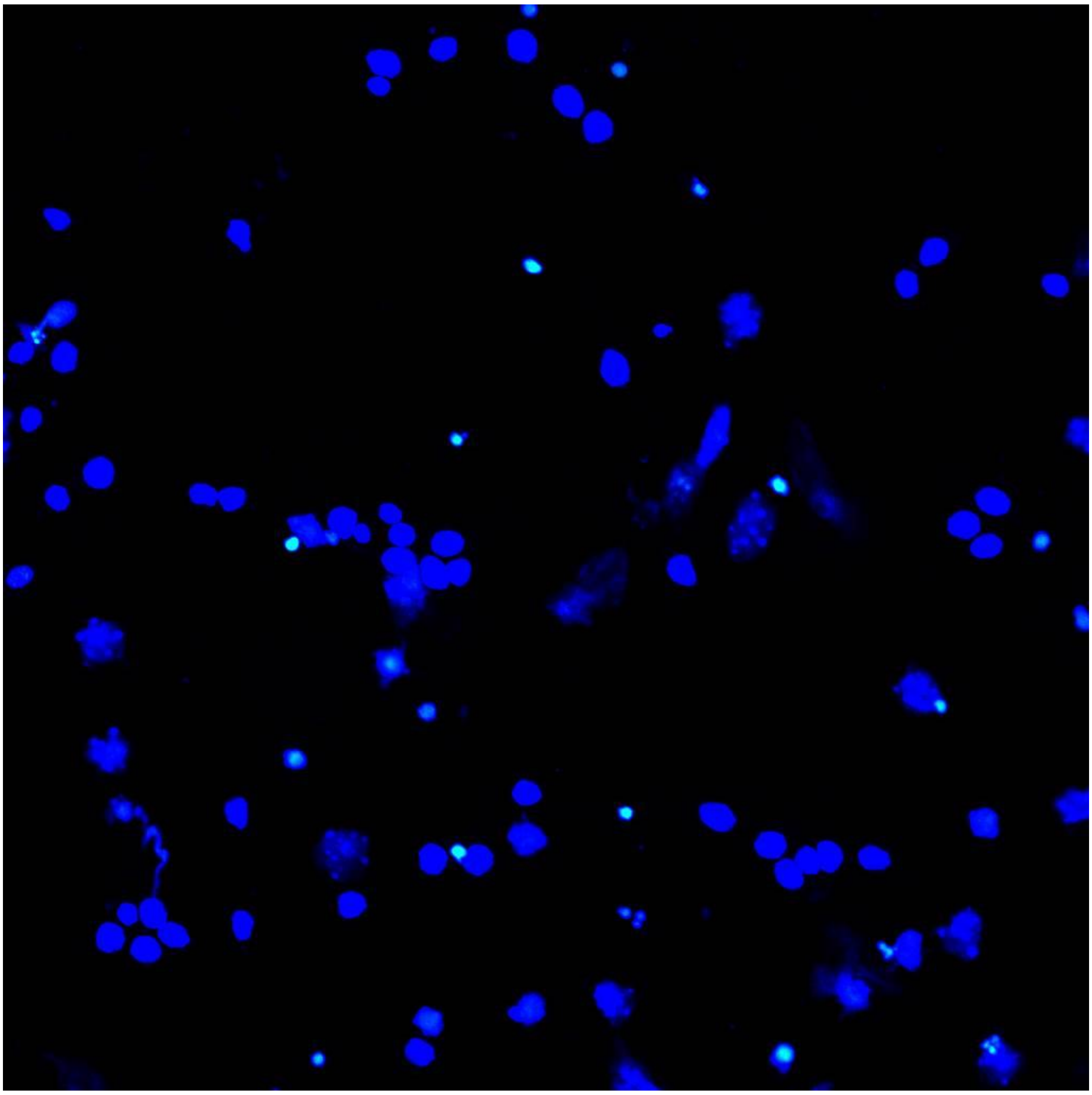

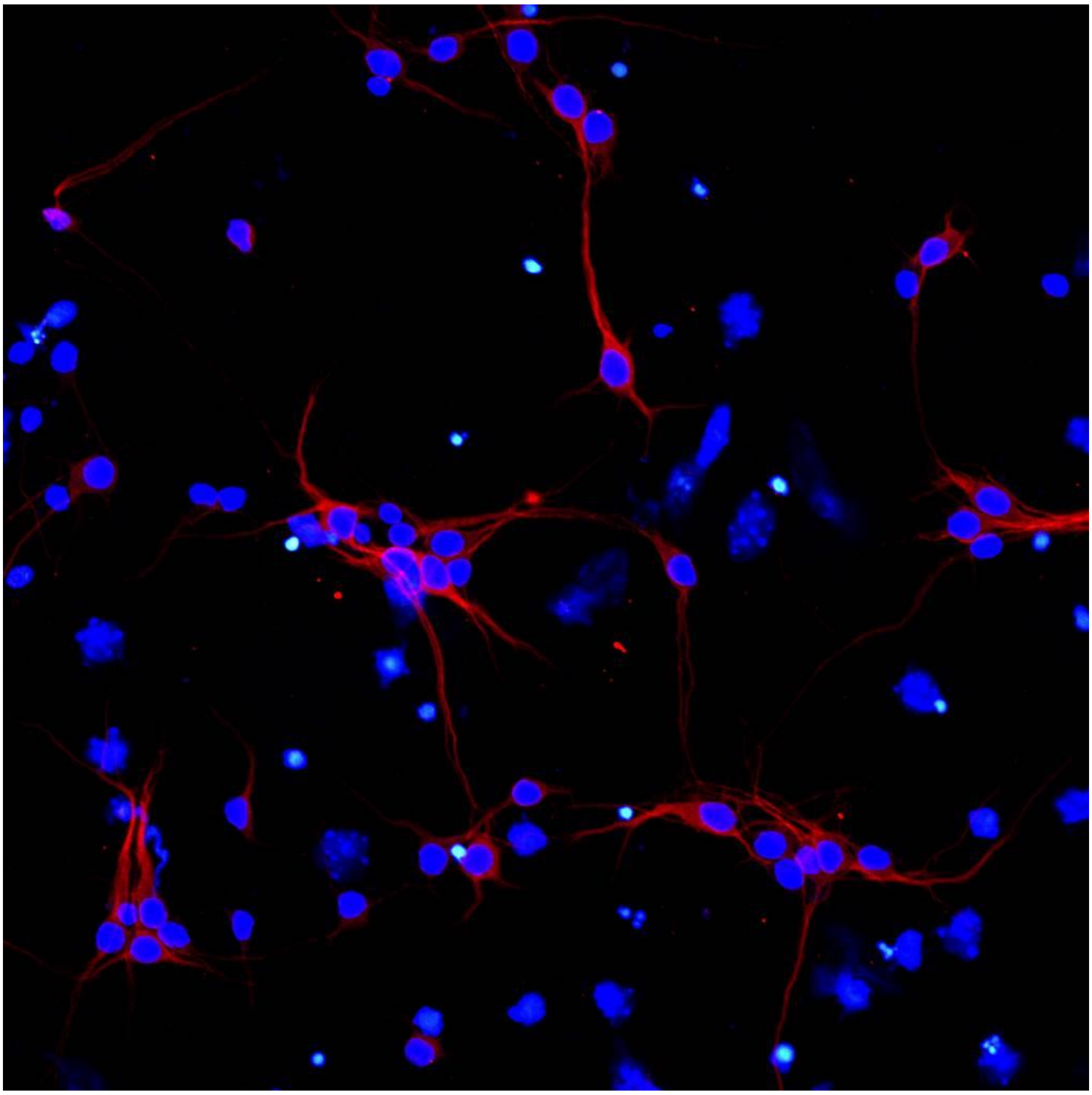

OGD/R+lv-ctrl+AST group

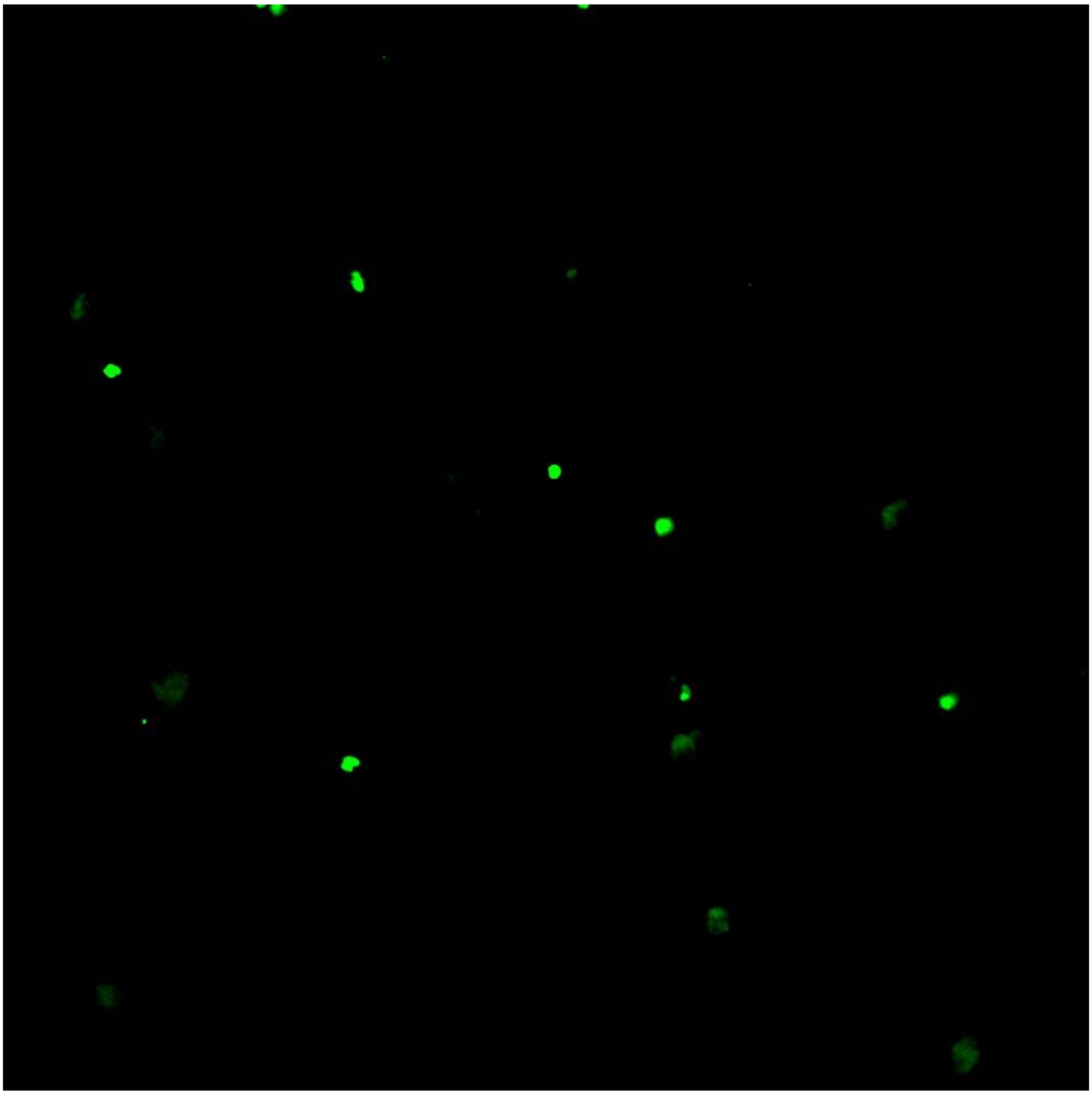

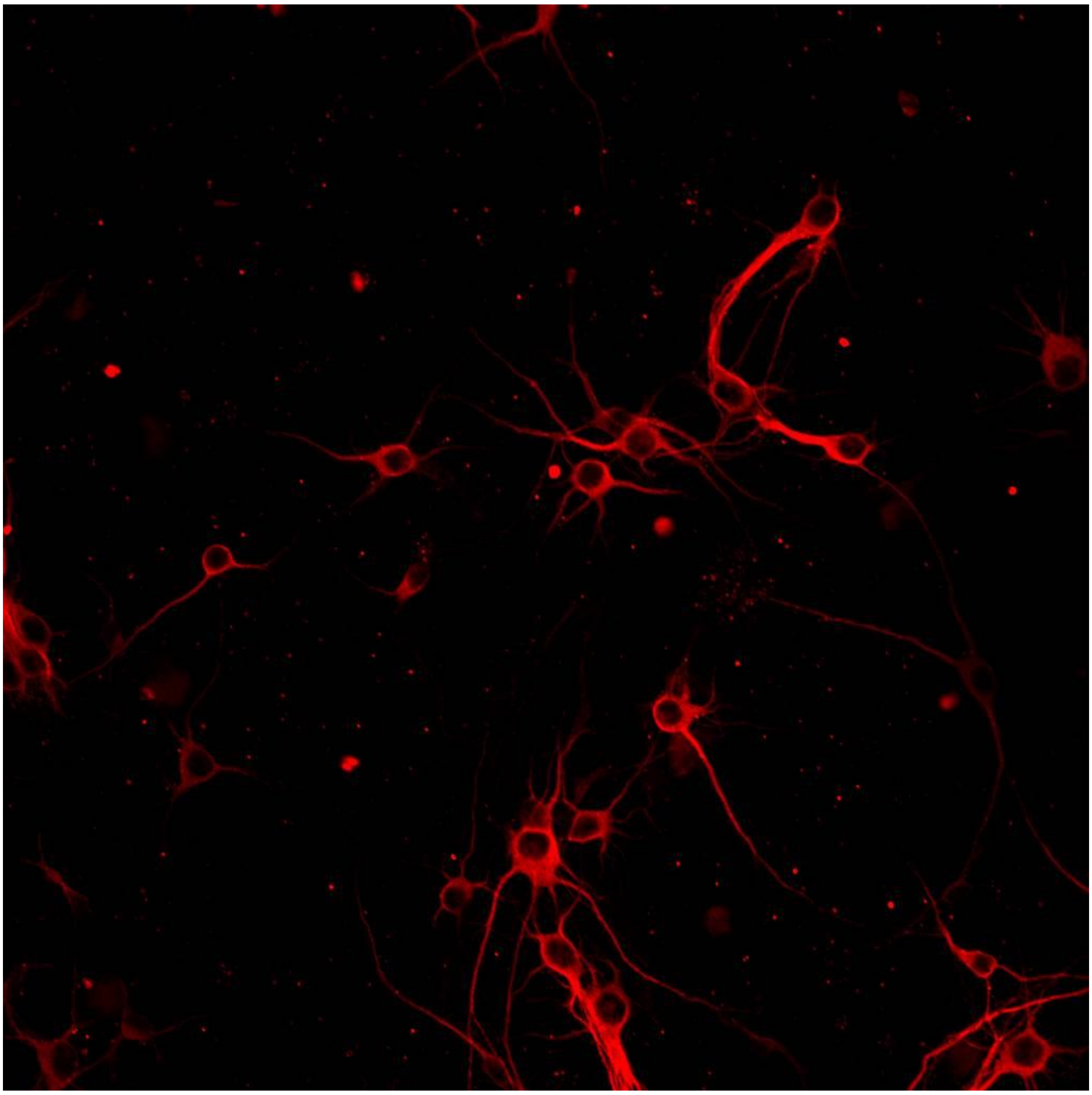

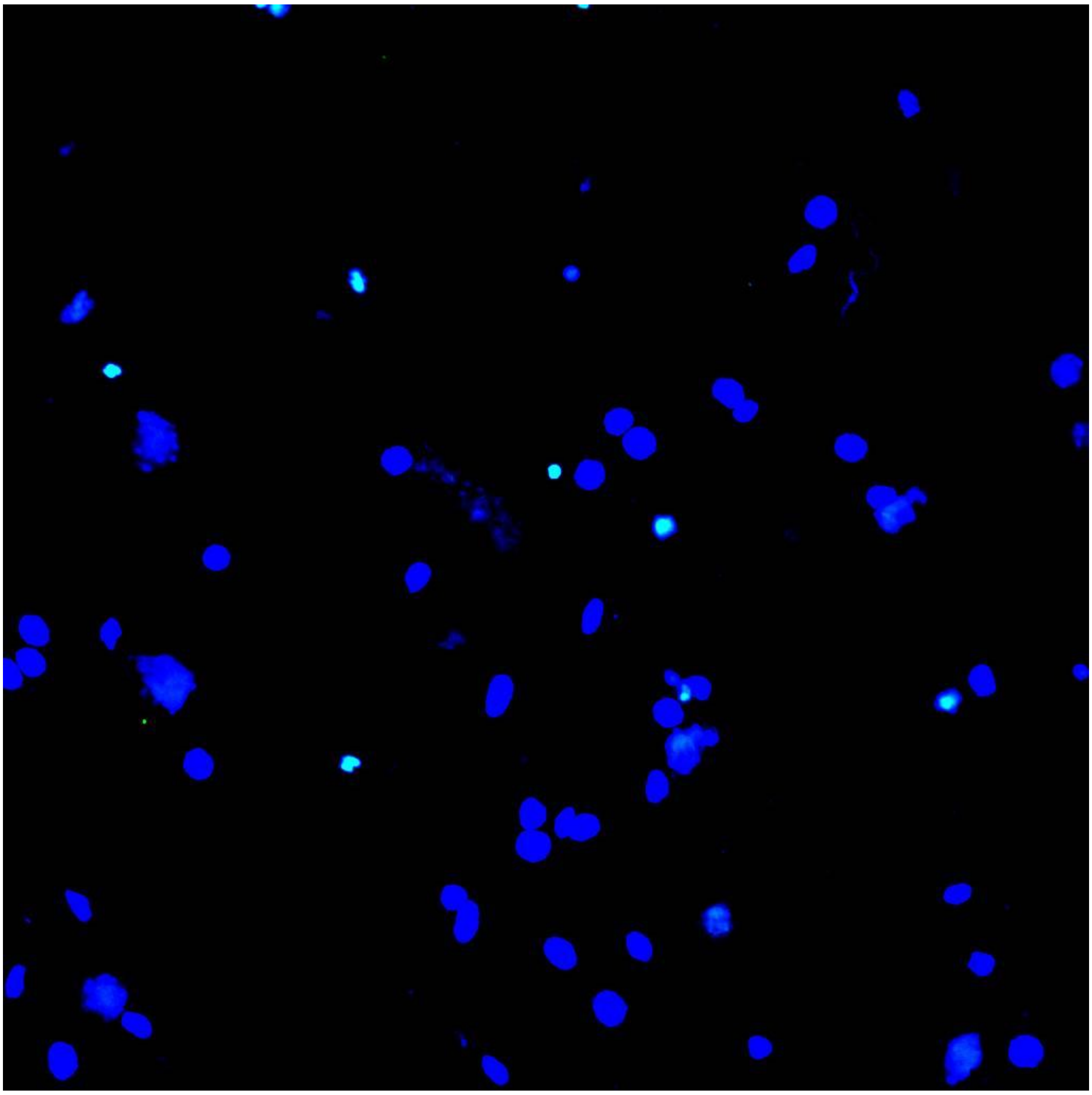

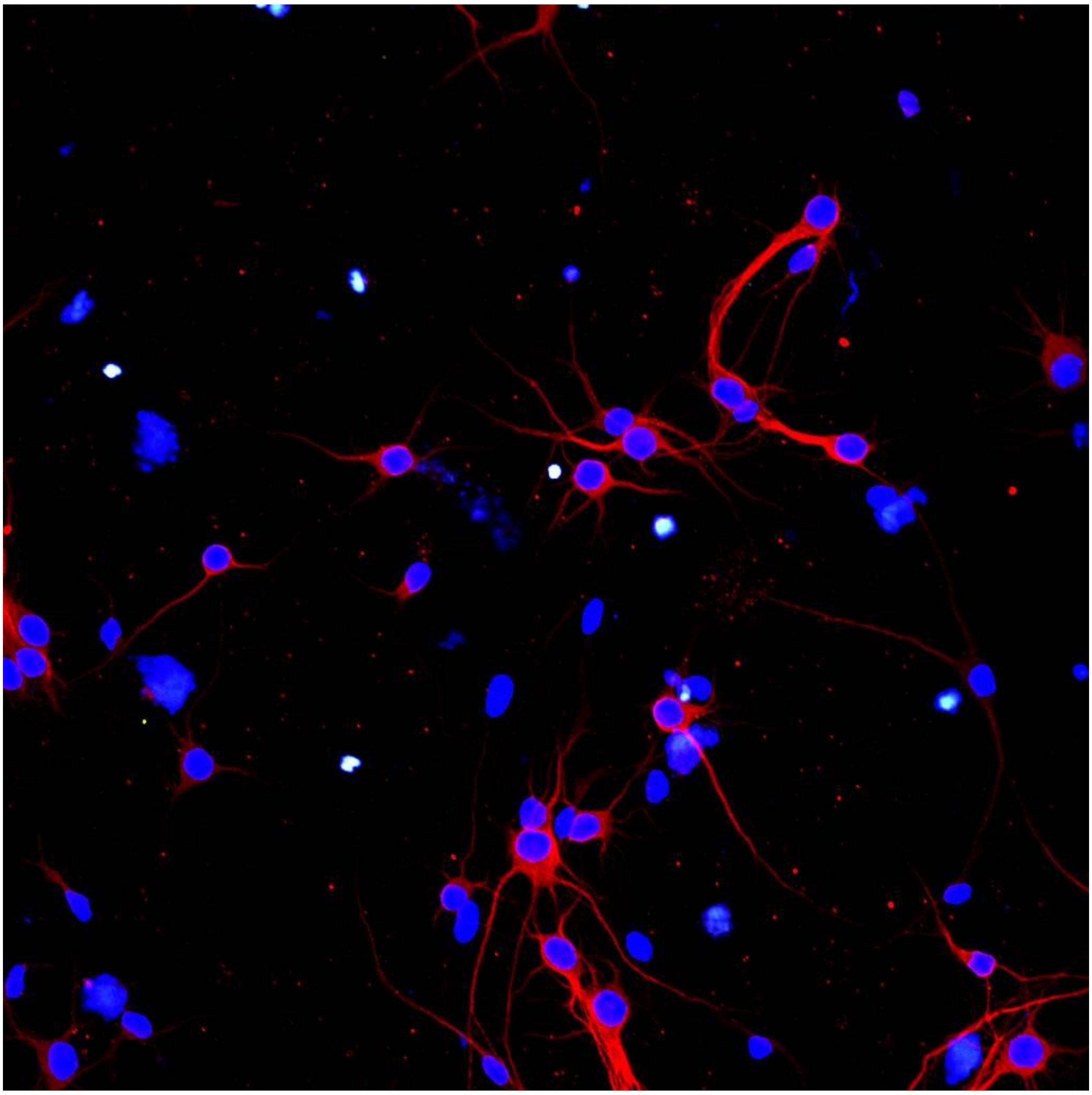

OGD/R+lv-Nrf2+AST group

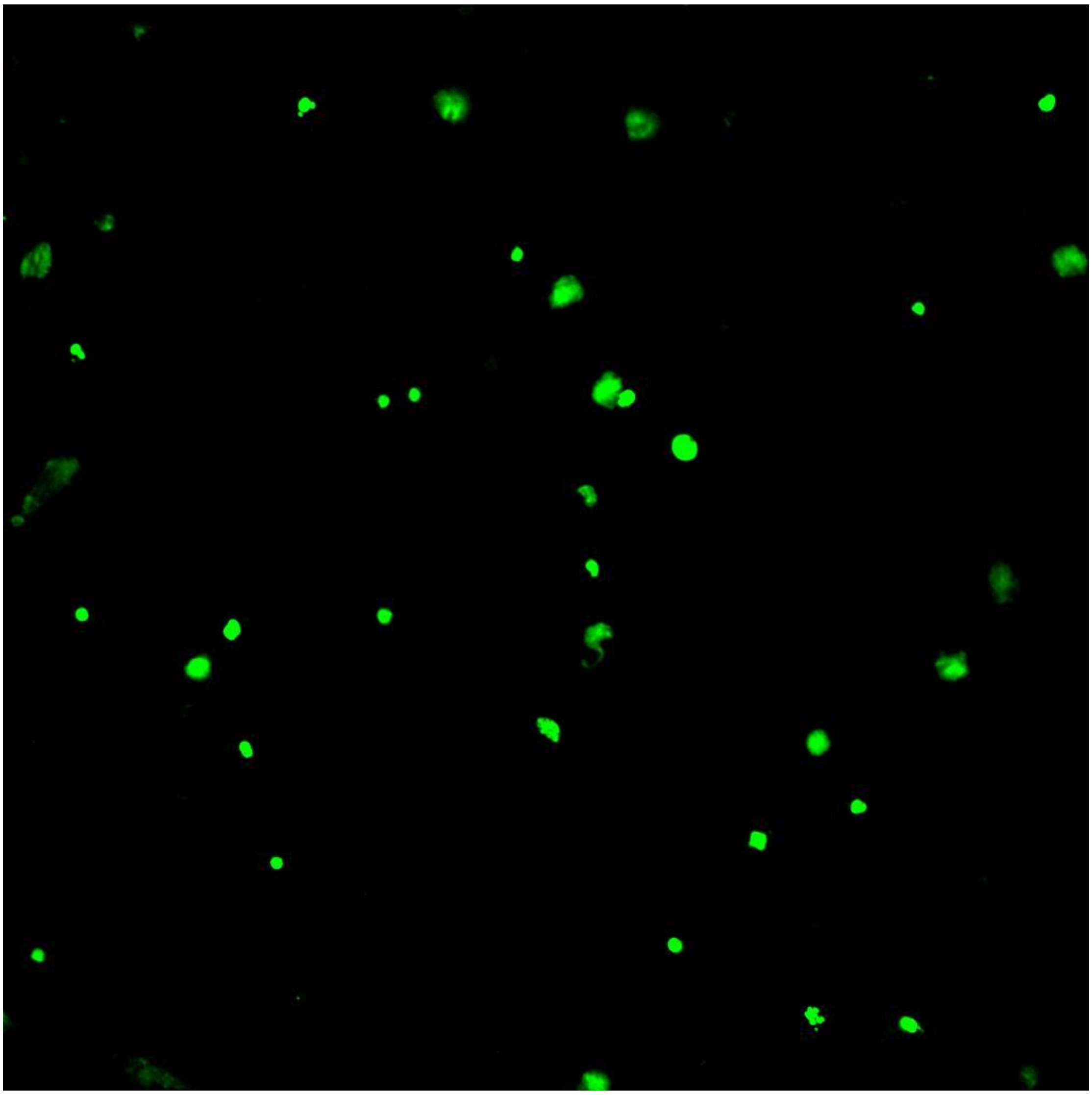

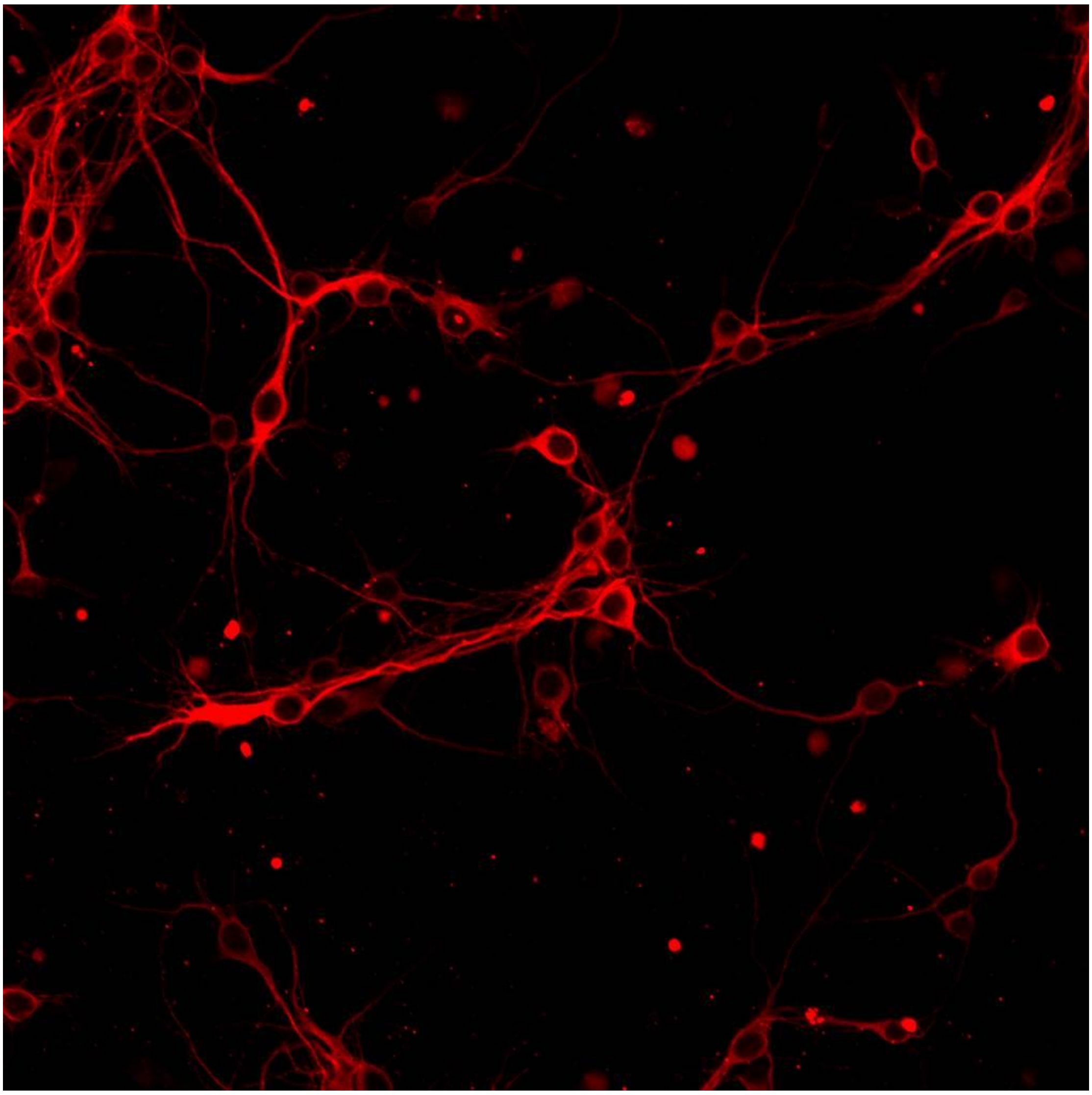

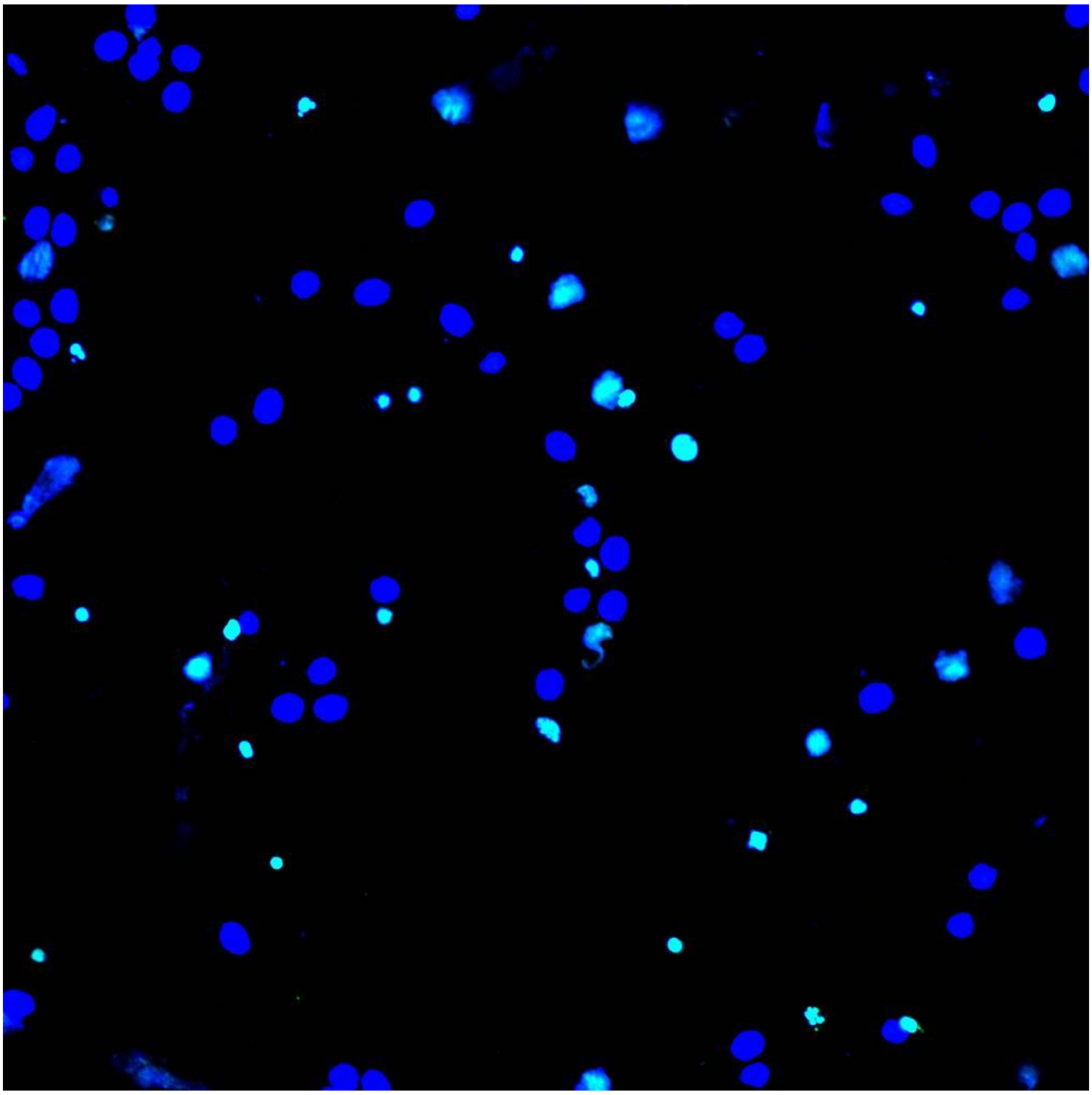

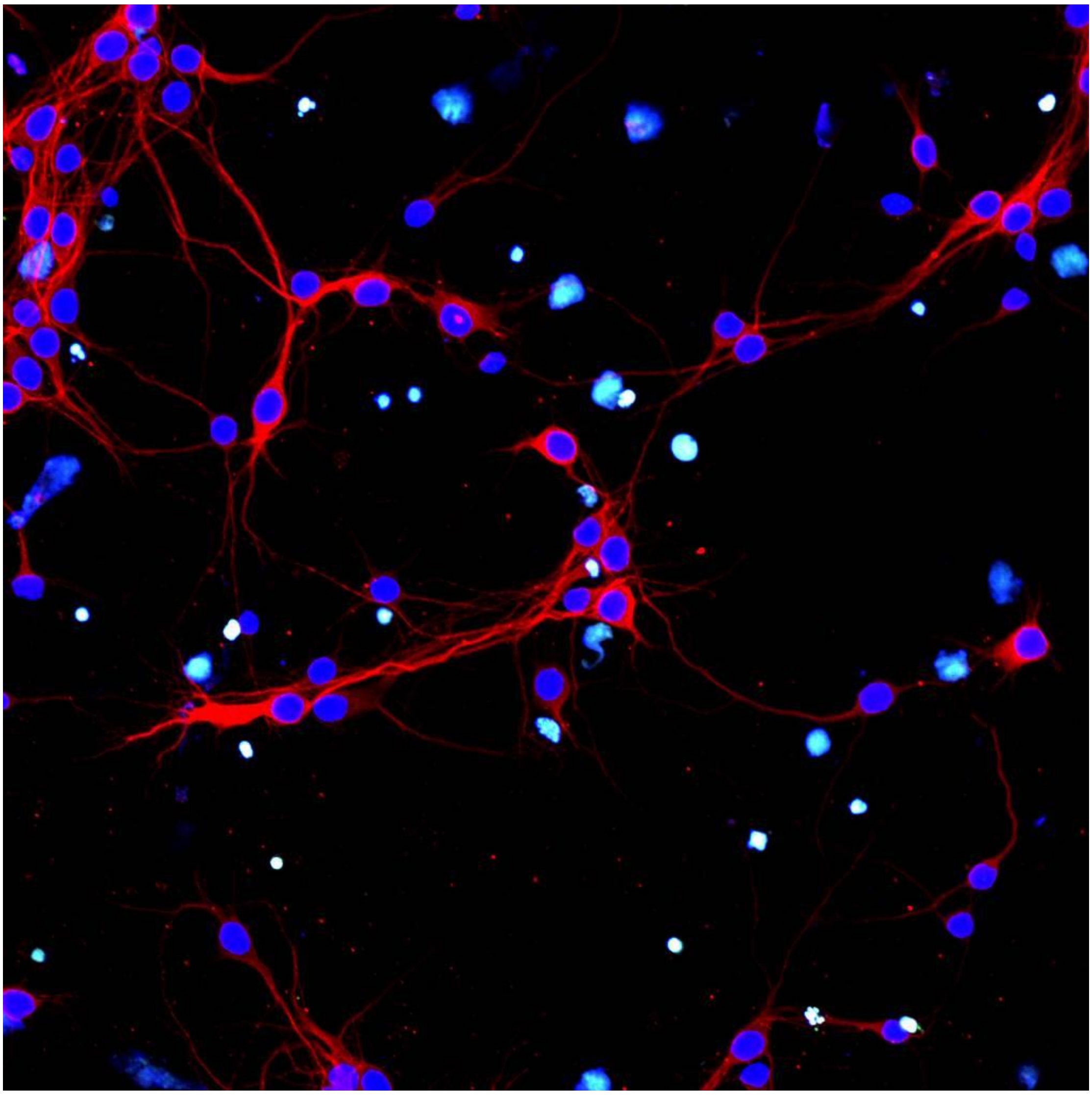

SFN group

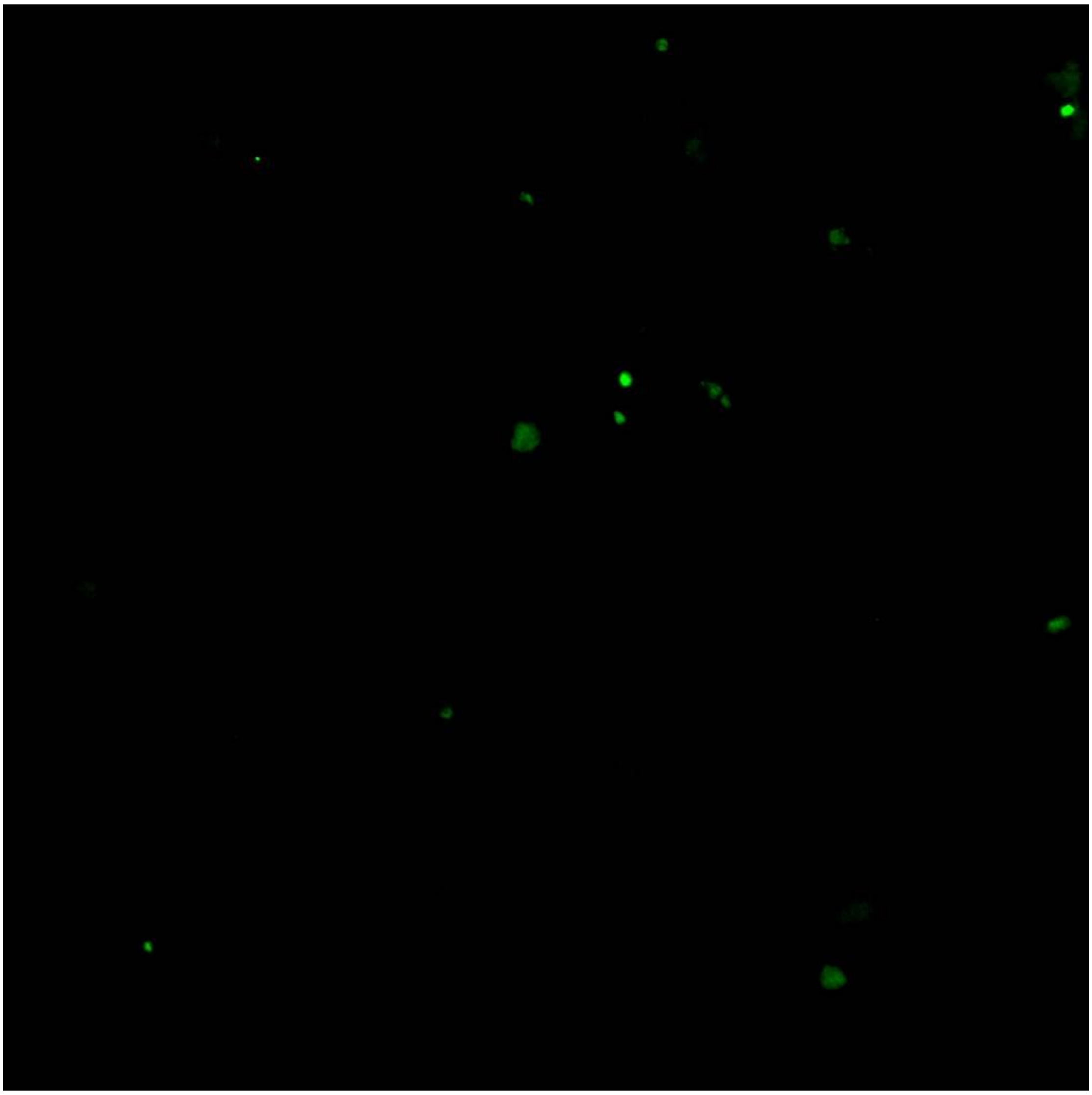

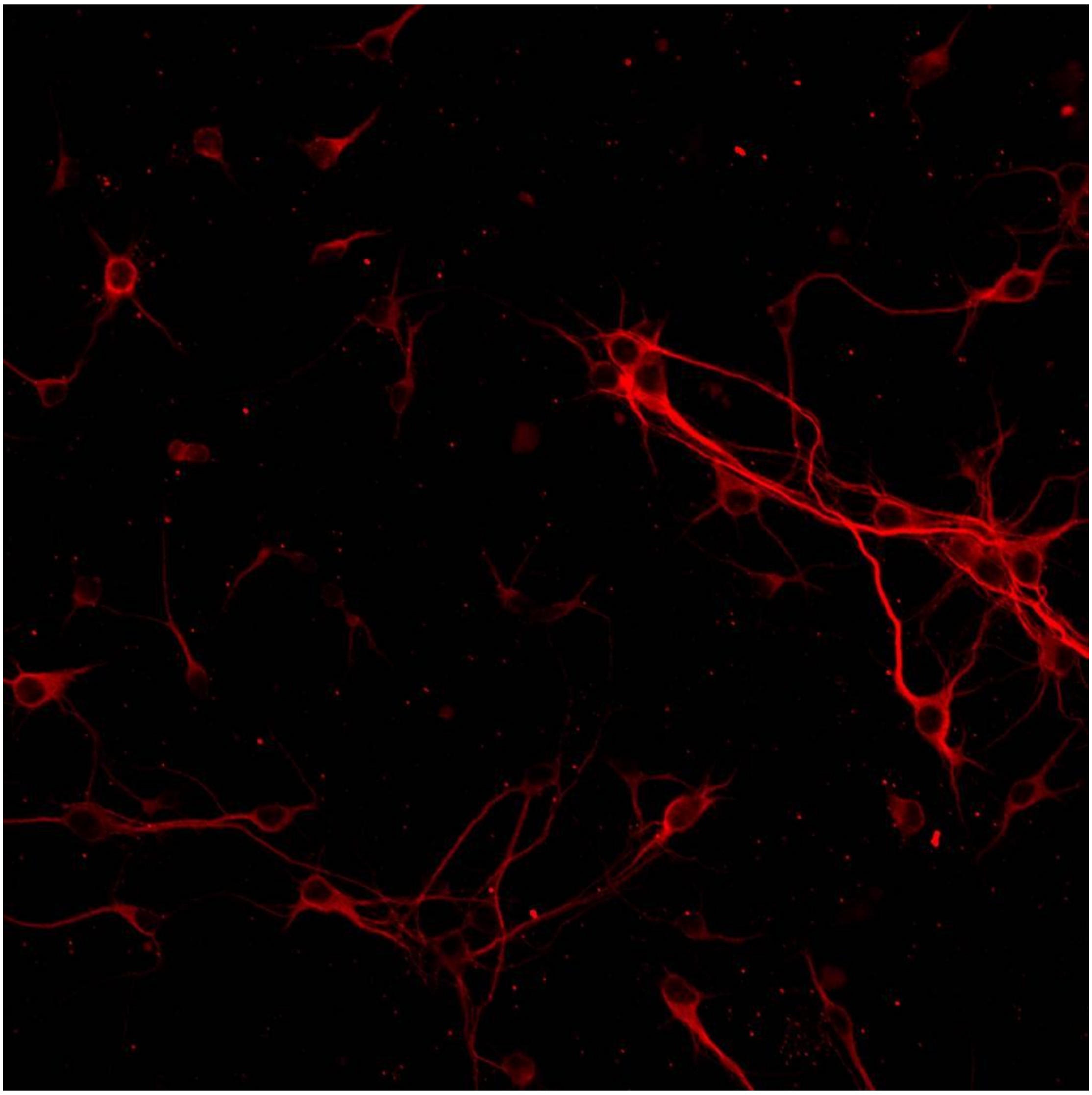

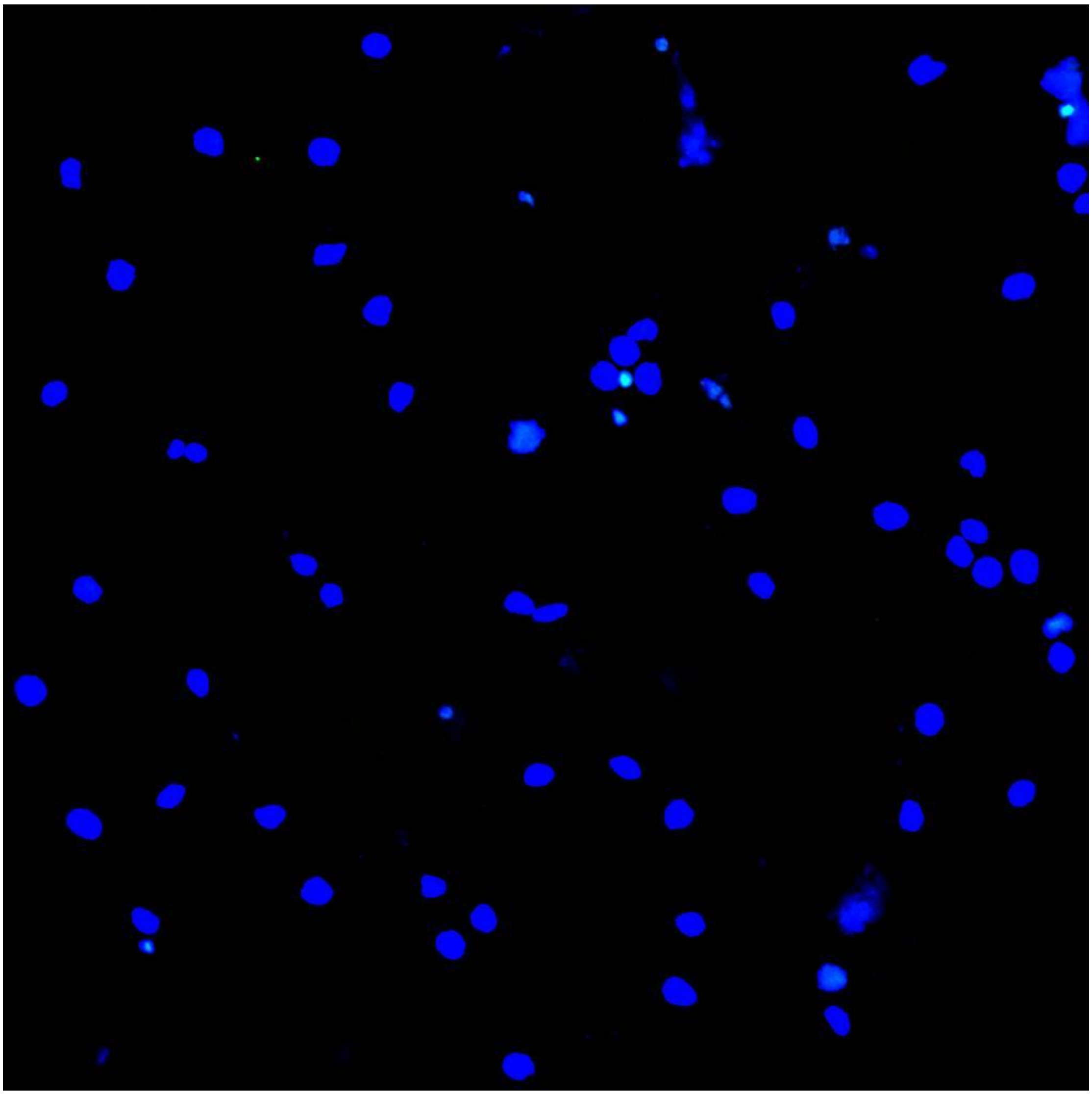

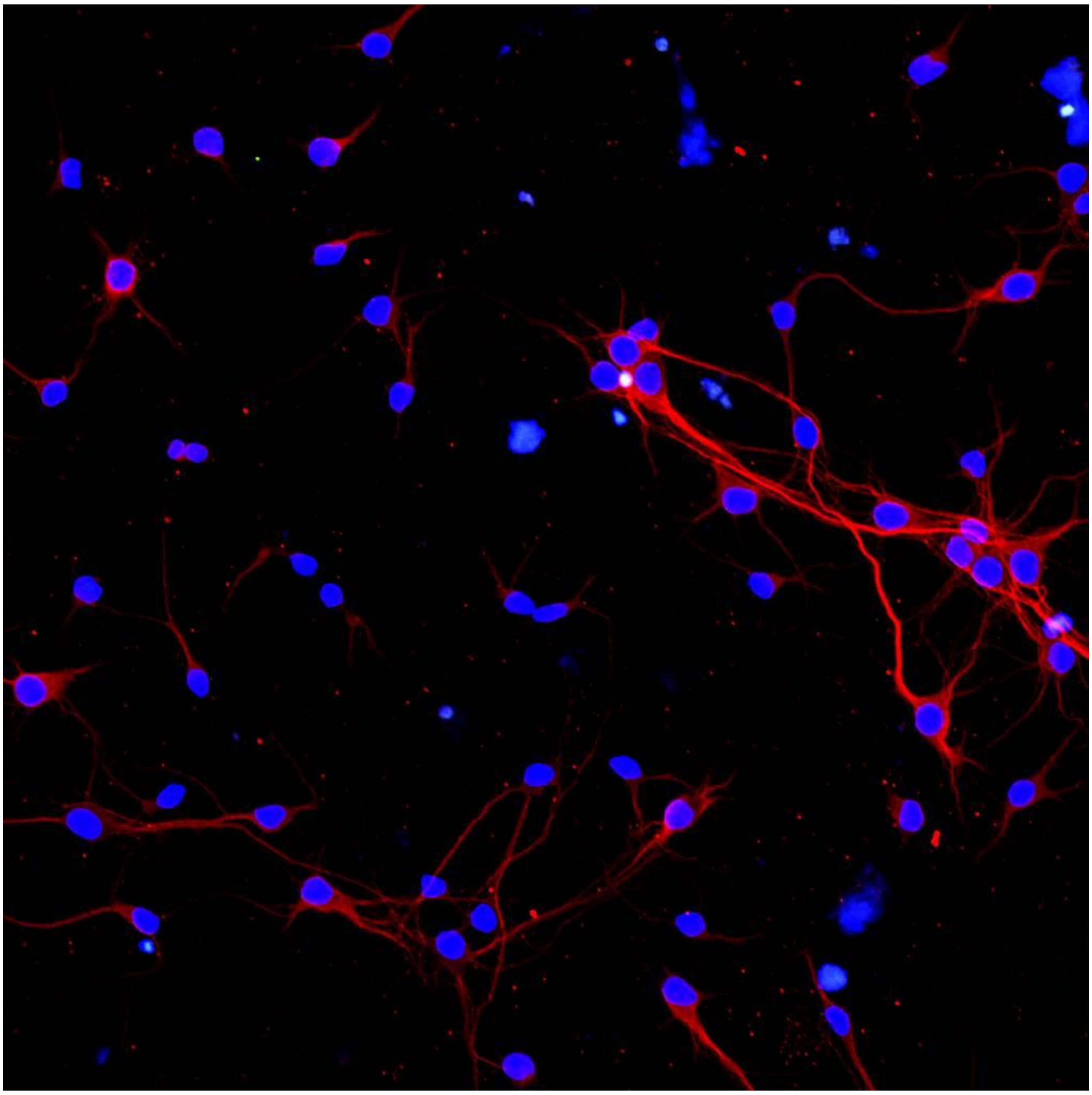

SFN+AST group

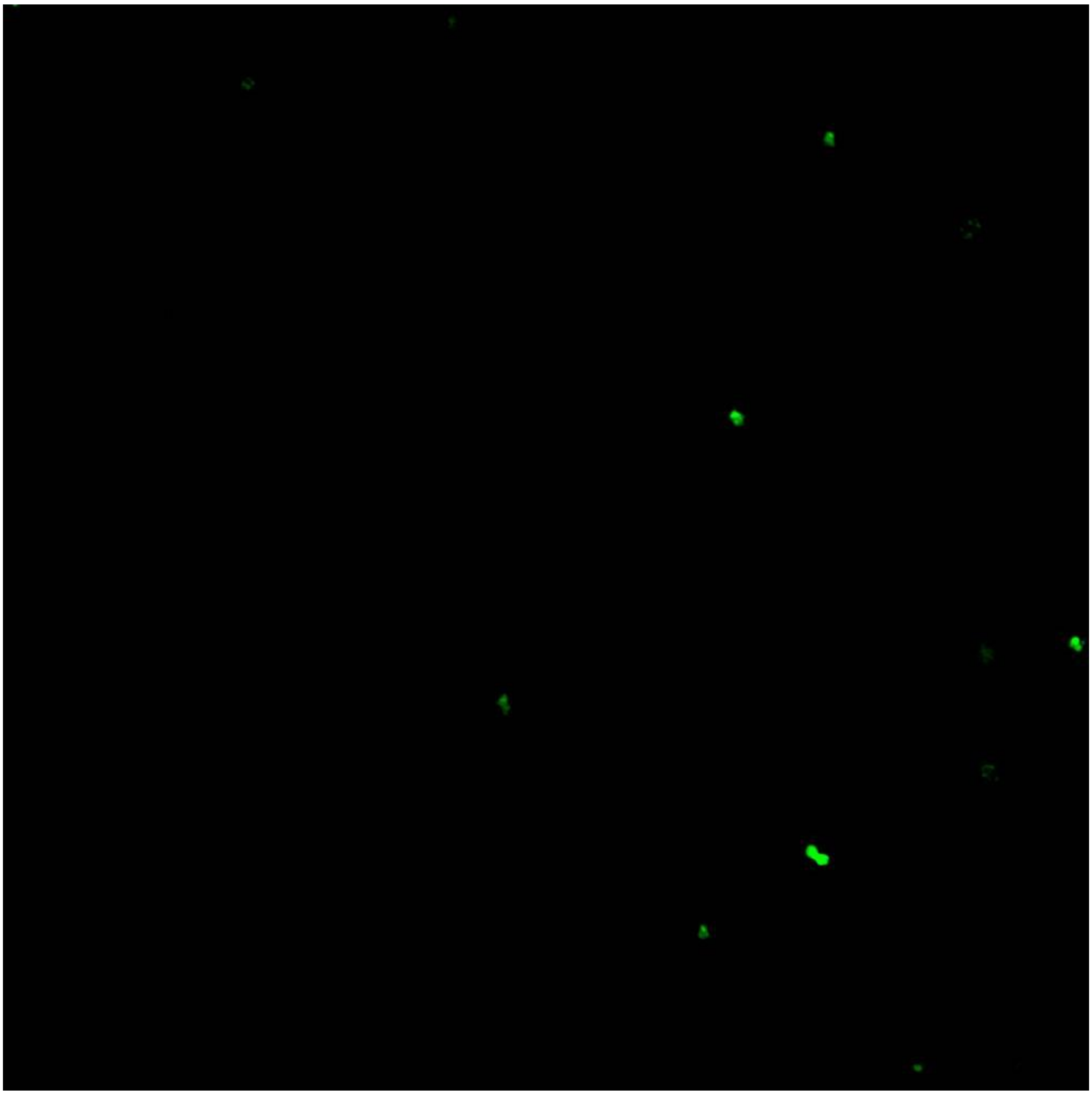

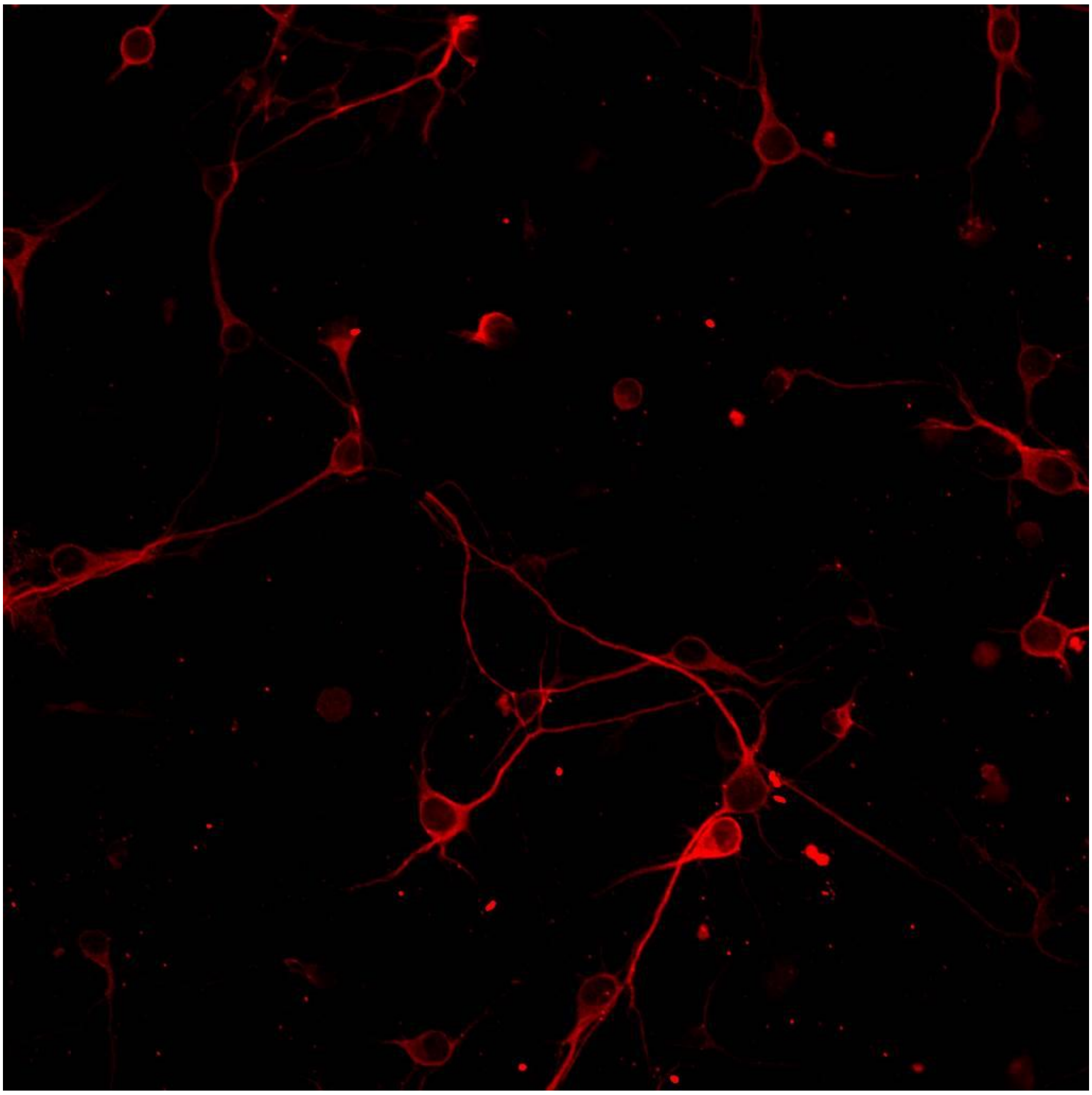

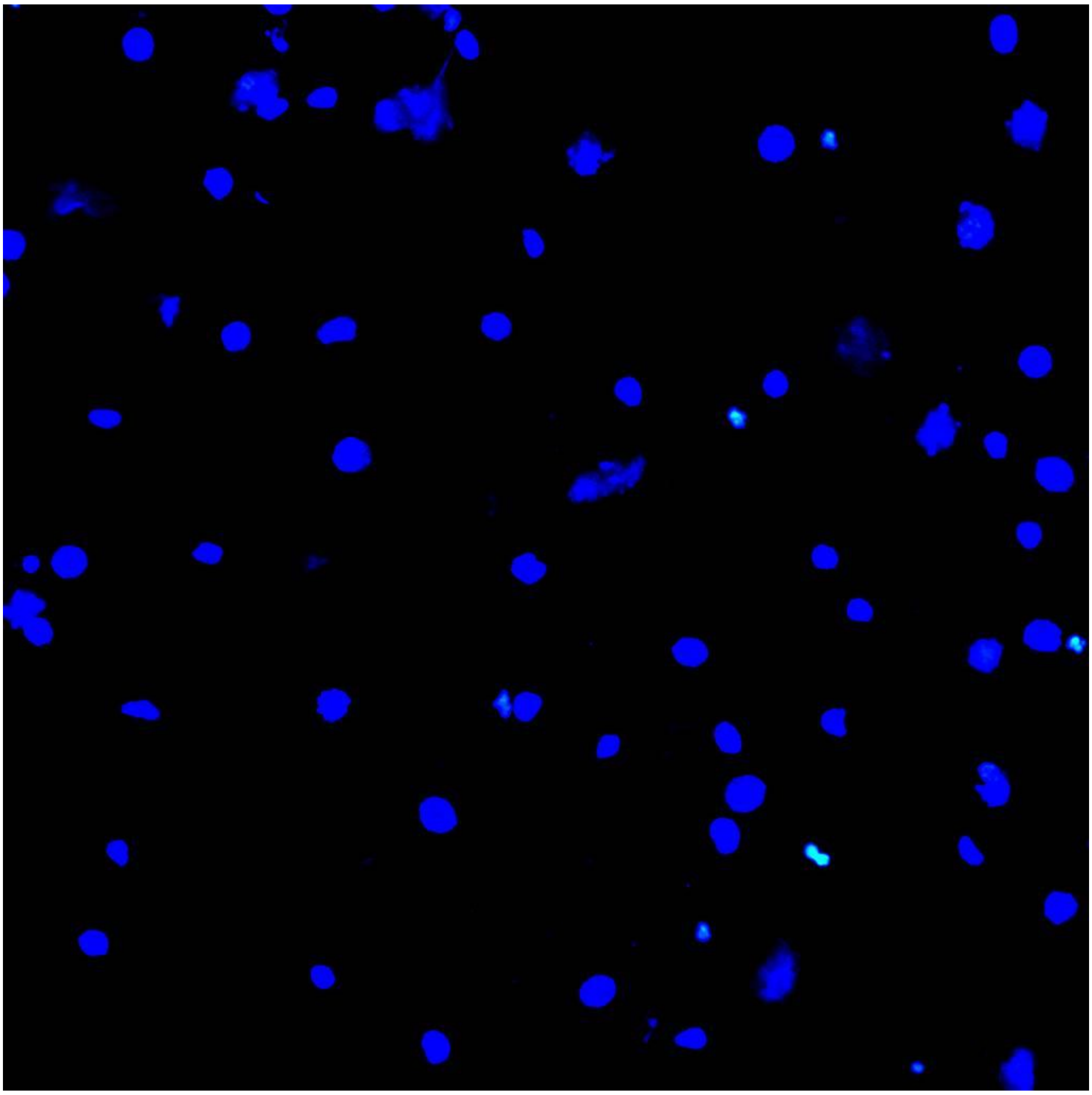

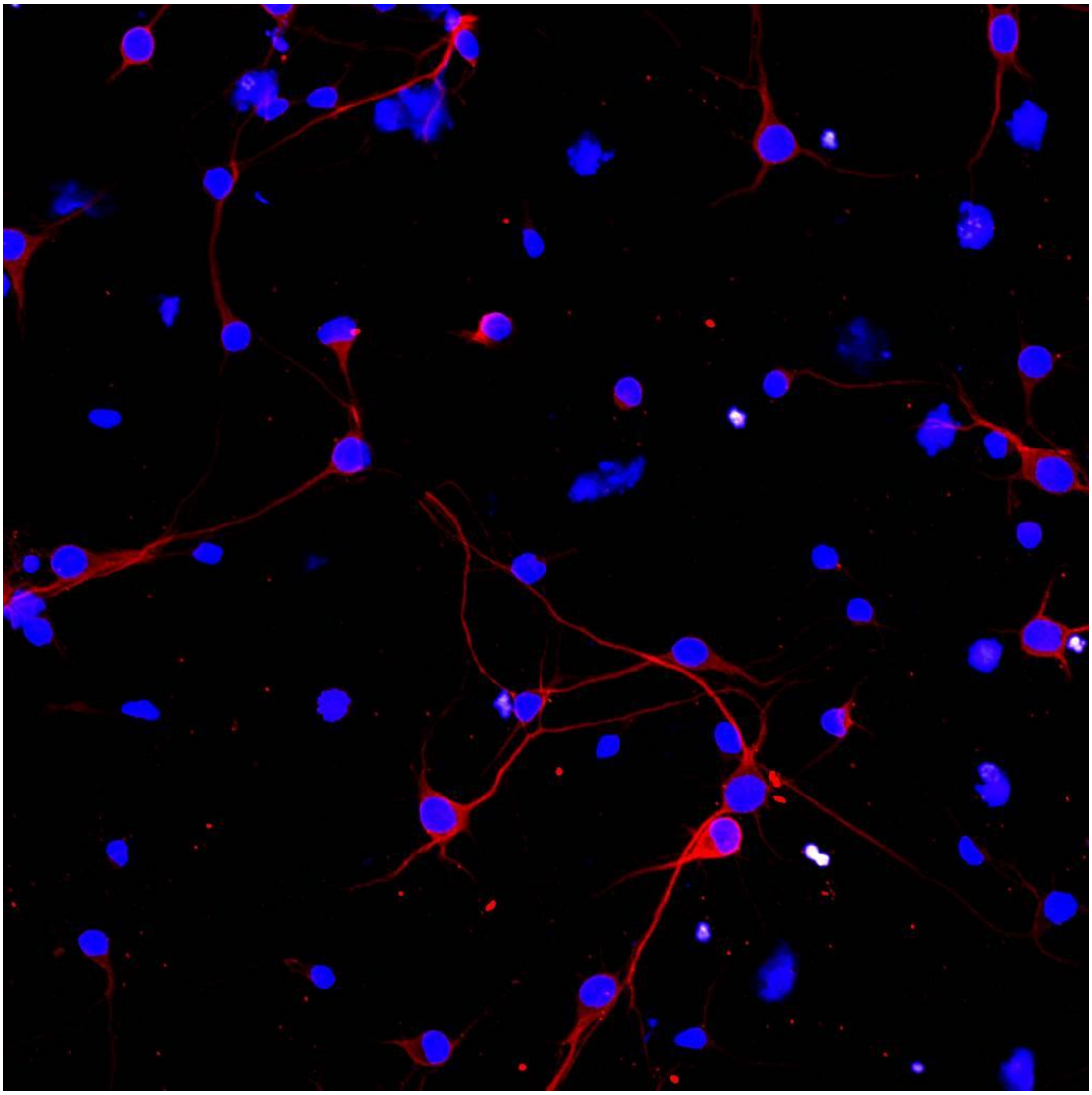

Supplement: Multimedia component 2 [file mmc2.pdf]
