# Supplementary material for: Astilbin exerts a neuroprotective effect by upregulating the signaling of nuclear NF-E2-related factor 2 in vitro
Source: Heliyon. 2024 Sep 3;10(17):e37276. doi: 10.1016/j.heliyon.2024.e37276 (PMC11409207; doi:10.1016/j.heliyon.2024.e37276)
Supplement: Multimedia component 3 [file mmc3.pdf]

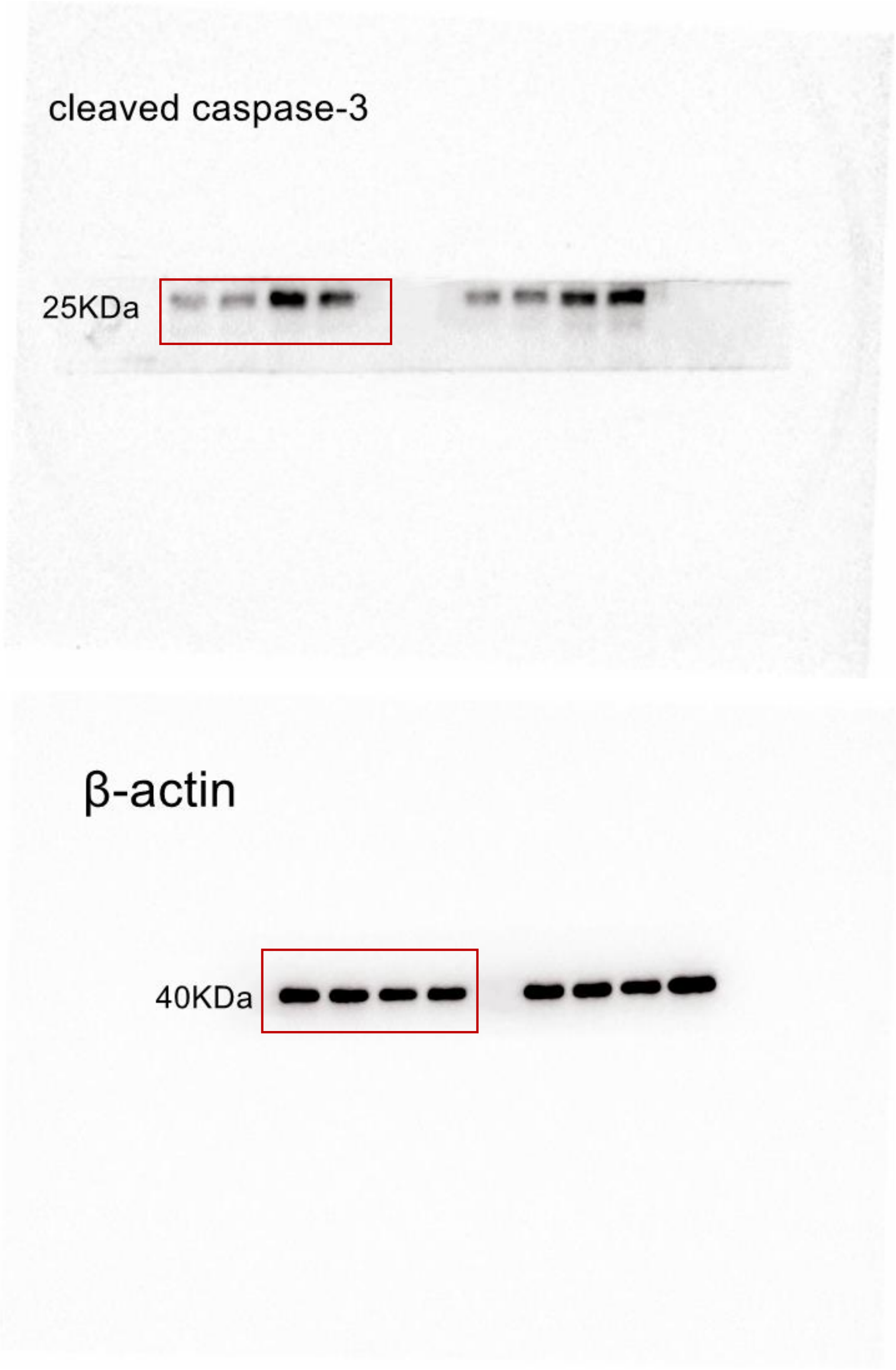
cleaved caspase-3
25KDa
β-actin
40KDa

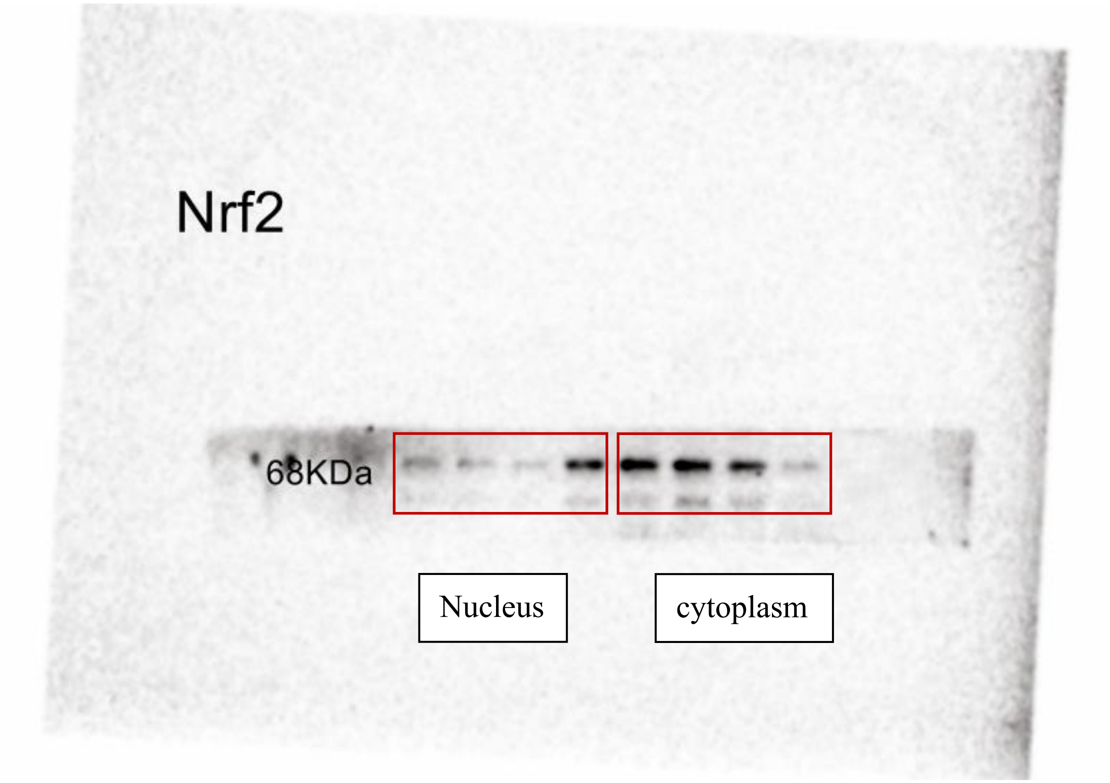
Nrf2
68KDa
Nucleus
cytoplasm

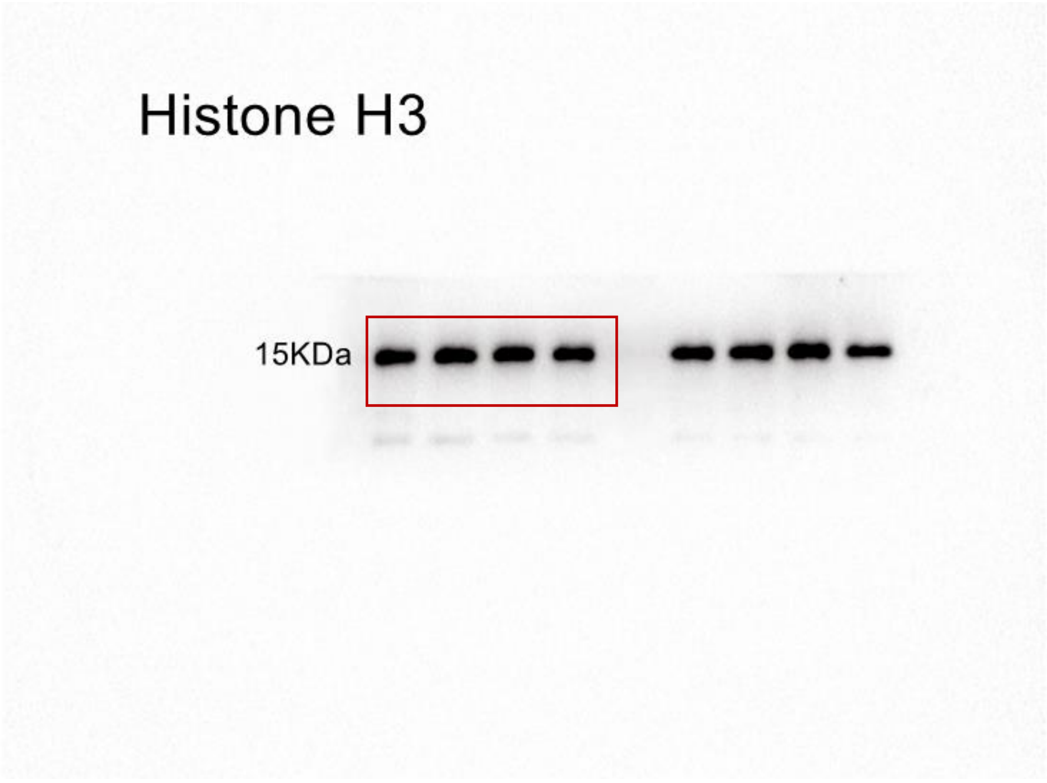
Histone H3
15KDa

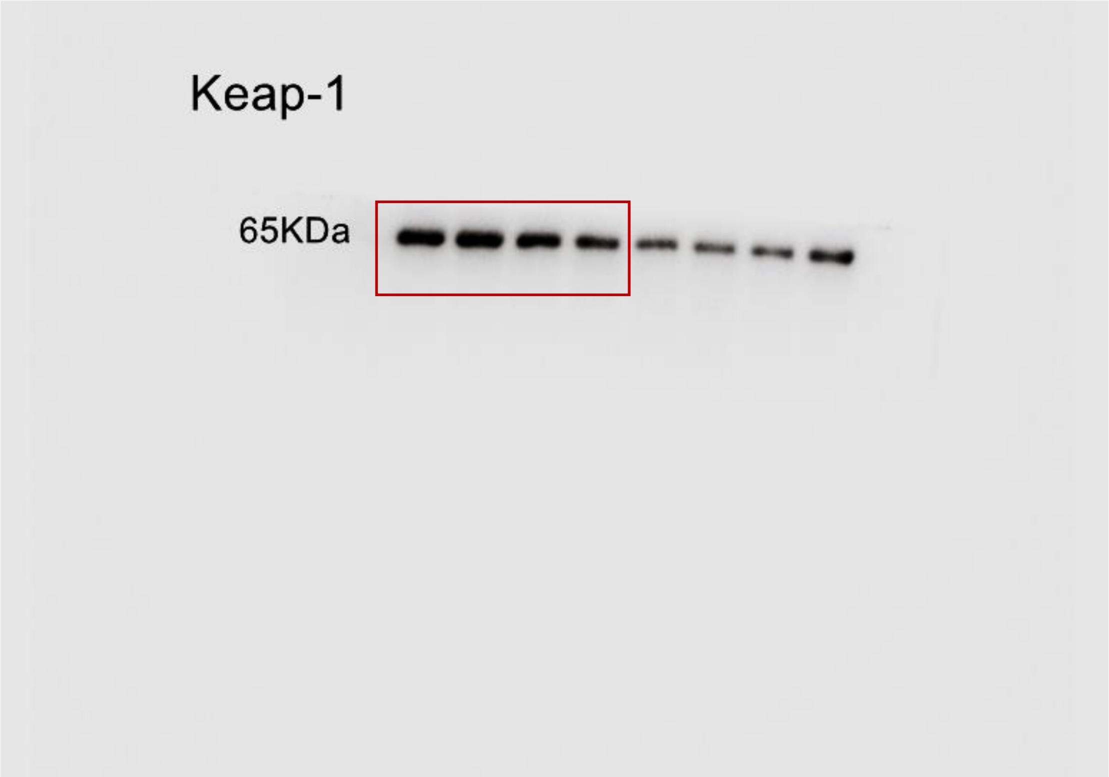
Keap-1
65KDa

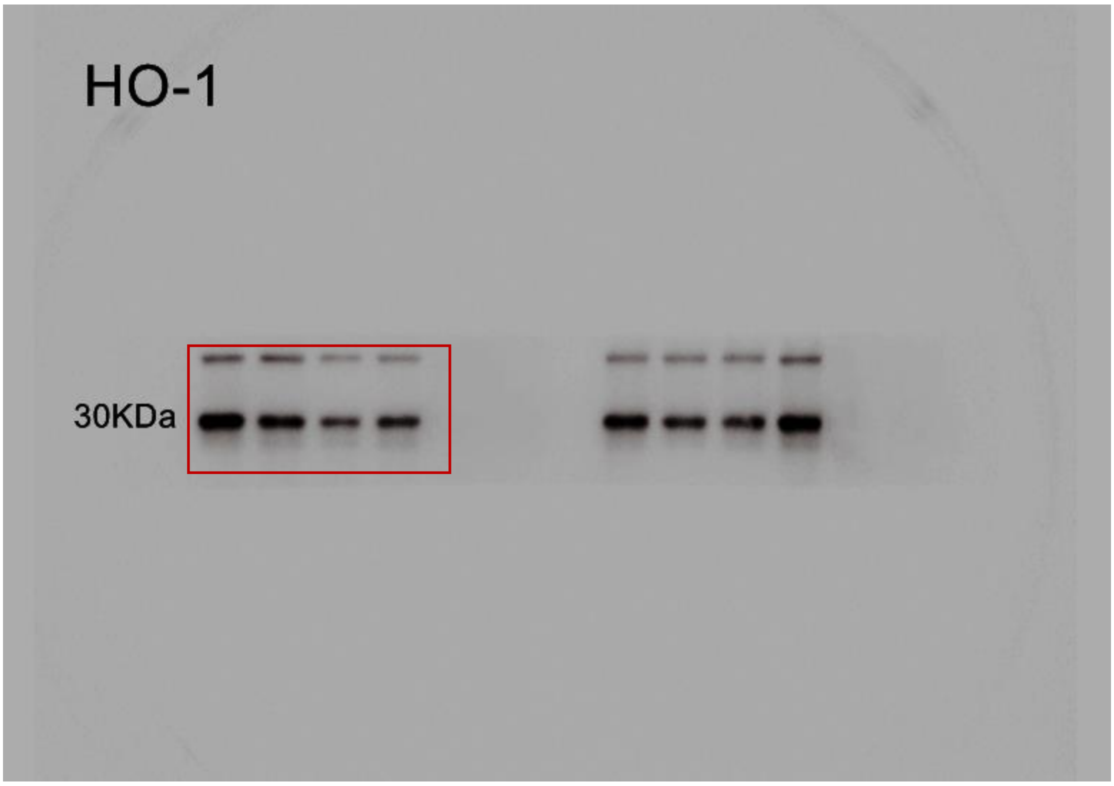
HO-1
30KDa

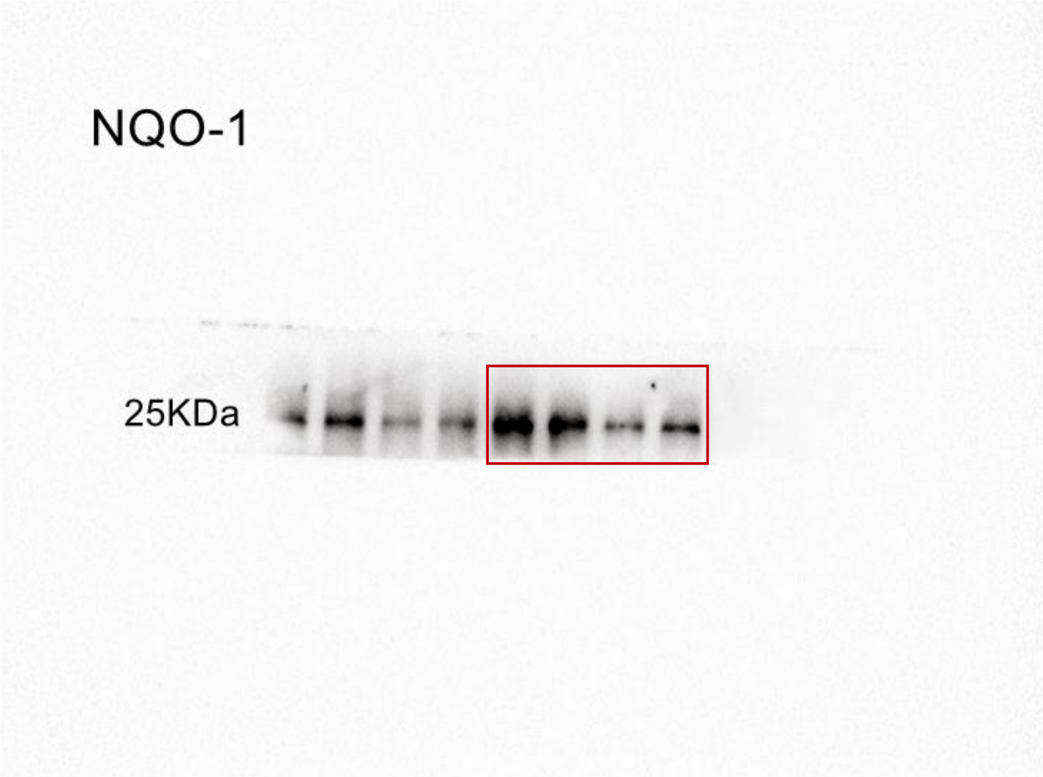
NQO-1
25KDa

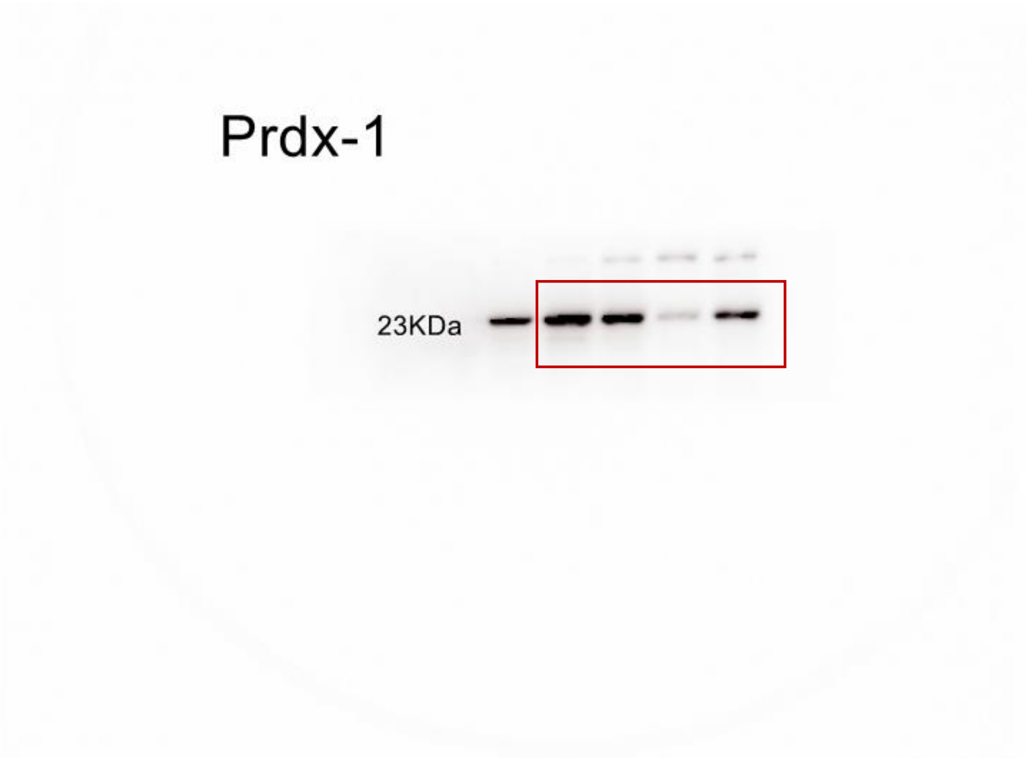
Prdx-1
23KDa

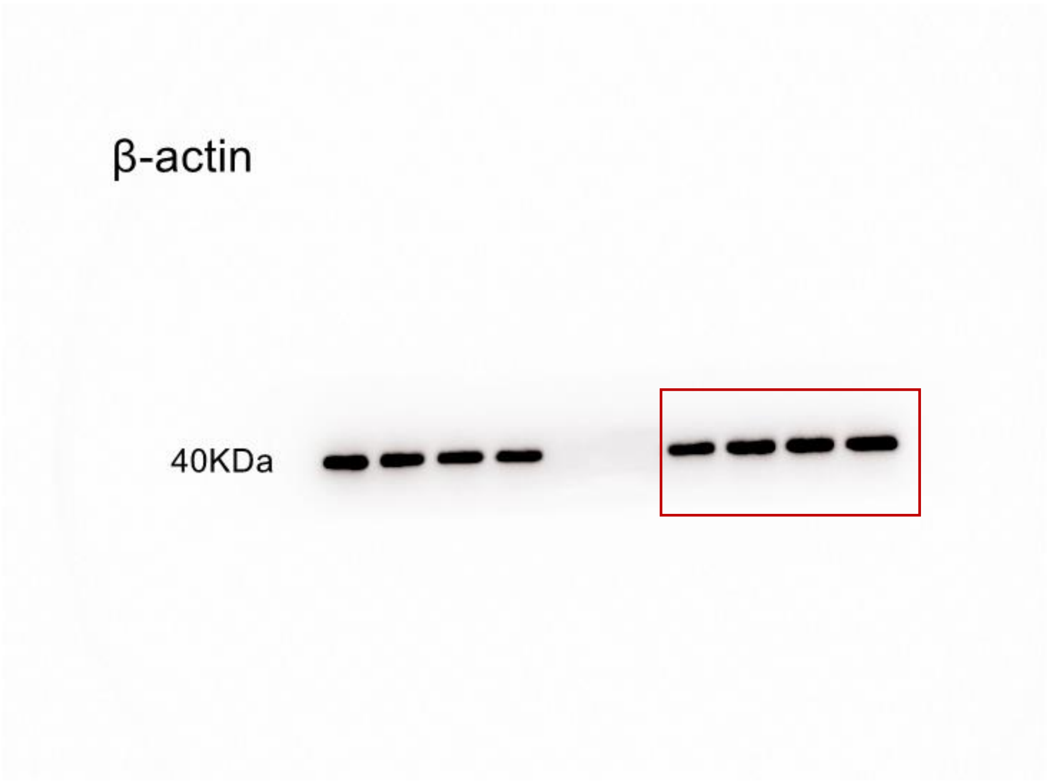
β-actin
40KDa

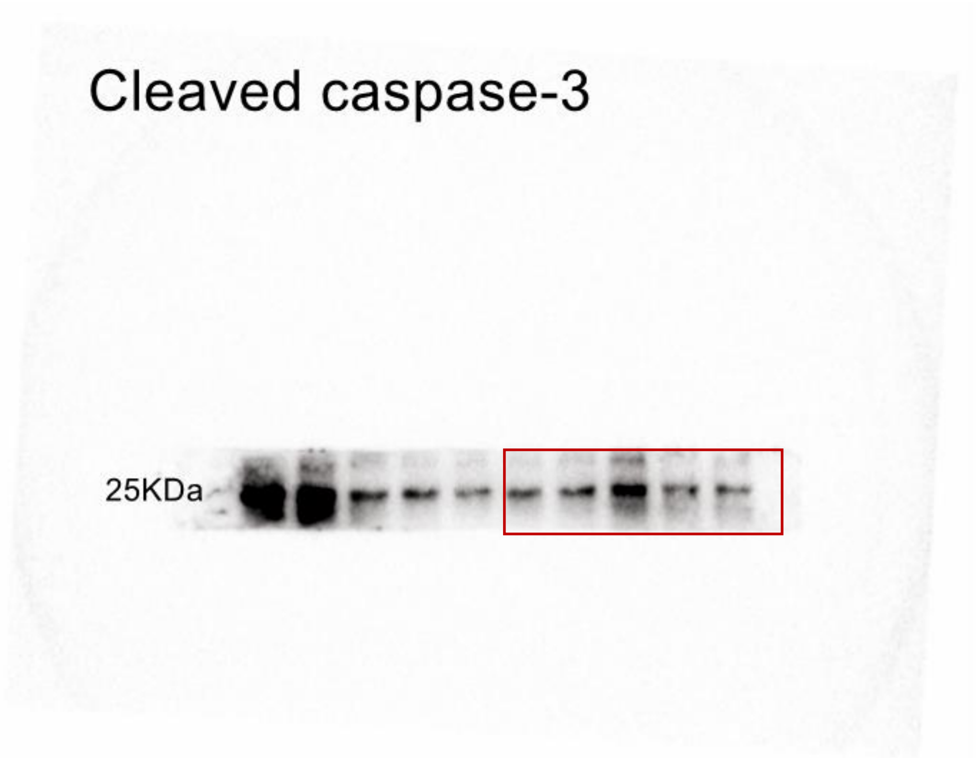
Cleaved caspase-3
25KDa

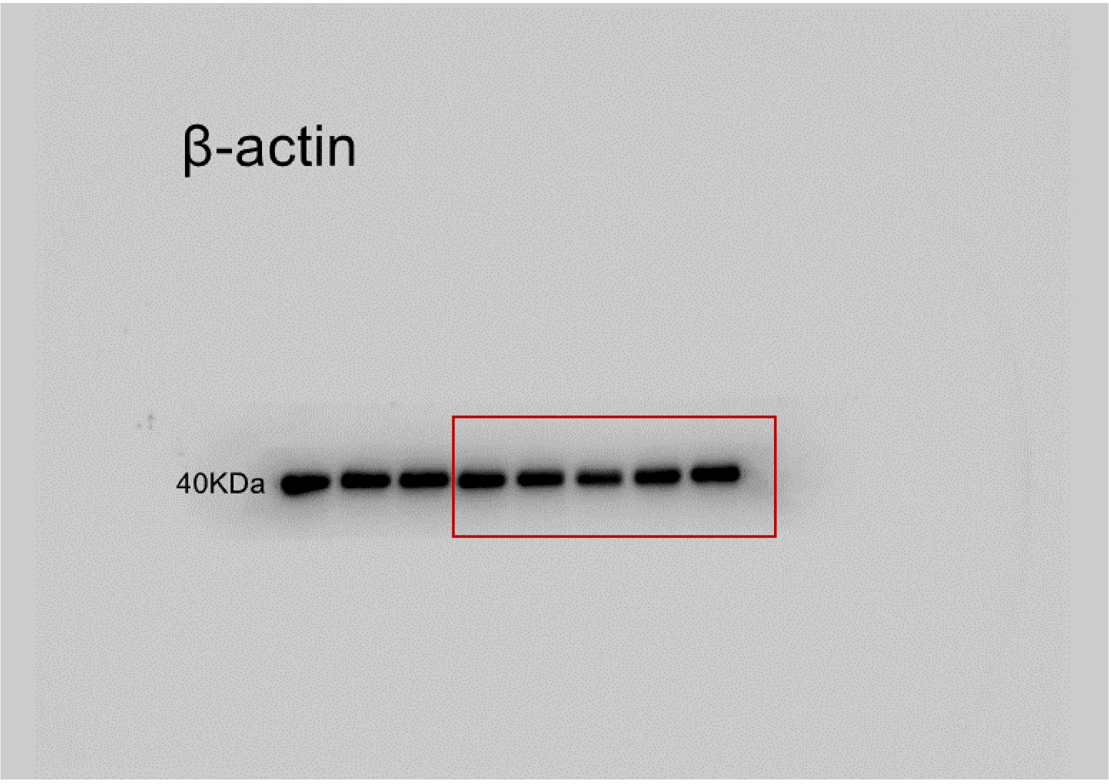
β-actin
40KDa
